# Single Cell Analysis Reveals Reciprocal Tumor-Macrophage Intercellular Communications Related with Metabolic Reprogramming in Stem-like Gastric Cancer

**DOI:** 10.3390/cells11152373

**Published:** 2022-08-02

**Authors:** Ji-Yong Sung, Jae-Ho Cheong

**Affiliations:** 1 Department of Laboratory Medicine, Yonsei University College of Medicine, Seoul 03722, Korea; 2 Department of Surgery, Yonsei University College of Medicine, Seoul 03722, Korea; 3 Yonsei Biomedical Research Institute, Yonsei University College of Medicine, Seoul 03722, Korea; 4 Department of Biochemistry and Molecular Biology, Yonsei University College of Medicine, Seoul 03722, Korea

**Keywords:** immunometabolism, single cell, gastric cancer, glycan metabolism, cancer stemness

## Abstract

Metabolic alterations and direct cell–cell interactions in the tumor microenvironment (TME) affect the prognostic molecular landscape of tumors; thus, it is imperative to investigate metabolic activity at the single-cell level rather than in bulk samples to understand the high-resolution mechanistic influences of cell-type specific metabolic pathway alterations on tumor cells. To investigate tumor metabolic reprogramming and intercellular communication at the single-cell level, we analyzed eighty-four metabolic pathways, seven metabolic signatures, and tumor-stroma cell interaction using 21,084 cells comprising gastric cancer and paired normal tissue. High EMT-score cells and stem-like subtype tumors showed elevated glycosaminoglycan metabolism, which was associated with poor patient outcome. Adenocarcinoma and macrophage cells had higher reactive oxidative species levels than the normal controls; they largely constituted the highest stemness cluster. They were found to reciprocally communicate through the common ligand RPS19. Consequently, ligand-target regulated transcriptional reprogramming resulted in *HS6ST2* expression in adenocarcinoma cells and *SERPINE1* expression in macrophages. Gastric cancer patients with increased *SERPINE1* and *HS6ST2* expression had unfavorable prognoses, suggesting these as potential drug targets. Our findings indicate that malignant stem-like/EMT cancer cell state might be regulated through reciprocal cancer cell-macrophage intercellular communication and metabolic reprogramming in the heterogeneous TME of gastric cancer at the single-cell level.

## 1. Background

Metabolic reprogramming is a hallmark of cancer [1]; however, given intratumoral heterogeneity, the exact metabolic characteristics of diverse cell types coexisting in the tumor microenvironment (TME) remain elusive. To understand the high-resolution mechanistic influences of cell-type specific metabolic pathway alterations on tumor cells, it is imperative to investigate metabolic activity at the single-cell level rather than in bulk samples [2]. Metabolic reprogramming via nutrient levels, oxygen tension, and the presence of signaling factors allows cells to adapt to and function in a specific TME and communicate with neighboring cells [3]. The tumor metabolic process is complex, and tumor cells select a metabolic energy pathway suitable for their environment to survive. Specifically, tumor cells adopt different metabolic pathways to maintain their oncogenic potential in the TME in which metabolic heterogeneity occurs depending on diverse stimuli by stromal cells. The tumor stroma mainly consists of fibroblasts, endothelial cells, several types of immune cells, and an extracellular matrix (ECM). Interaction among the cancer cells, stromal cells, and ECM in the TME leads to tumor progression; moreover, the tumor stroma continuously changes during cancer progression, promoting cancer cell survival, invasion, and metastasis [4]. Epithelial-mesenchymal transition (EMT) and stem-like cancer cells, in particular, rely on highly energetic metabolic pathways to support stress-resistance and malignant phenotypes such as anti-cancer therapy-induced genotoxic stress, migration, invasion, and metastasis. Inevitably, reactive oxidative species (ROS) generated in large quantities can threaten cell fitness. Therefore, EMT/stem-like cancer cells should devise biological mechanisms to overcome ROS stress.

In this study, we focused on distinct metabolic pathways activated in different cell types in tumor tissue to assess the metabolic activity that helps to acquire the stem-like state. We analyzed metabolic gene expression in 21,085 cells with a stem-like state and elucidated the metabolic reprogramming at the single-cell level. Further, we evaluated specific cell–cell interactions driving metabolic changes associated with cancer cell stemness. By doing so, we identified stem-like gastric cancer-specific drug targets. 

## 2. Methods

### 2.1. Data Preprocessing

We downloaded gastric cancer single-cell data [5] from the website of the Ji Research Group (https://dna-discovery.stanford.edu/research/datasets/, accessed on 29 March 2021). The raw output data were processed with the Seurat package (Seurat V3 [6]) using R software (R version 4.0.5). For downstream analysis, we filtered cells that meet the following criteria: (1) cells that have unique feature counts < 200 or >2500 (in the outlier range, because the latter might be indicative of potential doublets) and (2) had >5% of mitochondrial counts (Seurat default). Thus, we applied more stringent filter criteria than in the previous study, which is why there are fewer cells than in the previous reports. A total of 12,422 filtered cells were included for further bioinformatics analysis. We also used microarray data derived from bulk samples of 497 gastric cancer patients from the Yonsei cohort in Gene Express Omnibus (registration number: GSE84437, accessed on 15 May 2021). For patient survival validation, The Cancer Genome Atlas Stomach Adenocarcinoma (TCGA-STAD) was used in GEPIA2 [7].

### 2.2. Metabolic Pathway Activity and Cancer Hallmarks

To calculate metabolic pathway activity, we analyzed 84 Kyoto Encyclopedia of Genes and Genomes (KEGG) metabolic pathways. MAGIC [8] was used for imputing missing single-cell data. The information regarding the pathway activities for each cell was obtained using single-sample gene set enrichment with the R package GSVA [9]. To assess the significance of the scores, we estimated *p*-values by generating the background distribution using the permutation of the expression profiles (10,000,000 times). ROS signatures and 50 cancer hallmark gene sets were obtained from MSigDB (http://software.broadinstitute.org/gsea/msigdb, accessed on 2 May 2021).

### 2.3. Meta-Analysis and Deconvolution Analysis

We used RaceID and StemID from the R package to identify single-cell stem cells [10].

xCell was used for cell type classification from microarray Y497 bulk samples [11].

## 3. Results

### 3.1. Transcriptional Landscape of Cell Type-Specific Metabolic Heterogeneity in Gastric Cancer

**Figure 1:**
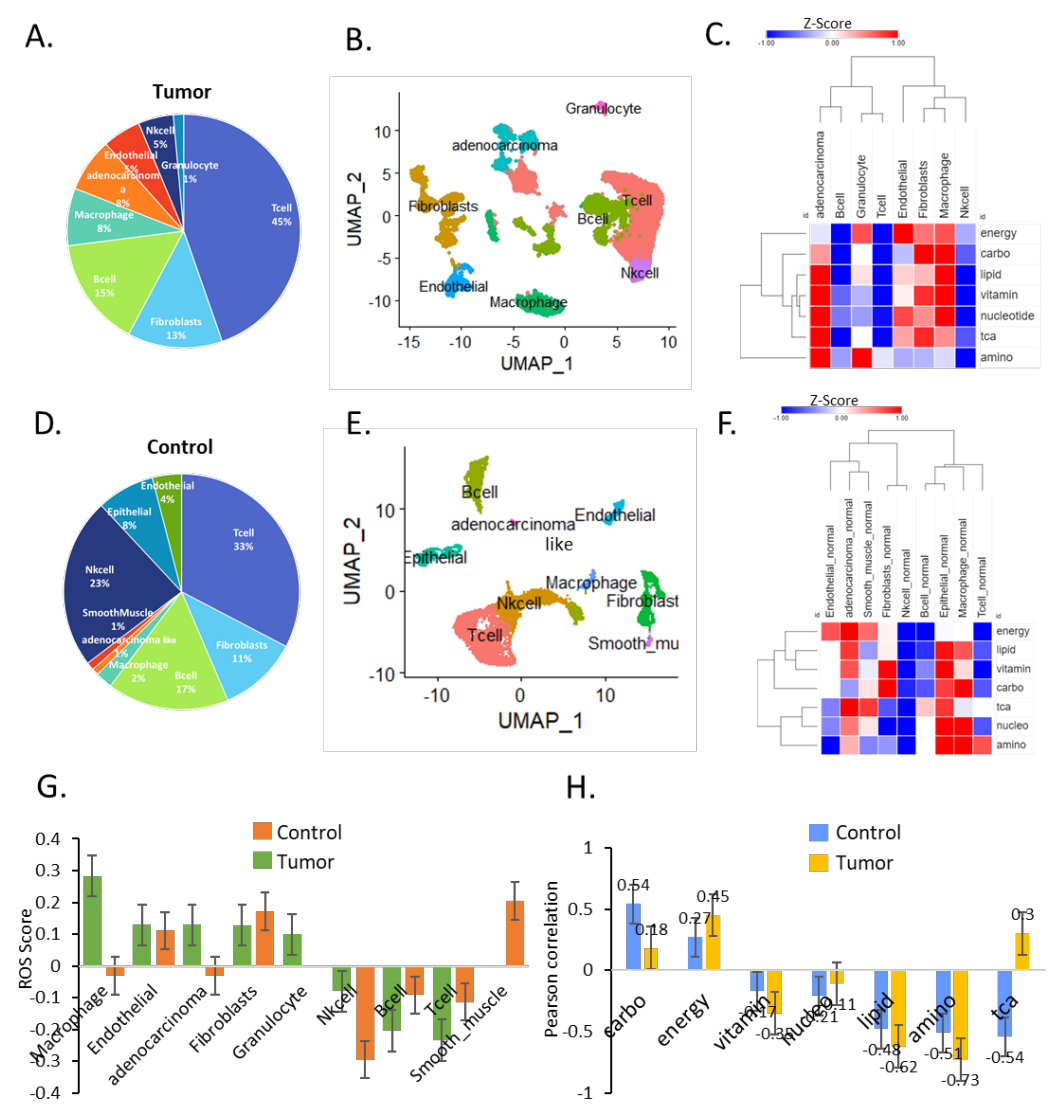
**Single**-**cell analysis of metabolic reprogramming.** (**A**) Pie chart for tumor cell types: T cells 45%, fibroblasts 13%, B cells 15%, macrophages 8%, adenocarcinoma cells 8%, endothelial cells 5%, NK cells 5%, and granulocytes 1%. (**B**) Uniform Manifold Approximation and Projection (UMAP) plot of the eight main cell types identified in gastric lesions. (**C**) Heatmap of seven metabolic reprogramming signatures for the eight main cell types in tumors. (**D**) Pie chart for cell types of the control: T cells 33%, fibroblasts 11%, B cells 17%, macrophages 2%, adenocarcinoma cells 1%, smooth muscle cells 1%, endothelial cells 4%, NK cells 23%, and epithelial cells 8%. (**E**) UMAP plot of the nine main cell types identified in adjacent normal gastric lesions (paired sample). (**F**) Heatmap of seven metabolic reprogramming signatures for the nine main cell types in the normal control. (**G**) ROS score of cell types in tumors and the control. (**H**) Pearson correlation between ROS and the seven metabolic reprogramming signatures in tumors and control.

We analyzed pan-cancer metabolic reprogramming in bulk samples obtained from The Cancer Genome Atlas (TCGA) reported in previous studies [2]. However, considering intratumoral heterogeneity, there are limitations to bulk analysis, which only provides average gene expression data of diverse cell types within tumor tissue. To overcome these limitations, a single-cell RNA sequencing (scRNA-seq) dataset of gastric cancer [5] was used to assess the cell type-specific metabolic reprogramming in 21,085 cells at the single-cell level. We used seven gastric cancer patients and one gastric intestinal metaplasia (GIM) as a tumor group (12,422 cells) based on the previous results of transcriptional similarity of metaplastic cells with gastric cancer type 2 cancer cells and paired normal cell controls (8663 cells). Since only 2000–4000 genes are expressed specifically in each single cell, MAGIC was used for imputing missing data [8]. The unbiased clustering of the cells identified eight main clusters for the tumors (Figure 1A, Table S1) and nine main clusters for the controls (Figure 1D). Next, we performed Uniform Manifold Approximation and Projection (UMAP) analysis to identify subclusters from tumors and adjacent normal controls using gene expression profiling and cluster marker genes (Figure 1B,E and Table S1). Interestingly, there was a small number of adenocarcinoma-like cells in the normal control, which were distinct from epithelial cells (n = 82) (Figure 1D,E). Among the tumor samples, adenocarcinoma cells, endothelial cells, fibroblasts, and macrophages were mostly enriched in seven metabolic signatures (Figure 1C). We found that adenocarcinoma cells had low integrated energy metabolic activity (Figure 1C). On the contrary, adenocarcinoma-like cells in normal tissue exhibited high energy metabolic pathways and low carbohydrate metabolic activity (Figure 1F). The level of glycolysis differed significantly (false discovery rate [FDR] = 0.01) between tumor and normal control samples, suggesting that metabolic reprogramming is highly enriched in specific cell types, such as adenocarcinoma cells, endothelial cells, fibroblasts, and macrophages. 

In addition, we performed ROS analysis on different cell types present in the TME. Low to moderate ROS levels are essential for cell survival and proliferation, but excessive ROS has detrimental effects on cell fitness. In tumor samples, macrophages and adenocarcinoma cells had significantly higher ROS levels than the controls (Figure 1G). Furthermore, the ROS levels in tumor sample subcluster cells positively correlated with energy metabolism and the TCA pathway (Figure 1H). 

Given that energy and the TCA pathway are integral to mitochondrial bioenergetics, these results show that a distinct ROS pathway is associated with the mitochondrial energy metabolism of adenocarcinoma cells and macrophages in the TME.

### 3.2. Heterogeneous Immune and Macrophage Metabolic Landscapes in the TME

Tumor and immune cells compete for nutrients [12]. Innate immune cells show comparatively lesser metabolic reprogramming than adenocarcinoma cells. In this study, we determined the metabolic signature used by the immune cells for mounting the immune response, since tumors reportedly inhibit the function of tumor-infiltrating T cells via competitive glucose uptake [13]. 

**Figure 2:**
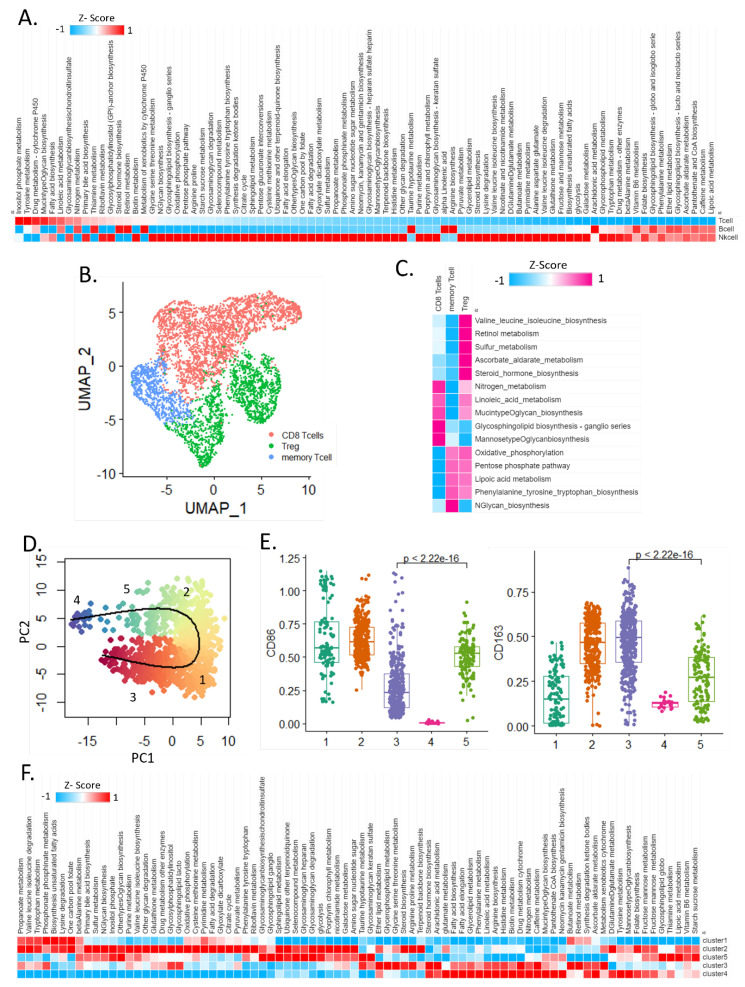
**Immune and macrophage cell heterogeneous metabolic landscapes in the TME.** (**A**) Heat map of the activity of 84 metabolic pathways in immune cells (T cells, B cells, NK cells). (**B**) Uniform Manifold Approximation and Projection (UMAP) plot of the three sub-clusters in T cell. (**C**) Heat map of highly enriched metabolic pathways in the T cell subtypes. (**D**) Macrophage lineage tree by using slingshot. (**E**) M1 and M2 marker gene for five clusters. (**F**) Heat map of highly enriched metabolic pathways in the macrophage cluster.

Our results showed that, overall, NK cells undergo a relatively more complex metabolic reprogramming compared to T cells and B cells. Among 84 metabolic pathways, B cells showed relatively higher activities of alpha-linolenic acid metabolism, arginine biosynthesis, and taurine and hypotaurine metabolism, whereas T cells showed relatively higher activities of tyrosine metabolism, linoleic acid metabolism, fatty acid biosynthesis, and inositol phosphate metabolism, while NK cells showed relatively complex metabolic reprogramming that included glycolysis and oxidative phosphorylation (OXPHOS) (Figure 2A).

Next, we performed UMAP analysis, which facilitated the classification of T cells into three subtypes and revealed the marker genes for each cluster (Figure 2B). Specifically, the OXPHOS, pentose phosphate pathway, ascorbate and aldarate metabolism, valine, leucine and isoleucine biosynthesis, sulfur metabolism, and lipoic acid metabolism were relatively upregulated in Treg cells compared to those in CD8+ T cells. The CD8+ T cells used glycosphingolipid biosynthesis—ganglio and linoleic acid metabolism, nitrogen metabolism, and mannose type O-glycan biosynthesis—for executing their functions (Figure 2C).

The metabolic reprogramming of M1 and M2 macrophages occurs differently depending on the macrophage polarization states [14]. M1 macrophages [15,16] use glycolysis and pentose phosphate pathways, whereas M2 macrophages [17,18] mainly rely on OXPHOS and fatty acid oxidation (FAO) to supply energy.

We analyzed the macrophage lineage tree using slingshot [19] and divided it into five clusters and investigated metabolic reprogramming in macrophages by their metabolic activity (Figure 2D,E).

Cluster analysis identified that in cluster 4, the M1 marker gene CD86 was relatively lower than that of the other clusters, and the expression of the M2 marker gene CD163 was high. Clusters 1, 2, 3, and 5 had both M1 and M2 marker genes expression at the same time (Figure 2E). In cluster 4, 27 metabolic pathways including glutamate metabolism and fatty acid biosynthesis were upregulated. In contrast, glycosaminoglycan (GAG) degradation, GAG biosynthesis-heparan sulfate (HS) [20], sphingolipid metabolism, glycosphingolipid biosynthesis-globo, and isoglobo pathways were relatively upregulated in cluster 2 and cluster 5 (Figure 2F).

Together, these results suggest that different types and subsets of immune cells and macrophages undergo distinct metabolic adaptations for their existence and functions within the heterogeneous TME.

### 3.3. Comparison of Intratumoral Metabolic Heterogeneity between Adenocarcinoma Cells and Bulk Samples

**Figure 3:**
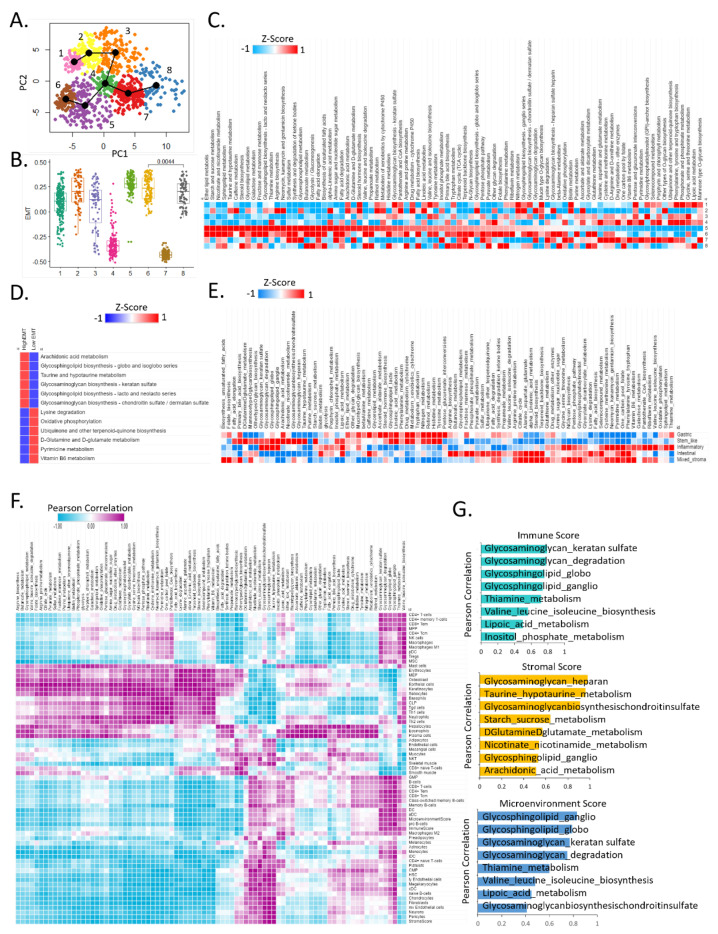
**Comparison of intratumoral metabolic heterogeneity between adenocarcinoma cells and bulk samples.** (**A**) Adenocarcinoma lineage plot adenocarcinoma single cells from the gastric tumor dataset. (**B**) Boxplot of EMT score for eight adenocarcinoma clusters. (**C**) Heat map of 84 metabolic pathways for clustering adenocarcinoma cells. (**D**) Heat map of top six enriched metabolic pathways between high EMT and low EMT. (**E**) Heat map of 84 metabolic pathways for molecular subtype of gastric cancer (497 Yonsei Hospital patients). (**F**) Heat map of Pearson correlation with 84 metabolic pathways and 64 cell types from bulk samples (purple: positive correlation, blue: negative correlation). (**G**) Bar graph of the immune score, stromal score, and microenvironment score using correlation coefficient values.

Cancer cells and immune cells compete for nutrients in the TME. At single-cell resolution, adenocarcinoma cells exhibit complex metabolic reprogramming compared to that of other immune cells [21]. We clustered 924 adenocarcinoma cells in tumor samples based on the expression of metabolic genes. Slingshot [19] analysis revealed intratumoral metabolic heterogeneity in eight clusters (Figure 3A). Specifically, among tumor samples, the cluster 5 cells overexpressed an EMT score; cluster 4 cells did not overexpress an EMT score (Figure 3B). Each cluster exhibited specific metabolic reprogramming. 

Cluster 5 with high EMT was enriched for ether lipid metabolism and starch and sucrose metabolism, and cluster 5 and cluster 6 showed similar metabolic reprogramming patterns, but cluster 4 showed the opposite pattern (Figure 3C). Cluster 4 with low EMT was highly enriched in the glycan degradation pathway (FDR < 0.01) (Figure 3C). Among 924 adenocarcinoma cells, high-EMT and low-EMT cells were classified, and the metabolic activation pathways were analyzed, showing a distinct metabolic reprogramming pattern. At high EMT, arachidonic acid metabolism, glycosphingolipid biosynthesis-globo and isoglobo series, taurine and hypotaurine metabolism, glucosaminoglycan biosynthesis-keratan sulfate, glycosphingolipid biosynthesis-lacto and neolacto series, glycosaminoglycan biosynthesis-chondroitin sulfate, and dermatan sulfate were upregulated. On the other hand, lysine degradation and oxidative phosphorylation were upregulated at low EMT (*p* < 0.001) (Figure 3D).

Interestingly, purine metabolism was upregulated in cluster 6 adenocarcinoma cells at the single-cell level but was not upregulated in any molecular type in the bulk samples of the Yonsei Hospital cohort (Figure 3E). Next, we analyzed bulk samples of gastric cancer. All the five molecular subtypes from the Yonsei Hospital cohort that had distinct prognoses [22] showed specific metabolic reprogramming in bulk analysis. 

Notably, the stem-like subtype showed distinctive metabolic reprogramming patterns among other subtypes with upregulated GAG, nicotinate and nicotinamide metabolism, and starch sucrose metabolism, while it downregulated glutathione metabolism (Figure 3E). However, at the single-cell level, there was a different activity of the TCA cycle and oxidative phosphorylation among four EMT high clusters, implying cancer cell subset-specific heterogeneous metabolic reprogramming in tumor tissues (Figure 3C). The activity related to GAG and glycosphingolipid was high in the stem-like subtype in bulk analysis. Stem-like subtype tumors share biological similarities with the EMT phenotype [23]. In line with the bulk analysis results, GAG and glycosphingolipid showed high activity in tumor cells with high EMT activity at the single-cell level. Further, among various metabolic pathways, only taurine and hypotaurine metabolism is highly upregulated both in bulk stem-like subtype tumors as well as in tumor cells with high EMT clusters at the single-cell level. These results suggest that cancer cell-intrinsic alterations in taurine and hypotaurine metabolism are specific to stem-like and EMT tumors.

Next, we successfully performed immune profiling in adenocarcinoma cells at the single-cell level in gastric cancer and in bulk samples (Figure 3F). We analyzed the correlation between cell types and 84 metabolic pathways in Y497 bulk samples.

According to KEGG, CD4+ T cells, CD4+ memory T cells, CD8+ effector memory T cells (Tem), MPP, CD4+ central memory T cells (Tcm), NK cells, macrophages, M1 macrophages, dendritic cells (DCs), T regulatory cells, and mesenchymal stem cells (MSCs) positively correlated with GAG biosynthesis—keratan sulfate, GAG degradation, glycosphingolipid biosynthesis—globo and isoglobo, and glycosphingolipid biosynthesis—ganglio, thiamine metabolism, valine, leucine, and isoleucine biosynthesis (Figure 3F). The immune score showed a high correlation with GAG-keratan sulfate (R = 0.74), while the stromal score showed a high correlation with GAG biosynthesis-HS (R = 0.98). The microenvironment score showed a high correlation with glycosphingolipid biosynthesis-ganglio (R = 0.83) (Figure 3G). Collectively, these results demonstrate that GAG [24] may affect immune and stromal cell function and tumor progression according to tumor environmental conditions.

### 3.4. Identification of High Stemness Cells and Metabolic Reprogramming

**Figure 4:**
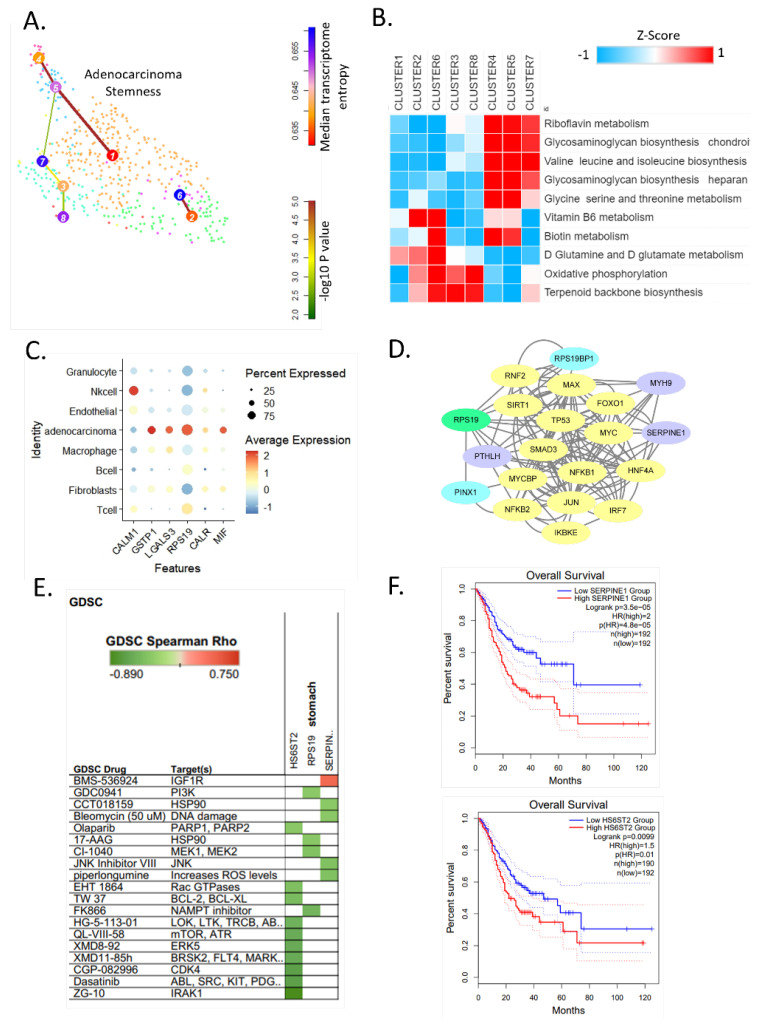
Metabolic heterogeneity and stem cell identification and cell–cell interaction correlates with therapeutic sensitivity. (**A**) RaceID3 and StemID2 analysis of 924 adenocarcinoma cells from the original clusters. The link color indicates the link *p*-value, and the vertex color represents transcriptome entropy. The link *p*-value and transcriptome entropies were derived by StemID2. The thickness and color of a link indicate the transition probability between the connected clusters. (**B**) Heat map of enriched metabolic pathway for adenocarcinoma clusters. (**C**) Dot plot of best upstream ligands for eight cell types. (**D**) Signaling path for the RPS19 ligand and the target gene link. (**E**) Genomics of Drug Sensitivity in Cancer (GDSC) drug and target genes for gastric cancer. Green (negative correlation) indicates high drug sensitivity (**F**) Kaplan–Meier plot showing recurrence-free survival rates for high and low *SERPINE1* expression in gastric cancer of TCGA cohort (up) and *HS6ST2* (down).

Next, we used VarID and StemID to identify cells likely to become stem cells [10] based on their metabolic pathways. We analyzed differential gene expression in 924 adenocarcinoma cells of tumor samples to identify cells with high metabolic reprogramming and stemness (Figure 4A). VarID quantifies the gene expression variability, whereas StemID permits the inference of a lineage tree based on clusters (i.e., cell types) identified by RaceID. Next, we analyzed the stemness of the clusters. The entropy from cluster 7 to cluster 6 was the highest, and the stem cell transition from cluster 7 to cluster 5 was also predicted (Figure 4A,B). Clusters 6 and 7 had the highest stemness cells. Clusters 4, 5, and 7’s cells are upregulated in riboflavin metabolism, glycosaminoglycan biosynthesis—chondroitin sulfate/dermatan sulfate, valine leucine and isoleucine biosynthesis, glycosaminoglycan biosynthesis heparan, glycine serine and threonine metabolism (Figure 4B). We compared metabolic reprogramming of eight clusters of cells with high stemness and observed that the GAG biosynthesis was highly expressed in cluster 7 (Figure 4B). 

Next, we attempted to identify novel therapeutic targets related to the highest stemness in 924 adenocarcinoma cells. Among eight clusters, clusters 6 and 7 showed the highest stemness score. The activity of six ligands expressed in multiple cell types indicated that RPS19 was overexpressed in adenocarcinoma (Figure 4C). Since the metabolic reprogramming of macrophages among other cell types in the TME was most significantly altered along with adenocarcinoma cells (Figure 1C), we evaluated the signaling interaction between macrophages and adenocarcinoma cells. To this end, we used NicheNet to predict ligand-target links between interacting cells [25]. We investigated the key transcription factors that regulate the expression of target genes and that are most closely downstream of the ligand (based on the weights of the edges in the integrated ligand signaling and gene regulatory networks). Then, the shortest paths between these transcription factors and the ligands of interest were determined, and the genes that were part of these paths were considered important signaling mediators.

RPS19 and CALM1 were predicted as prioritized active ligands for macrophages. The genes in selected metabolic pathways in adenocarcinoma cells potentially regulated by these ligands were predicted as follows: *ETFB, HS6ST2,* and *NDST1.*

When the ligand-target links between adenocarcinoma and macrophages were investigated, we identified *CALM1*, *RPS19*, *GSTP1*, and *LGALS3* as prioritized adenocarcinoma expressed ligands. We used *RPS19* as a ligand and *MYH9*, *PTHLH*, and *SERPINE1* as targets with 14 transcriptional regulators (Figure 4D). Then, we identified signaling pathways between an adenocarcinoma ligand and some of its top predicted macrophage target genes. Intriguingly, the common ligand of *RPS19* potentially regulates *SERPINE1* in macrophages (Figure 4D). Indeed, SERPINE1 is expressed in tumor-associated macrophages in esophageal squamous cell carcinoma and it promotes cancer cell invasion and macrophage migration [26]. 

We performed a drug-target prediction analysis using the Genomics of Drug Sensitivity in Cancer (GDSC) database [27] and identified candidate drugs (ZG10, Dasatinib, CGP-082996) for *HS6ST2*, *RPS19*, and *SERPINE1* in gastric cancer (Figure 4E). We further examined its clinical relevance using another dataset (TCGA STAD), which revealed poor outcomes (*p* = 3.5 × 10^−5^) when *SERPINE1* is overexpressed [28]. *HS6ST2* (*p* = 0.0099) overexpression was also related to poor outcomes (Figure 4F). Piperlongumine [29] inhibits cancer growth by inducing the accumulation of intracellular ROS, decreasing glutathione, damaging chromosomal DNA, and modulating key regulatory proteins, including PI3K, AKT, mTOR, NF-κB, STATs, and cyclin D1. Piperlongumine also induces apoptosis in gastric cancer cells [30,31].

## 4. Discussion

Cancer and stromal cells depend on metabolic reprogramming for their proper growth and functioning [32]. In the highly competitive metabolic TME, tumor cells not only compete with stromal cells for nutrition but also co-opt stromal cells via intercellular communications. To the best of our knowledge, in gastric cancer, metabolic reprogramming and cell–cell interaction have not been reported in diverse cell types at the single-cell level. Our results demonstrated that distinct metabolic pathways are differentially activated in specific cell types. High stemness and EMT score cells showed elevated GAG metabolic signatures, which were associated with an unfavorable prognosis. 

Cell–cell interaction analysis identified that cancer cells and macrophages in the highest stemness cluster communicate through the common ligand RPS19. The ligand-target regulated transcriptional reprogramming resulted in *HS6ST2* expression in adenocarcinoma cells, while it may lead to the expression of *SERPINE1* in macrophages [33]. In line with this, adenocarcinoma-like cells in normal tissue use various energy sources in the TME, primarily glycan metabolism [34]. Moreover, glycan metabolism was upregulated in the poor prognostic stem-like cell subtype in the bulk analysis of 497 gastric cancer patients. Collectively, these single cell and bulk analyses results suggest that reciprocal intercellular communications between macrophages and cancer cells promoting stem-like GC progression are associated with GAG metabolism in the TME. HS, one of the major GAGs expressed at the cell membrane, is sulfated at 2-O- of uronic acid residues, and 3-O- and 6-O of GlcN units. M1 and M2 activation drastically modified the profiles of the expression of numerous HS sulfotransferases, which was accompanied by the expression of GAGs with distinct structural features. The ability of macrophage GAG species to present FGF-2 might promote tumor cell proliferation. Indeed, M2 macrophages could be efficient promoters of FGF-2-dependent proliferation of adjacent cancer cells. In M2 macrophages, the expression of *HS6ST2* was increased by a factor of two, while HS3ST3B was barely detected. Indeed, the removal of cell surface HS by heparinases potently reduced FGF-2-induced cell proliferation, suggesting the importance of HS6ST2 in augmenting M2 macrophages’ induced cancer cell promotion [35].

In addition, GAG is partially degraded in ECM and endocytosed into cells, wherein being associated with lysosome, it inhibits lysosomal cathepsin activity, thereby reducing ROS, maintaining mitochondrial membrane potential, and inhibiting cyt c release [36].

Our findings suggest that high stemness cancer cells co-opt macrophages to promote cell survival and malignant progression via cell–cell interaction-mediated ROS regulation in highly energetic cancer metabolic reprogramming. Metabolic pathways with a high correlation with immune scores are shown in top-ranking order (Figure 3G). In particular, the stromal score and the glycosaminoglycan heparan were highly positively correlated.

Understanding metabolic reprogramming-related markers and pathways may provide insights into cancer metabolism-associated features specific for each cell type and their potential as novel drug targets. Gastric cancer patients with increased *SERPINE1* and *HS6ST2* expression had unfavorable prognoses (*p* = 0.000035 and *p* = 0.009, respectively) [37], suggesting these as potential drug targets. Our integrated analyses highlight novel findings that malignant stem-like/EMT cancer cell state might be regulated through reciprocal cancer cell-macrophage intercellular communication and metabolic reprogramming in the heterogeneous TME of gastric cancer at the single-cell level [38].

Collectively, refractory cancer cells, stem-like EMT cancer cells, devise highly intricate hetero-molecular signaling modules through co-opting macrophages to overcome oxidative stress and maintain stem cell fitness in metabolically competitive and immunosurveillant TME.

Although we attempted to delineate the metabolic characteristics of each cell type existing in the TME, a vast number of metabolic pathways were differently enriched in immune cell phenotypes. This impeded a clear understanding regarding the biological implication of distinct metabolic activities occurring in different cell types. Nevertheless, we believe that our results will be useful for future investigation and facilitate a detailed understanding of metabolic reprogramming and cell–cell interaction in the heterogeneous TME.

## 5. Conclusions

This study is the first attempt to profile the metabolic activity of different cell types at the single-cell level in the TME of gastric cancer and it successfully demonstrated metabolic reprogramming and intercellular communications between cancer cells and macrophages that contribute to the stemness and EMT state of gastric cancer at the single-cell level. Furthermore, our study outcomes present potential therapeutic targets and drugs based on intercellular interactions associated with specific metabolic pathways related to unfavorable cancer prognosis. 

## Data Availability

Not applicable.

## Supplementary Materials

The following supporting information can be downloaded at: https://www.mdpi.com/article/10.3390/cells11152373/s1, Table S1: Marker gene for clusters.

## Abbreviations

TME, tumor microenvironment; ECM, extracellular matrix; EMT, Epithelial-mesenchymal transition; ROS, reactive oxidative species; TCGA-STAD, The Cancer Genome Atlas Stomach Adenocarcinoma; KEGG, Kyoto Encyclopedia of Genes and Genomes; TCGA, The Cancer Genome Atlas; scRNA-seq, RNA sequencing; GIM, gastric intestinal metaplasia; UMAP, Uniform Manifold Approximation and Projection; FDR, false discovery rate; OXPHOS, oxidative phosphorylation; GAG, glycosaminoglycan; HS, heparan sulfate; Tcm, memory T cells; DC, dendritic cells; MSCs, mesenchymal stem cells; GDSC, Genomics of Drug Sensitivity in Cancer.
