# Supplementary material for: Single Cell Analysis Reveals Reciprocal Tumor-Macrophage Intercellular Communications Related with Metabolic Reprogramming in Stem-like Gastric Cancer
Source: Cells. 2022 Aug 2;11(15):2373. doi: 10.3390/cells11152373 (PMC9368184; doi:10.3390/cells11152373)
Supplement: Supplementary file 1 [file cells-11-02373-s001.zip › Supplementary Table S1.pdf]

Table S1. Marker gene for clusters

Gene	p_val	avg_log2FC	pct.1	pct.2	p_val_adj	cluster
IL7R	0	2.495835621	0.487	0.106	0	Tcell
CD3D	0	2.119857484	0.559	0.079	0	Tcell
CD2	0	2.028339926	0.481	0.072	0	Tcell
DUSP4	0	1.893535285	0.418	0.12	0	Tcell
TRBC2	0	1.69124078	0.375	0.072	0	Tcell
PTPRC	0	1.660171838	0.667	0.235	0	Tcell
FYN	0	1.656467152	0.469	0.144	0	Tcell
TRAC	0	1.649241862	0.328	0.062	0	Tcell
CNOT6L	0	1.601026824	0.474	0.17	0	Tcell
CXCR4	0	1.595785167	0.72	0.315	0	Tcell
CD7	0	1.530195326	0.413	0.116	0	Tcell
FYB1	0	1.524784589	0.416	0.124	0	Tcell
CD52	0	1.477765479	0.531	0.185	0	Tcell
CD3E	0	1.447887921	0.312	0.046	0	Tcell
CD3G	0	1.446577369	0.343	0.058	0	Tcell
BTG1	0	1.395650944	0.926	0.699	0	Tcell
RGS1	0	1.370835731	0.624	0.285	0	Tcell
CREM	0	1.195408174	0.643	0.337	0	Tcell
TMSB4X	0	0.658358857	0.993	0.975	0	Tcell
B2M	0	0.626217816	0.994	0.976	0	Tcell
RPL28	0	0.529605688	0.988	0.965	0	Tcell
ZFP36L2	1.50E-302	1.343709995	0.71	0.529	3.20E-298	Tcell
RNF19A	2.11E-288	1.587201838	0.434	0.174	4.49E-284	Tcell
SYTL3	6.37E-287	1.332877596	0.418	0.136	1.36E-282	Tcell
FAM129A	1.15E-284	1.605161488	0.334	0.092	2.46E-280	Tcell
SRGN	4.57E-284	0.763508943	0.783	0.489	9.75E-280	Tcell
CCL5	1.22E-283	1.882401657	0.438	0.163	2.61E-279	Tcell
RPS15A	1.68E-282	0.47332536	0.983	0.969	3.59E-278	Tcell
SARAF	9.39E-269	1.354566782	0.591	0.397	2.00E-264	Tcell
IL32	2.92E-262	1.407128589	0.567	0.338	6.22E-258	Tcell
TRBC1	6.78E-261	1.542433216	0.331	0.09	1.45E-256	Tcell
LTB	2.31E-259	1.497974006	0.283	0.058	4.93E-255	Tcell
ACAP1	1.77E-256	1.299104824	0.316	0.083	3.78E-252	Tcell
RPS3	2.36E-254	0.518221039	0.965	0.924	5.04E-250	Tcell
LEPROTL1	4.56E-251	1.466790892	0.411	0.172	9.73E-247	Tcell
SLA	9.05E-250	1.34295341	0.386	0.137	1.93E-245	Tcell
STK4	9.71E-248	1.249021907	0.489	0.241	2.07E-243	Tcell
SPOCK2	3.84E-240	1.178551476	0.264	0.055	8.19E-236	Tcell
BATF	1.21E-234	1.60987419	0.284	0.072	2.58E-230	Tcell
RPL30	7.06E-234	0.534899145	0.939	0.887	1.51E-229	Tcell
MALAT1	3.88E-224	0.482244434	1	0.989	8.28E-220	Tcell

RPLP1	1.54E-214	0.445044366	0.992	0.987	3.28E-210	Tcell
ETS1	2.68E-214	1.248189753	0.419	0.193	5.72E-210	Tcell
RPLP2	1.24E-209	0.409120995	0.985	0.968	2.64E-205	Tcell
CLEC2D	6.17E-204	1.16185132	0.262	0.068	1.32E-199	Tcell
CRYBG1	3.19E-191	1.254758295	0.264	0.077	6.80E-187	Tcell
LINC00513	4.70E-191	1.29322674	0.342	0.139	1.00E-186	Tcell
RPL13	6.17E-191	0.423041307	0.987	0.976	1.32E-186	Tcell
SMCHD1	2.50E-190	1.219454476	0.427	0.221	5.34E-186	Tcell
TNFAIP3	1.53E-188	1.002436548	0.56	0.352	3.26E-184	Tcell
STK17B	6.12E-187	1.114467669	0.376	0.166	1.31E-182	Tcell
IKZF1	4.45E-185	1.0984773	0.296	0.099	9.49E-181	Tcell
RPL13A	2.21E-180	0.372909068	0.989	0.979	4.71E-176	Tcell
RPS29	2.43E-178	0.450034	0.985	0.971	5.18E-174	Tcell
RGCC	5.14E-171	1.11524144	0.477	0.278	1.10E-166	Tcell
AC058791.	6.74E-171	1.174406506	0.386	0.186	1.44E-166	Tcell
RPL23A	4.99E-170	0.403930674	0.949	0.929	1.06E-165	Tcell
RUNX3	4.69E-168	1.086184734	0.347	0.147	1.00E-163	Tcell
SRSF7	2.92E-166	1.160104997	0.523	0.358	6.24E-162	Tcell
RORA	4.44E-164	1.298099109	0.328	0.145	9.47E-160	Tcell
PPP2R5C	1.77E-160	1.032361558	0.408	0.216	3.78E-156	Tcell
SAMSN1	9.40E-159	1.011954427	0.358	0.163	2.01E-154	Tcell
TSC22D3	1.10E-155	0.831838832	0.678	0.551	2.35E-151	Tcell
KLRB1	5.58E-155	1.419864117	0.277	0.099	1.19E-150	Tcell
AC016831.	2.37E-154	1.144427637	0.376	0.194	5.06E-150	Tcell
ARHGDIB	1.14E-149	0.93706307	0.451	0.264	2.42E-145	Tcell
RPS2	6.30E-149	0.328273858	0.976	0.948	1.34E-144	Tcell
CYTIP	9.04E-149	0.989114259	0.325	0.146	1.93E-144	Tcell
SYNE2	3.14E-147	1.094735682	0.342	0.165	6.70E-143	Tcell
RPS19	9.93E-145	0.360114264	0.982	0.968	2.12E-140	Tcell
HCST	3.52E-144	0.916315661	0.359	0.168	7.52E-140	Tcell
RPS27	3.96E-143	0.333737229	0.994	0.989	8.45E-139	Tcell
RPL27A	4.77E-143	0.345299234	0.972	0.948	1.02E-138	Tcell
RPS14	1.01E-137	0.350448582	0.969	0.946	2.16E-133	Tcell
CORO1A	2.64E-137	0.892455455	0.324	0.146	5.64E-133	Tcell
RPS26	3.56E-137	0.769644098	0.776	0.731	7.59E-133	Tcell
AAK1	6.09E-134	1.080650199	0.406	0.249	1.30E-129	Tcell
EML4	2.03E-133	1.129400139	0.318	0.159	4.32E-129	Tcell
EEF1A1	2.01E-132	0.397915354	0.97	0.937	4.30E-128	Tcell
DUSP2	1.15E-130	1.005423452	0.356	0.182	2.46E-126	Tcell
RPSA	3.40E-129	0.482498515	0.825	0.781	7.25E-125	Tcell
NR3C1	4.52E-127	1.198015369	0.332	0.177	9.65E-123	Tcell
RPS7	2.87E-126	0.391498663	0.904	0.855	6.12E-122	Tcell
RPS4X	5.50E-125	0.393131439	0.94	0.906	1.17E-120	Tcell

HLA-B	1.19E-123	0.381190444	0.893	0.835	2.54E-119	Tcell
EVL	1.22E-123	1.066856611	0.282	0.129	2.61E-119	Tcell
RPS27A	1.24E-121	0.333683225	0.961	0.95	2.64E-117	Tcell
RHOH	6.33E-120	0.870111169	0.258	0.106	1.35E-115	Tcell
EIF1	6.81E-120	0.417033714	0.911	0.867	1.45E-115	Tcell
HLA-A	9.20E-114	0.474889187	0.83	0.771	1.96E-109	Tcell
DDX24	4.78E-109	0.819272898	0.545	0.438	1.02E-104	Tcell
AKNA	6.38E-107	1.003143865	0.271	0.125	1.36E-102	Tcell
PDCD4	9.59E-104	0.967610127	0.296	0.155	2.05E-99	Tcell
RPS12	1.05E-103	0.298728746	0.982	0.968	2.24E-99	Tcell
ELF1	3.54E-102	0.90198608	0.425	0.291	7.55E-98	Tcell
RPS25	5.12E-101	0.302067156	0.933	0.927	1.09E-96	Tcell
FNBP1	8.11E-100	0.863526443	0.369	0.227	1.73E-95	Tcell
BIRC3	7.29E-99	0.822949441	0.365	0.214	1.56E-94	Tcell
CDC42SE2	2.03E-97	0.873105026	0.308	0.171	4.33E-93	Tcell
RPL10	3.39E-97	0.278836555	0.991	0.984	7.23E-93	Tcell
ARID4B	1.85E-95	0.977438023	0.409	0.29	3.94E-91	Tcell
MT-ND2	2.98E-95	0.3727137	0.949	0.867	6.37E-91	Tcell
PDE4B	2.16E-93	0.874724112	0.303	0.167	4.61E-89	Tcell
IDS	5.32E-93	0.99141487	0.358	0.231	1.14E-88	Tcell
AKAP13	7.19E-93	0.801338838	0.509	0.405	1.53E-88	Tcell
RPL9	1.55E-92	0.366874609	0.897	0.872	3.31E-88	Tcell
TPT1	1.13E-91	0.377455507	0.953	0.914	2.42E-87	Tcell
SMAP2	4.99E-91	0.778918636	0.379	0.24	1.07E-86	Tcell
CD44	9.90E-91	0.715968778	0.491	0.362	2.11E-86	Tcell
ARL4C	4.30E-90	0.79296809	0.347	0.203	9.17E-86	Tcell
RPL14	6.74E-89	0.331229911	0.897	0.871	1.44E-84	Tcell
RPS3A	1.23E-87	0.329384514	0.932	0.902	2.63E-83	Tcell
RPL19	2.08E-85	0.284405438	0.949	0.929	4.43E-81	Tcell
MT-ATP6	1.03E-83	0.324807608	0.966	0.9	2.20E-79	Tcell
RPS16	1.69E-83	0.294123216	0.948	0.932	3.61E-79	Tcell
RPL11	1.91E-82	0.259952822	0.964	0.946	4.09E-78	Tcell
GSPT1	1.22E-81	1.030812021	0.326	0.216	2.61E-77	Tcell
MT-CO1	1.43E-81	0.283791551	0.988	0.953	3.05E-77	Tcell
PABPC1	2.95E-79	0.781638408	0.753	0.739	6.29E-75	Tcell
DDX5	3.01E-78	0.44585726	0.774	0.732	6.42E-74	Tcell
CST7	3.18E-78	0.54363245	0.254	0.121	6.79E-74	Tcell
CD48	9.40E-78	0.674397562	0.261	0.135	2.01E-73	Tcell
NKG7	9.12E-76	0.301333948	0.282	0.138	1.95E-71	Tcell
RNF213	2.04E-74	0.79164936	0.429	0.328	4.37E-70	Tcell
PPP1R2	5.09E-74	0.903378844	0.33	0.22	1.09E-69	Tcell
ZNF331	1.22E-72	0.741311013	0.317	0.192	2.61E-68	Tcell
RPL39	5.42E-72	0.259092903	0.955	0.943	1.16E-67	Tcell

PIK3R1	1.17E-71	1.00752702	0.281	0.171	2.51E-67	Tcell
ISG20	1.06E-69	0.686118474	0.409	0.288	2.27E-65	Tcell
CELF2	1.10E-69	0.851846088	0.287	0.175	2.34E-65	Tcell
FAM177A1	2.17E-69	0.915597356	0.415	0.328	4.64E-65	Tcell
CD53	3.93E-69	0.641812204	0.285	0.163	8.38E-65	Tcell
S100A4	2.61E-67	0.606743144	0.567	0.455	5.57E-63	Tcell
PRPF38B	9.39E-66	0.799517216	0.414	0.326	2.00E-61	Tcell
RPL6	1.57E-65	0.293590592	0.884	0.851	3.36E-61	Tcell
GMFG	6.64E-65	0.684185404	0.308	0.196	1.42E-60	Tcell
RPL3	1.70E-64	0.283400625	0.94	0.921	3.62E-60	Tcell
STK17A	2.19E-62	0.696720421	0.272	0.165	4.67E-58	Tcell
CD37	5.39E-60	0.343592211	0.322	0.198	1.15E-55	Tcell
REL	4.55E-59	0.604324978	0.49	0.39	9.72E-55	Tcell
WNK1	6.59E-59	0.917667607	0.263	0.17	1.41E-54	Tcell
KMT2E	3.75E-58	0.696909269	0.435	0.357	8.00E-54	Tcell
GPBP1	9.27E-55	0.745322799	0.413	0.342	1.98E-50	Tcell
NSD3	4.28E-54	0.805592829	0.301	0.212	9.13E-50	Tcell
YPEL5	5.28E-54	0.778537529	0.349	0.262	1.13E-49	Tcell
FKBP5	1.74E-53	0.92471575	0.277	0.189	3.71E-49	Tcell
CALM1	5.11E-52	0.460089066	0.638	0.606	1.09E-47	Tcell
CEMIP2	2.08E-49	0.757496324	0.254	0.164	4.44E-45	Tcell
ANKRD12	8.11E-49	0.744000895	0.368	0.295	1.73E-44	Tcell
ARID5B	3.34E-47	0.708669015	0.44	0.371	7.12E-43	Tcell
EZR	6.28E-47	0.606818486	0.441	0.366	1.34E-42	Tcell
EEF1D	3.58E-45	0.304723043	0.767	0.781	7.63E-41	Tcell
PRRC2C	6.53E-45	0.626134737	0.481	0.449	1.39E-40	Tcell
FAM107B	9.97E-45	0.689893373	0.333	0.253	2.13E-40	Tcell
RPL36AL	1.27E-43	0.392402425	0.647	0.662	2.70E-39	Tcell
TNFRSF1B	1.45E-42	0.638016796	0.253	0.166	3.10E-38	Tcell
MT-CYB	6.56E-42	0.288879145	0.886	0.826	1.40E-37	Tcell
H3F3B	1.41E-41	0.25431398	0.851	0.83	3.01E-37	Tcell
EPC1	7.16E-37	0.732689727	0.293	0.228	1.53E-32	Tcell
FUS	1.57E-36	0.551536664	0.502	0.469	3.36E-32	Tcell
TSPYL2	6.25E-36	0.66541108	0.291	0.216	1.33E-31	Tcell
CCL4	4.70E-35	0.727606576	0.264	0.181	1.00E-30	Tcell
N4BP2L2	8.36E-35	0.682011978	0.348	0.291	1.79E-30	Tcell
SH3BGRL3	1.57E-32	0.395213679	0.625	0.612	3.35E-28	Tcell
RBPJ	5.76E-31	0.851184665	0.28	0.225	1.23E-26	Tcell
HLA-C	1.24E-30	0.289830555	0.753	0.734	2.65E-26	Tcell
HNRNPA1	2.20E-30	0.357118993	0.619	0.634	4.70E-26	Tcell
NKTR	3.44E-29	0.63456627	0.292	0.235	7.34E-25	Tcell
IFI16	8.05E-29	0.621675442	0.317	0.261	1.72E-24	Tcell
RBM39	7.97E-28	0.392058325	0.559	0.561	1.70E-23	Tcell

SF1	8.27E-28	0.658487359	0.331	0.286	1.76E-23	Tcell
HLA-E	1.24E-26	0.267559578	0.666	0.667	2.65E-22	Tcell
COTL1	6.99E-26	0.536666925	0.34	0.284	1.49E-21	Tcell
RPL4	1.50E-25	0.285932674	0.68	0.709	3.21E-21	Tcell
SRSF5	2.72E-25	0.505001251	0.45	0.432	5.81E-21	Tcell
LRRFIP1	8.04E-25	0.477378002	0.47	0.449	1.72E-20	Tcell
RARRES3	8.91E-25	0.573161067	0.27	0.214	1.90E-20	Tcell
NOP53	8.21E-24	0.501789715	0.471	0.473	1.75E-19	Tcell
DHX36	1.63E-23	0.626835985	0.317	0.275	3.48E-19	Tcell
RSRP1	1.67E-22	0.644728461	0.313	0.274	3.56E-18	Tcell
TOMM7	4.26E-22	0.26698673	0.651	0.69	9.09E-18	Tcell
STAT3	1.19E-21	0.654284931	0.342	0.312	2.54E-17	Tcell
SFPQ	1.88E-21	0.562125618	0.416	0.404	4.01E-17	Tcell
HMGB2	4.42E-20	0.263654477	0.294	0.239	9.43E-16	Tcell
FOXP1	7.40E-20	0.614258981	0.338	0.308	1.58E-15	Tcell
SRSF2	8.13E-20	0.514754403	0.43	0.427	1.74E-15	Tcell
NPM1	5.63E-19	0.320440193	0.544	0.564	1.20E-14	Tcell
XIST	4.76E-18	0.595279813	0.321	0.285	1.02E-13	Tcell
ZC3HAV1	8.44E-18	0.489172734	0.258	0.214	1.80E-13	Tcell
NAP1L1	2.00E-17	0.293262148	0.555	0.559	4.28E-13	Tcell
FXYD5	1.12E-15	0.46311974	0.331	0.307	2.39E-11	Tcell
YWHAB	5.52E-15	0.450980749	0.446	0.462	1.18E-10	Tcell
ARGLU1	1.55E-14	0.440992675	0.41	0.411	3.30E-10	Tcell
ARPC2	2.78E-14	0.265189898	0.567	0.57	5.92E-10	Tcell
MBNL1	4.72E-14	0.546251368	0.318	0.301	1.01E-09	Tcell
LDHB	8.23E-14	0.483749332	0.308	0.289	1.76E-09	Tcell
CHD1	1.10E-13	0.435440494	0.274	0.24	2.36E-09	Tcell
SUB1	6.12E-13	0.383100624	0.462	0.49	1.31E-08	Tcell
SYAP1	1.30E-12	0.429959301	0.362	0.348	2.78E-08	Tcell
ARF6	2.27E-12	0.454553458	0.347	0.339	4.84E-08	Tcell
SCAF11	3.41E-12	0.480231816	0.349	0.344	7.28E-08	Tcell
JAK1	2.00E-11	0.536658231	0.332	0.329	4.27E-07	Tcell
NCOR1	9.12E-11	0.554268239	0.254	0.237	1.95E-06	Tcell
AMD1	3.50E-10	0.495577091	0.289	0.274	7.47E-06	Tcell
SON	1.00E-09	0.30440591	0.481	0.51	2.14E-05	Tcell
MCL1	2.11E-09	0.401583724	0.461	0.492	4.50E-05	Tcell
CRIP1	3.29E-09	0.323494951	0.352	0.331	7.03E-05	Tcell
ATP1B3	5.39E-09	0.530646007	0.345	0.344	0.000115	Tcell
HINT1	6.39E-09	0.262146371	0.526	0.569	0.000136	Tcell
TNRC6B	1.31E-08	0.479722278	0.261	0.247	0.000279	Tcell
PARK7	2.27E-08	0.253140176	0.254	0.349	0.000486	Tcell
PNISR	2.95E-08	0.47412054	0.335	0.338	0.00063	Tcell
TXNIP	5.28E-08	0.428385206	0.377	0.372	0.001126	Tcell

DYNC1H1	5.06E-07	0.546701947	0.253	0.248	0.010802	Tcell
UBE2B	9.21E-07	0.445958923	0.32	0.332	0.019667	Tcell
KLF6	2.03E-06	0.301784084	0.538	0.566	0.043363	Tcell
DDX46	5.69E-06	0.465365457	0.269	0.268	0.121415	Tcell
SRSF11	8.30E-06	0.41388745	0.361	0.386	0.177162	Tcell
IQGAP1	8.58E-06	0.515624147	0.296	0.31	0.183245	Tcell
COMMD6	3.19E-05	0.269635232	0.461	0.508	0.681595	Tcell
ANKRD11	3.80E-05	0.441835182	0.319	0.332	0.810669	Tcell
RBMX	0.000109	0.279630006	0.396	0.414	1	Tcell
SLTM	0.000214	0.401841851	0.258	0.261	1	Tcell
SRRM1	0.000288	0.300345044	0.386	0.417	1	Tcell
CLK1	0.000845	0.283315963	0.323	0.337	1	Tcell
CIB1	0.001212	0.390185594	0.285	0.299	1	Tcell
CYTOR	0.001888	0.368560661	0.292	0.305	1	Tcell
H2AFV	0.002868	0.382245435	0.273	0.288	1	Tcell
MPHOSPH	0.003257	0.411725295	0.248	0.256	1	Tcell
RBM25	0.003533	0.311468124	0.366	0.403	1	Tcell
BCLAF1	0.003703	0.443911169	0.242	0.25	1	Tcell
HIGD2A	0.003833	0.258135427	0.266	0.338	1	Tcell
PTP4A2	0.004126	0.338411167	0.275	0.352	1	Tcell
CDV3	0.005297	0.459287359	0.314	0.345	1	Tcell
TAX1BP1	0.006576	0.271280688	0.219	0.273	1	Tcell
ANXA1	0.008369	0.352627724	0.299	0.369	1	Tcell
COL1A1	0	5.335655797	0.668	0.048	0	Fibroblasts
DCN	0	5.126619988	0.832	0.027	0	Fibroblasts
COL1A2	0	5.003860796	0.762	0.045	0	Fibroblasts
CCDC80	0	4.777859363	0.712	0.017	0	Fibroblasts
MGP	0	4.648782592	0.665	0.086	0	Fibroblasts
COL3A1	0	4.597211904	0.63	0.026	0	Fibroblasts
CALD1	0	4.483006092	0.925	0.078	0	Fibroblasts
LUM	0	4.402529526	0.77	0.034	0	Fibroblasts
CXCL14	0	4.160502672	0.32	0.021	0	Fibroblasts
TAGLN	0	3.974686182	0.571	0.036	0	Fibroblasts
CFD	0	3.913003367	0.377	0.052	0	Fibroblasts
FBLN1	0	3.808143693	0.646	0.014	0	Fibroblasts
APOD	0	3.657831592	0.277	0.008	0	Fibroblasts
GSN	0	3.485302853	0.613	0.105	0	Fibroblasts
IGFBP7	0	3.460971452	0.881	0.135	0	Fibroblasts
SPARC	0	3.420756671	0.719	0.095	0	Fibroblasts
IGFBP6	0	3.353408201	0.535	0.02	0	Fibroblasts
BGN	0	3.34117761	0.665	0.032	0	Fibroblasts
RARRES2	0	3.309260397	0.692	0.021	0	Fibroblasts
TPM2	0	3.281035581	0.634	0.04	0	Fibroblasts

CTGF	0	3.252734474	0.692	0.063	0	Fibroblasts
IGFBP5	0	3.247059565	0.553	0.025	0	Fibroblasts
SFRP4	0	3.178390611	0.379	0.004	0	Fibroblasts
MEG3	0	3.174974204	0.598	0.01	0	Fibroblasts
C11orf96	0	3.140702292	0.695	0.052	0	Fibroblasts
SERPINF1	0	3.135768223	0.666	0.02	0	Fibroblasts
COL6A2	0	3.067678151	0.752	0.044	0	Fibroblasts
MYL9	0	3.048582007	0.588	0.036	0	Fibroblasts
C1R	0	2.972897632	0.696	0.016	0	Fibroblasts
COL6A3	0	2.969077859	0.641	0.019	0	Fibroblasts
ASPN	0	2.965576525	0.454	0.002	0	Fibroblasts
SFRP2	0	2.939581846	0.431	0.004	0	Fibroblasts
C7	0	2.90809222	0.304	0.01	0	Fibroblasts
TIMP1	0	2.869029507	0.852	0.238	0	Fibroblasts
SPARCL1	0	2.85459622	0.75	0.076	0	Fibroblasts
C1S	0	2.853692731	0.696	0.016	0	Fibroblasts
TIMP3	0	2.851693912	0.637	0.047	0	Fibroblasts
IGFBP4	0	2.835079903	0.744	0.076	0	Fibroblasts
TPM1	0	2.805765941	0.712	0.133	0	Fibroblasts
MMP2	0	2.777380482	0.642	0.039	0	Fibroblasts
ACTA2	0	2.776311144	0.343	0.026	0	Fibroblasts
COL6A1	0	2.755532059	0.655	0.033	0	Fibroblasts
CYR61	0	2.738326133	0.586	0.047	0	Fibroblasts
THY1	0	2.703622124	0.583	0.014	0	Fibroblasts
SOD3	0	2.653636744	0.593	0.009	0	Fibroblasts
FN1	0	2.631701225	0.546	0.033	0	Fibroblasts
AEBP1	0	2.570978833	0.585	0.016	0	Fibroblasts
NNMT	0	2.568428687	0.695	0.049	0	Fibroblasts
C3	0	2.543669229	0.387	0.013	0	Fibroblasts
SERPING1	0	2.502675673	0.692	0.038	0	Fibroblasts
LGALS1	0	2.499170925	0.943	0.421	0	Fibroblasts
CTHRC1	0	2.486706848	0.414	0.023	0	Fibroblasts
MFAP4	0	2.466306815	0.552	0.006	0	Fibroblasts
EGR1	0	2.451206383	0.724	0.165	0	Fibroblasts
IFITM3	0	2.391864046	0.93	0.229	0	Fibroblasts
PTGDS	0	2.390900979	0.277	0.031	0	Fibroblasts
COL5A2	0	2.321916151	0.459	0.012	0	Fibroblasts
DPT	0	2.309702699	0.382	0.002	0	Fibroblasts
PLAC9	0	2.303255091	0.486	0.013	0	Fibroblasts
CTSK	0	2.296460713	0.528	0.014	0	Fibroblasts
SELENOM	0	2.276635371	0.732	0.111	0	Fibroblasts
MT1M	0	2.263854527	0.268	0.024	0	Fibroblasts
CRYAB	0	2.251811856	0.282	0.013	0	Fibroblasts

VCAN	0	2.242458648	0.53	0.031	0	Fibroblasts
OGN	0	2.209256358	0.332	0.002	0	Fibroblasts
FSTL1	0	2.159162921	0.586	0.036	0	Fibroblasts
CEBPD	0	2.151995938	0.69	0.137	0	Fibroblasts
FOS	0	2.150905428	0.858	0.449	0	Fibroblasts
CST3	0	2.134929492	0.877	0.262	0	Fibroblasts
TIMP2	0	2.119019246	0.655	0.071	0	Fibroblasts
PCOLCE	0	2.108725801	0.503	0.008	0	Fibroblasts
FILIP1L	0	2.106708935	0.546	0.055	0	Fibroblasts
INHBA	0	2.090928245	0.481	0.024	0	Fibroblasts
THBS1	0	2.069453777	0.544	0.087	0	Fibroblasts
CRISPLD2	0	2.055672332	0.517	0.016	0	Fibroblasts
DSTN	0	2.044400988	0.85	0.322	0	Fibroblasts
NBL1	0	2.022646535	0.578	0.083	0	Fibroblasts
RND3	0	2.018705396	0.495	0.038	0	Fibroblasts
TCEAL9	0	2.003603055	0.651	0.089	0	Fibroblasts
LHFPL6	0	1.954652135	0.488	0.031	0	Fibroblasts
CD63	0	1.944159105	0.861	0.339	0	Fibroblasts
NUPR1	0	1.929099471	0.552	0.032	0	Fibroblasts
PALLD	0	1.902729362	0.505	0.03	0	Fibroblasts
POSTN	0	1.888652152	0.296	0.029	0	Fibroblasts
EMP1	0	1.876781446	0.583	0.111	0	Fibroblasts
CAVIN1	0	1.872569359	0.582	0.062	0	Fibroblasts
COL12A1	0	1.832166378	0.355	0.008	0	Fibroblasts
MFGE8	0	1.830727365	0.533	0.043	0	Fibroblasts
IGF1	0	1.828920013	0.293	0.016	0	Fibroblasts
SPON2	0	1.82302956	0.552	0.071	0	Fibroblasts
PRRX1	0	1.801623137	0.434	0.002	0	Fibroblasts
NEXN	0	1.799496115	0.409	0.005	0	Fibroblasts
CNN3	0	1.790440427	0.553	0.048	0	Fibroblasts
PMP22	0	1.784472157	0.526	0.064	0	Fibroblasts
OLFML3	0	1.757713727	0.401	0.009	0	Fibroblasts
MYLK	0	1.757596597	0.339	0.014	0	Fibroblasts
S100A13	0	1.753100603	0.567	0.087	0	Fibroblasts
GREM1	0	1.731593227	0.259	0.004	0	Fibroblasts
HTRA1	0	1.710584759	0.428	0.039	0	Fibroblasts
HTRA3	0	1.694712055	0.341	0.006	0	Fibroblasts
COL5A1	0	1.692493525	0.409	0.007	0	Fibroblasts
MXRA8	0	1.686103382	0.448	0.004	0	Fibroblasts
MAP1B	0	1.680048956	0.368	0.027	0	Fibroblasts
THBS2	0	1.665838832	0.315	0.002	0	Fibroblasts
COL14A1	0	1.664314304	0.387	0.002	0	Fibroblasts
FBN1	0	1.652576679	0.426	0.014	0	Fibroblasts

NR2F1	0	1.650196489	0.339	0.009	0	Fibroblasts
MFAP5	0	1.646512171	0.266	0.003	0	Fibroblasts
LRP1	0	1.623303634	0.445	0.025	0	Fibroblasts
LAPTM4A	0	1.622061654	0.688	0.198	0	Fibroblasts
SERPINE2	0	1.61405794	0.324	0.026	0	Fibroblasts
PDGFRA	0	1.607826732	0.367	0.006	0	Fibroblasts
SULF1	0	1.602449512	0.353	0.008	0	Fibroblasts
PDLIM3	0	1.588972018	0.423	0.01	0	Fibroblasts
A2M	0	1.577755575	0.438	0.074	0	Fibroblasts
YBX3	0	1.565140655	0.697	0.174	0	Fibroblasts
NFIA	0	1.552781426	0.373	0.042	0	Fibroblasts
DIO2	0	1.538692263	0.305	0.003	0	Fibroblasts
COX7A1	0	1.538344797	0.45	0.031	0	Fibroblasts
CAV1	0	1.529716486	0.486	0.06	0	Fibroblasts
GEM	0	1.512274185	0.443	0.053	0	Fibroblasts
DAB2	0	1.510575963	0.486	0.062	0	Fibroblasts
BEX3	0	1.503133087	0.544	0.074	0	Fibroblasts
PPIC	0	1.500704809	0.445	0.037	0	Fibroblasts
MARCKS	0	1.495975578	0.484	0.116	0	Fibroblasts
CFH	0	1.488923194	0.313	0.019	0	Fibroblasts
LTBP4	0	1.487718614	0.405	0.039	0	Fibroblasts
CALU	0	1.484693796	0.518	0.09	0	Fibroblasts
TSC22D1	0	1.484154546	0.519	0.111	0	Fibroblasts
NFIC	0	1.480687356	0.517	0.068	0	Fibroblasts
PDPN	0	1.471610042	0.338	0.011	0	Fibroblasts
ADAMTS1	0	1.469873079	0.316	0.027	0	Fibroblasts
COL18A1	0	1.468637	0.354	0.043	0	Fibroblasts
SSPN	0	1.463382053	0.384	0.012	0	Fibroblasts
SDC2	0	1.450530836	0.422	0.023	0	Fibroblasts
CAVIN3	0	1.435760078	0.425	0.041	0	Fibroblasts
NR4A1	0	1.434926754	0.629	0.201	0	Fibroblasts
EPB41L2	0	1.412724703	0.426	0.054	0	Fibroblasts
AKAP12	0	1.412337556	0.442	0.028	0	Fibroblasts
PRSS23	0	1.411098403	0.37	0.055	0	Fibroblasts
RAB31	0	1.408313695	0.385	0.062	0	Fibroblasts
FGFR1	0	1.400643368	0.426	0.032	0	Fibroblasts
MRC2	0	1.391174683	0.381	0.008	0	Fibroblasts
OMD	0	1.380458903	0.261	0	0	Fibroblasts
COL4A2	0	1.380276974	0.378	0.059	0	Fibroblasts
EID1	0	1.376978218	0.67	0.228	0	Fibroblasts
TMEM176A	0	1.366432957	0.411	0.061	0	Fibroblasts
TMEM176B	0	1.366323602	0.414	0.059	0	Fibroblasts
ANXA5	0	1.364005167	0.613	0.171	0	Fibroblasts

FHL1	0	1.360023819	0.352	0.015	0	Fibroblasts
COL4A1	0	1.358416949	0.384	0.057	0	Fibroblasts
COL8A1	0	1.352521698	0.334	0.004	0	Fibroblasts
ANTXR1	0	1.336408277	0.333	0.004	0	Fibroblasts
ELN	0	1.333887843	0.283	0.01	0	Fibroblasts
FXYD1	0	1.310303036	0.308	0.02	0	Fibroblasts
SERPINH1	0	1.303461647	0.489	0.075	0	Fibroblasts
MXRA5	0	1.298342962	0.324	0.004	0	Fibroblasts
EMILIN1	0	1.292165154	0.321	0.005	0	Fibroblasts
SPATS2L	0	1.290579771	0.508	0.119	0	Fibroblasts
PDGFRB	0	1.284520931	0.321	0.008	0	Fibroblasts
LGALS3BP	0	1.281752379	0.471	0.075	0	Fibroblasts
ISLR	0	1.279067265	0.322	0.003	0	Fibroblasts
11-Sep	0	1.271064157	0.475	0.086	0	Fibroblasts
FERMT2	0	1.269974053	0.38	0.026	0	Fibroblasts
APP	0	1.263745965	0.511	0.103	0	Fibroblasts
TCEAL4	0	1.263342366	0.445	0.075	0	Fibroblasts
TFPI	0	1.258029354	0.365	0.037	0	Fibroblasts
LTBP1	0	1.251790045	0.312	0.011	0	Fibroblasts
DKK3	0	1.2501154	0.365	0.009	0	Fibroblasts
SVIL	0	1.238765869	0.393	0.037	0	Fibroblasts
AXL	0	1.237570257	0.335	0.023	0	Fibroblasts
RHOBTB3	0	1.235205798	0.359	0.024	0	Fibroblasts
NR2F2	0	1.231355204	0.358	0.035	0	Fibroblasts
FBLN2	0	1.229564539	0.327	0.01	0	Fibroblasts
PLAT	0	1.209006704	0.309	0.032	0	Fibroblasts
EFEMP2	0	1.183827005	0.352	0.007	0	Fibroblasts
ZFHX3	0	1.182393415	0.338	0.046	0	Fibroblasts
LMCD1	0	1.172349505	0.3	0.034	0	Fibroblasts
CLEC11A	0	1.166291351	0.332	0.013	0	Fibroblasts
ACTN1	0	1.166238132	0.394	0.068	0	Fibroblasts
CPQ	0	1.161713288	0.37	0.049	0	Fibroblasts
FAM114A1	0	1.160740106	0.365	0.025	0	Fibroblasts
PLXDC2	0	1.136496064	0.333	0.032	0	Fibroblasts
PODN	0	1.130758913	0.287	0.001	0	Fibroblasts
CDH11	0	1.129023189	0.307	0.005	0	Fibroblasts
TCF4	0	1.115776046	0.539	0.101	0	Fibroblasts
GUCY1A1	0	1.111908047	0.297	0.005	0	Fibroblasts
CYBRD1	0	1.107815681	0.3	0.012	0	Fibroblasts
PLTP	0	1.106735402	0.282	0.02	0	Fibroblasts
CYB5R3	0	1.100020013	0.502	0.122	0	Fibroblasts
NID1	0	1.079542969	0.299	0.017	0	Fibroblasts
HCFC1R1	0	1.074982341	0.376	0.057	0	Fibroblasts

BICC1	0	1.070494297	0.284	0.001	0	Fibroblasts
HSPB1	0	1.069620061	0.875	0.379	0	Fibroblasts
FKBP10	0	1.05785221	0.318	0.011	0	Fibroblasts
RAB34	0	1.049938454	0.349	0.022	0	Fibroblasts
CYGB	0	1.046651765	0.272	0.007	0	Fibroblasts
KANK2	0	1.042859657	0.302	0.009	0	Fibroblasts
PLPP3	0	1.03355894	0.277	0.027	0	Fibroblasts
AC245595.	0	1.027059945	0.268	0.019	0	Fibroblasts
S100A6	0	1.016334006	0.956	0.716	0	Fibroblasts
PKIG	0	1.014918573	0.356	0.047	0	Fibroblasts
PHLDA3	0	1.012384846	0.299	0.015	0	Fibroblasts
LAMC1	0	1.011166836	0.308	0.028	0	Fibroblasts
DDR2	0	0.994702678	0.294	0.005	0	Fibroblasts
LAMA4	0	0.981563964	0.301	0.029	0	Fibroblasts
OAF	0	0.980738119	0.292	0.032	0	Fibroblasts
RCN1	0	0.979991855	0.359	0.053	0	Fibroblasts
PBX1	0	0.977264918	0.291	0.014	0	Fibroblasts
PLOD2	0	0.973966233	0.303	0.017	0	Fibroblasts
TSPAN4	0	0.95274865	0.324	0.039	0	Fibroblasts
CLMP	0	0.948323233	0.268	0.002	0	Fibroblasts
MAP3K20	0	0.939951073	0.3	0.031	0	Fibroblasts
PDLIM7	0	0.938548462	0.307	0.039	0	Fibroblasts
DST	0	0.930157926	0.316	0.038	0	Fibroblasts
RCN3	0	0.923992836	0.258	0.004	0	Fibroblasts
ENAH	0	0.906351086	0.26	0.018	0	Fibroblasts
CERCAM	0	0.904494593	0.28	0.013	0	Fibroblasts
TGFB1I1	0	0.899875705	0.265	0.013	0	Fibroblasts
ITGB5	0	0.882610498	0.257	0.017	0	Fibroblasts
PDLIM4	0	0.881480395	0.273	0.03	0	Fibroblasts
RBPMS	0	0.861182898	0.258	0.024	0	Fibroblasts
INAFM1	0	0.855059634	0.28	0.02	0	Fibroblasts
DPYSL3	0	0.816421003	0.263	0.021	0	Fibroblasts
C9orf3	0	0.815360296	0.272	0.026	0	Fibroblasts
RTL8C	0	0.805016307	0.297	0.038	0	Fibroblasts
GNG11	0	0.794406953	0.376	0.06	0	Fibroblasts
PDLIM2	1.15E-306	0.850891094	0.352	0.057	2.46E-302	Fibroblasts
CRTAP	3.95E-306	0.959744759	0.381	0.069	8.43E-302	Fibroblasts
GPNMB	1.26E-304	0.788376208	0.295	0.037	2.69E-300	Fibroblasts
GOLIM4	4.30E-304	0.997144047	0.416	0.084	9.18E-300	Fibroblasts
CKAP4	2.85E-300	0.836919317	0.314	0.046	6.09E-296	Fibroblasts
EMP2	5.48E-298	0.930596729	0.312	0.046	1.17E-293	Fibroblasts
ITGB1	5.33E-290	1.279136299	0.742	0.308	1.14E-285	Fibroblasts
F3	5.48E-290	1.361087081	0.265	0.032	1.17E-285	Fibroblasts

MT1E	1.99E-288	1.804426144	0.492	0.126	4.24E-284	Fibroblasts
HSPA1A	1.78E-287	1.094210188	0.756	0.289	3.80E-283	Fibroblasts
ATF3	1.83E-287	1.34177503	0.545	0.159	3.90E-283	Fibroblasts
UACA	2.25E-287	1.024349276	0.274	0.035	4.81E-283	Fibroblasts
CEBPB	5.85E-283	1.361074641	0.71	0.272	1.25E-278	Fibroblasts
SELENOP	3.86E-281	1.043518497	0.294	0.043	8.23E-277	Fibroblasts
LEPROT	5.33E-281	0.922217071	0.417	0.091	1.14E-276	Fibroblasts
TUBB6	7.94E-280	0.702556675	0.26	0.031	1.69E-275	Fibroblasts
FOSB	2.01E-277	1.687499019	0.782	0.407	4.29E-273	Fibroblasts
UGDH	1.40E-273	1.14163117	0.279	0.039	2.98E-269	Fibroblasts
VKORC1	3.74E-273	0.967561251	0.439	0.106	7.99E-269	Fibroblasts
VIM	1.52E-272	1.230246275	0.891	0.559	3.25E-268	Fibroblasts
NENF	5.49E-271	1.004101505	0.544	0.162	1.17E-266	Fibroblasts
PTMS	1.09E-269	1.119106431	0.61	0.203	2.33E-265	Fibroblasts
MT2A	1.26E-269	2.994040958	0.778	0.399	2.69E-265	Fibroblasts
TNFRSF12/	9.18E-268	1.000379407	0.265	0.035	1.96E-263	Fibroblasts
KCNQ1OT	2.40E-266	1.369674104	0.459	0.118	5.11E-262	Fibroblasts
NFIB	1.85E-264	1.103587782	0.308	0.051	3.95E-260	Fibroblasts
SPRY1	2.04E-264	1.00434557	0.457	0.11	4.35E-260	Fibroblasts
KLF9	2.20E-264	0.972627741	0.414	0.093	4.69E-260	Fibroblasts
PAM	1.58E-258	0.710753021	0.269	0.038	3.37E-254	Fibroblasts
REXO2	1.61E-258	0.922131931	0.417	0.098	3.44E-254	Fibroblasts
NUCKS1	1.52E-256	1.160000677	0.674	0.263	3.24E-252	Fibroblasts
TGFBI	1.47E-255	0.990475156	0.297	0.048	3.13E-251	Fibroblasts
ID3	3.55E-253	1.286362563	0.457	0.12	7.57E-249	Fibroblasts
JUN	1.73E-251	1.624773821	0.853	0.527	3.69E-247	Fibroblasts
MMP14	6.72E-251	0.82297625	0.273	0.041	1.43E-246	Fibroblasts
IL1R1	2.16E-250	0.81050435	0.28	0.043	4.61E-246	Fibroblasts
LIMA1	2.74E-249	1.007472249	0.428	0.107	5.85E-245	Fibroblasts
F2R	7.31E-248	0.92611989	0.322	0.059	1.56E-243	Fibroblasts
TAX1BP3	7.53E-247	0.791370426	0.328	0.063	1.61E-242	Fibroblasts
RHOB	1.18E-246	1.261138854	0.553	0.178	2.51E-242	Fibroblasts
VCL	1.25E-246	0.775834705	0.294	0.049	2.66E-242	Fibroblasts
GADD45B	2.17E-245	1.448229499	0.595	0.214	4.63E-241	Fibroblasts
MDK	1.06E-243	0.862593305	0.362	0.077	2.27E-239	Fibroblasts
PPFIBP1	2.24E-243	0.655707511	0.251	0.035	4.78E-239	Fibroblasts
LMO4	9.43E-241	0.977697578	0.53	0.163	2.01E-236	Fibroblasts
CD81	5.48E-240	0.977889427	0.52	0.156	1.17E-235	Fibroblasts
ERRFI1	3.08E-237	0.919192346	0.304	0.055	6.57E-233	Fibroblasts
CCL2	5.61E-234	1.672566667	0.252	0.038	1.20E-229	Fibroblasts
SERPINE1	2.11E-233	1.545063095	0.31	0.059	4.50E-229	Fibroblasts
KLF4	3.88E-233	1.263100307	0.404	0.104	8.27E-229	Fibroblasts
ZFP36L1	8.60E-233	1.286627591	0.727	0.324	1.84E-228	Fibroblasts

ADIRF	1.16E-232	2.19442643	0.438	0.123	2.47E-228	Fibroblasts
RRBP1	3.38E-230	1.103227885	0.632	0.247	7.21E-226	Fibroblasts
ABL2	2.67E-229	1.347596516	0.39	0.096	5.69E-225	Fibroblasts
PHLDA1	6.02E-229	1.256440113	0.478	0.144	1.29E-224	Fibroblasts
CD151	1.42E-228	0.90126379	0.402	0.102	3.03E-224	Fibroblasts
HES1	3.35E-228	1.132726882	0.48	0.141	7.15E-224	Fibroblasts
TCEAL8	9.46E-226	0.837291479	0.373	0.088	2.02E-221	Fibroblasts
MXRA7	1.41E-224	0.59539245	0.275	0.046	3.01E-220	Fibroblasts
FHL2	3.44E-221	0.717863825	0.289	0.053	7.35E-217	Fibroblasts
HES4	2.51E-220	0.946279048	0.324	0.068	5.37E-216	Fibroblasts
ZBTB20	2.89E-220	0.959212386	0.506	0.157	6.17E-216	Fibroblasts
DDAH2	9.80E-219	0.804166748	0.334	0.074	2.09E-214	Fibroblasts
CSRP1	4.70E-216	1.004117879	0.292	0.057	1.00E-211	Fibroblasts
PDLIM1	3.52E-215	0.8587421	0.435	0.119	7.52E-211	Fibroblasts
IFI27	3.98E-215	1.037600038	0.61	0.228	8.50E-211	Fibroblasts
RTN4	3.43E-214	1.045458418	0.604	0.233	7.33E-210	Fibroblasts
MT1X	1.74E-213	2.877131656	0.471	0.159	3.71E-209	Fibroblasts
DPYSL2	4.89E-209	0.830499678	0.341	0.079	1.04E-204	Fibroblasts
HSPA1B	8.53E-207	0.822346297	0.65	0.249	1.82E-202	Fibroblasts
SMIM3	1.05E-204	0.74221631	0.275	0.051	2.25E-200	Fibroblasts
ITGAV	1.97E-204	0.738319115	0.286	0.057	4.20E-200	Fibroblasts
ARF4	2.82E-204	0.896120521	0.517	0.177	6.01E-200	Fibroblasts
COL15A1	7.73E-203	0.719824564	0.25	0.042	1.65E-198	Fibroblasts
CD9	1.26E-200	0.99095129	0.493	0.159	2.69E-196	Fibroblasts
LPP	1.64E-198	1.052503685	0.426	0.127	3.51E-194	Fibroblasts
TSHZ2	2.84E-198	0.8679076	0.391	0.104	6.05E-194	Fibroblasts
CTSL	3.57E-198	0.706948982	0.364	0.09	7.61E-194	Fibroblasts
CAMK2N1	4.76E-196	0.73993802	0.296	0.062	1.02E-191	Fibroblasts
TUBA1A	1.16E-194	1.038948587	0.483	0.16	2.47E-190	Fibroblasts
ANXA2	2.90E-194	0.993014001	0.748	0.36	6.20E-190	Fibroblasts
CLU	3.93E-194	1.581317576	0.316	0.075	8.40E-190	Fibroblasts
RAB13	2.21E-193	0.882742137	0.381	0.104	4.71E-189	Fibroblasts
C12orf57	6.39E-193	0.891242634	0.659	0.282	1.36E-188	Fibroblasts
LMNA	3.77E-190	0.961255475	0.734	0.354	8.05E-186	Fibroblasts
SOX4	4.27E-189	0.734877618	0.484	0.158	9.11E-185	Fibroblasts
TMEM14C	7.53E-189	0.678369215	0.395	0.113	1.61E-184	Fibroblasts
MGST3	6.06E-188	0.843104437	0.496	0.17	1.29E-183	Fibroblasts
ITM2B	9.04E-188	1.03727455	0.786	0.448	1.93E-183	Fibroblasts
MAFB	3.38E-187	0.861959049	0.268	0.054	7.22E-183	Fibroblasts
CD59	5.53E-187	0.753629459	0.434	0.131	1.18E-182	Fibroblasts
SPTBN1	1.04E-185	0.967225199	0.422	0.13	2.23E-181	Fibroblasts
YIF1A	5.41E-184	0.593343271	0.301	0.069	1.16E-179	Fibroblasts
VGLL4	1.07E-182	0.700646571	0.26	0.052	2.27E-178	Fibroblasts

WASL	1.67E-182	0.554433448	0.257	0.051	3.57E-178	Fibroblasts
MAP4	2.46E-181	0.681971003	0.36	0.096	5.25E-177	Fibroblasts
FOXO3	2.50E-181	0.765503425	0.343	0.089	5.35E-177	Fibroblasts
C1orf21	1.08E-179	0.69097131	0.304	0.07	2.30E-175	Fibroblasts
ARL2	3.02E-179	0.601381047	0.298	0.069	6.45E-175	Fibroblasts
ILK	4.57E-178	0.59635259	0.302	0.07	9.75E-174	Fibroblasts
PSAP	2.67E-177	0.567162435	0.653	0.271	5.70E-173	Fibroblasts
CRIP2	1.08E-174	0.667128132	0.303	0.07	2.32E-170	Fibroblasts
SPTSSA	1.48E-173	0.682816915	0.297	0.071	3.17E-169	Fibroblasts
CLIC4	2.13E-173	0.6763103	0.255	0.053	4.56E-169	Fibroblasts
SOD2	4.10E-171	0.991054898	0.495	0.183	8.75E-167	Fibroblasts
FAM3C	2.15E-170	0.660034415	0.351	0.098	4.59E-166	Fibroblasts
RUNX1	1.18E-169	0.611934439	0.293	0.069	2.52E-165	Fibroblasts
SCARB2	1.60E-169	0.618705805	0.304	0.075	3.42E-165	Fibroblasts
DUSP1	1.75E-168	1.151745185	0.711	0.376	3.73E-164	Fibroblasts
LY6E	4.35E-168	0.876794544	0.528	0.203	9.28E-164	Fibroblasts
GOLGA2	6.85E-168	0.732014964	0.376	0.111	1.46E-163	Fibroblasts
KDELR2	2.94E-167	0.84215223	0.475	0.175	6.28E-163	Fibroblasts
GINM1	2.50E-166	0.550915426	0.272	0.061	5.33E-162	Fibroblasts
PPIB	2.85E-166	1.003369305	0.658	0.331	6.08E-162	Fibroblasts
CTTN	1.62E-165	0.631142888	0.258	0.056	3.46E-161	Fibroblasts
PLD3	1.49E-164	0.565254785	0.286	0.068	3.19E-160	Fibroblasts
PHPT1	1.54E-162	0.784777733	0.484	0.178	3.29E-158	Fibroblasts
MTRNR2L8	1.25E-161	0.669631472	0.308	0.079	2.66E-157	Fibroblasts
FCGRT	8.13E-161	0.657248042	0.364	0.107	1.73E-156	Fibroblasts
H2AFJ	4.97E-160	0.660689506	0.384	0.117	1.06E-155	Fibroblasts
BSG	1.13E-155	0.742841385	0.512	0.198	2.42E-151	Fibroblasts
DYNC1I2	5.09E-155	0.704455593	0.437	0.151	1.09E-150	Fibroblasts
RHOC	1.30E-154	0.686451555	0.469	0.169	2.77E-150	Fibroblasts
IER2	2.06E-154	1.217529978	0.607	0.297	4.39E-150	Fibroblasts
HDLBP	2.48E-153	0.688386168	0.39	0.129	5.30E-149	Fibroblasts
ESD	6.72E-153	0.642914178	0.323	0.091	1.43E-148	Fibroblasts
BAG3	1.00E-152	0.685336258	0.357	0.107	2.14E-148	Fibroblasts
SOCS3	1.37E-152	1.036074392	0.412	0.145	2.93E-148	Fibroblasts
MORF4L2	1.01E-151	0.647638329	0.428	0.147	2.15E-147	Fibroblasts
RGS16	3.20E-150	1.018172252	0.278	0.071	6.82E-146	Fibroblasts
TUBB	7.30E-149	0.649973458	0.524	0.21	1.56E-144	Fibroblasts
PEBP1	1.18E-148	0.774086588	0.566	0.249	2.53E-144	Fibroblasts
TENT5A	3.67E-147	0.627659359	0.259	0.062	7.83E-143	Fibroblasts
PRDX6	2.05E-146	0.667746902	0.494	0.191	4.39E-142	Fibroblasts
LGALS3	3.39E-146	0.650674327	0.617	0.273	7.23E-142	Fibroblasts
PTTG1IP	5.81E-146	0.6728382	0.389	0.13	1.24E-141	Fibroblasts
BST2	9.58E-146	0.957872321	0.424	0.157	2.05E-141	Fibroblasts

TNFAIP2	4.86E-143	1.045216847	0.302	0.088	1.04E-138	Fibroblasts
EPS8	1.46E-142	0.580109449	0.254	0.062	3.12E-138	Fibroblasts
NUCB1	1.19E-139	0.636709109	0.351	0.112	2.53E-135	Fibroblasts
MTRNR2L1	9.19E-139	0.938270543	0.362	0.122	1.96E-134	Fibroblasts
RAB2A	4.03E-138	0.669857004	0.501	0.204	8.61E-134	Fibroblasts
MYADM	4.83E-137	0.696628867	0.533	0.221	1.03E-132	Fibroblasts
TNFRSF1A	1.11E-134	0.565232434	0.269	0.071	2.36E-130	Fibroblasts
DNAJB4	3.57E-133	0.630124404	0.275	0.074	7.61E-129	Fibroblasts
ATRAID	8.77E-133	0.614824292	0.377	0.131	1.87E-128	Fibroblasts
RABAC1	1.41E-132	0.671561401	0.568	0.256	3.01E-128	Fibroblasts
CD99	5.67E-132	0.766378225	0.602	0.287	1.21E-127	Fibroblasts
GLUL	2.67E-131	0.835178384	0.462	0.18	5.71E-127	Fibroblasts
VAMP5	1.67E-130	0.607431229	0.418	0.151	3.57E-126	Fibroblasts
TPM4	6.35E-129	0.883233727	0.6	0.299	1.36E-124	Fibroblasts
OSTC	9.50E-128	0.633199033	0.407	0.153	2.03E-123	Fibroblasts
MORF4L1	5.31E-127	0.669932999	0.693	0.367	1.13E-122	Fibroblasts
NPC2	1.90E-126	0.51605179	0.548	0.234	4.05E-122	Fibroblasts
RDX	4.39E-125	0.599829229	0.398	0.142	9.38E-121	Fibroblasts
P4HB	1.73E-124	0.673534525	0.528	0.237	3.69E-120	Fibroblasts
SELENOS	9.32E-124	0.636225282	0.546	0.242	1.99E-119	Fibroblasts
KDELR1	3.46E-123	0.587880838	0.366	0.131	7.40E-119	Fibroblasts
STOM	4.06E-123	0.725453238	0.34	0.113	8.67E-119	Fibroblasts
IER3	1.13E-122	1.010002037	0.517	0.237	2.40E-118	Fibroblasts
SEC62	1.29E-122	0.674445303	0.649	0.321	2.76E-118	Fibroblasts
VMP1	1.63E-122	0.888422225	0.522	0.233	3.48E-118	Fibroblasts
AHCYL1	1.78E-122	0.530188931	0.329	0.107	3.79E-118	Fibroblasts
CASC4	8.12E-122	0.41810068	0.252	0.068	1.73E-117	Fibroblasts
NUCB2	8.85E-121	0.522892263	0.297	0.091	1.89E-116	Fibroblasts
TMED3	3.04E-119	0.438035142	0.252	0.07	6.48E-115	Fibroblasts
GSTP1	1.26E-118	0.543877524	0.663	0.328	2.68E-114	Fibroblasts
PRDX4	5.98E-118	0.472165945	0.293	0.09	1.28E-113	Fibroblasts
ANKRD10	8.52E-118	0.495947292	0.278	0.083	1.82E-113	Fibroblasts
ITGB1BP1	2.12E-117	0.422426332	0.315	0.101	4.52E-113	Fibroblasts
RAC1	3.05E-117	0.617903588	0.712	0.379	6.52E-113	Fibroblasts
C1orf122	6.52E-117	0.478957729	0.306	0.098	1.39E-112	Fibroblasts
RNH1	1.37E-116	0.51721284	0.356	0.126	2.92E-112	Fibroblasts
RCN2	2.96E-116	0.423210907	0.274	0.08	6.33E-112	Fibroblasts
IFITM2	4.22E-116	0.587060273	0.689	0.353	9.01E-112	Fibroblasts
NORAD	4.30E-115	0.49824797	0.347	0.121	9.18E-111	Fibroblasts
DAAM1	4.31E-115	0.543842639	0.275	0.082	9.21E-111	Fibroblasts
ADI1	1.04E-114	0.494469157	0.284	0.088	2.22E-110	Fibroblasts
IFI6	2.18E-114	0.709752287	0.422	0.167	4.66E-110	Fibroblasts
ATOX1	4.18E-114	0.531169416	0.456	0.187	8.91E-110	Fibroblasts

REEP3	1.35E-113	0.47718501	0.275	0.084	2.89E-109	Fibroblasts
PMEPA1	1.65E-113	0.595728173	0.341	0.118	3.52E-109	Fibroblasts
SRM	6.65E-113	0.472882671	0.298	0.096	1.42E-108	Fibroblasts
FUNDC2	7.97E-113	0.445219064	0.29	0.091	1.70E-108	Fibroblasts
HEXB	1.29E-112	0.47505041	0.269	0.081	2.75E-108	Fibroblasts
NEAT1	7.38E-112	0.613370192	0.957	0.831	1.57E-107	Fibroblasts
TMED2	9.30E-112	0.585094922	0.496	0.22	1.99E-107	Fibroblasts
SEC31A	2.47E-110	0.434330079	0.307	0.101	5.28E-106	Fibroblasts
IL6ST	1.20E-109	0.551742412	0.373	0.139	2.55E-105	Fibroblasts
PDLIM5	9.58E-109	0.565185081	0.275	0.087	2.04E-104	Fibroblasts
NSRP1	1.97E-108	0.562832752	0.329	0.115	4.20E-104	Fibroblasts
HSBP1	2.00E-108	0.4807465	0.401	0.158	4.27E-104	Fibroblasts
ZBTB16	3.18E-108	0.599651663	0.252	0.073	6.80E-104	Fibroblasts
CCPG1	5.44E-108	0.471797574	0.261	0.079	1.16E-103	Fibroblasts
OAT	6.85E-108	0.445679909	0.25	0.073	1.46E-103	Fibroblasts
PRDX5	3.14E-107	0.495896179	0.52	0.233	6.71E-103	Fibroblasts
RAB7A	8.68E-107	0.578269949	0.374	0.144	1.85E-102	Fibroblasts
S100A16	1.01E-106	0.458862717	0.329	0.115	2.16E-102	Fibroblasts
ATP6AP2	1.16E-106	0.455004658	0.327	0.114	2.47E-102	Fibroblasts
DNAJB1	7.94E-106	0.52870677	0.751	0.462	1.69E-101	Fibroblasts
ERLEC1	1.04E-105	0.500118851	0.378	0.146	2.23E-101	Fibroblasts
CYB5A	1.21E-105	0.496943308	0.276	0.089	2.58E-101	Fibroblasts
RBMS1	5.33E-105	0.423643463	0.382	0.143	1.14E-100	Fibroblasts
MTCH1	1.66E-104	0.445697466	0.406	0.162	3.54E-100	Fibroblasts
NDUFC2	8.25E-104	0.520947106	0.409	0.166	1.76E-99	Fibroblasts
ZEB1	1.73E-103	0.403949291	0.266	0.081	3.69E-99	Fibroblasts
FNDC3B	2.71E-103	0.489218484	0.267	0.085	5.79E-99	Fibroblasts
EMP3	7.59E-103	0.629382889	0.509	0.232	1.62E-98	Fibroblasts
DESI2	1.51E-101	0.351914096	0.25	0.075	3.23E-97	Fibroblasts
NFKBIZ	1.75E-100	0.665884716	0.424	0.18	3.73E-96	Fibroblasts
MAP1LC3A	1.94E-100	0.399160158	0.257	0.08	4.14E-96	Fibroblasts
AP2M1	2.50E-100	0.461982611	0.406	0.166	5.33E-96	Fibroblasts
CFAP97	3.15E-100	0.401428458	0.262	0.082	6.72E-96	Fibroblasts
HSP90B1	4.63E-100	0.626189363	0.642	0.351	9.89E-96	Fibroblasts
TMEM230	1.20E-99	0.454732774	0.385	0.154	2.55E-95	Fibroblasts
ATP5PF	2.04E-99	0.500786606	0.551	0.263	4.36E-95	Fibroblasts
NUDT4	2.52E-99	0.665081931	0.387	0.159	5.38E-95	Fibroblasts
IFI27L2	3.26E-99	0.534992843	0.396	0.161	6.95E-95	Fibroblasts
CHD9	1.18E-97	0.422655634	0.286	0.096	2.53E-93	Fibroblasts
SNX3	1.85E-97	0.47095039	0.53	0.253	3.96E-93	Fibroblasts
IFITM1	2.41E-97	0.769698217	0.307	0.112	5.14E-93	Fibroblasts
LMAN1	4.46E-97	0.441227974	0.362	0.142	9.51E-93	Fibroblasts
NDUFAF3	7.30E-97	0.44329204	0.32	0.117	1.56E-92	Fibroblasts

MYDGF	9.10E-97	0.483211871	0.412	0.174	1.94E-92	Fibroblasts
MCRIP1	9.82E-97	0.326533689	0.26	0.082	2.10E-92	Fibroblasts
JUNB	1.12E-96	1.071363451	0.772	0.542	2.40E-92	Fibroblasts
PSMB5	2.27E-96	0.41874613	0.269	0.09	4.84E-92	Fibroblasts
BRI3	1.09E-94	0.452739622	0.431	0.185	2.32E-90	Fibroblasts
SERTAD1	1.99E-94	0.644660007	0.357	0.143	4.25E-90	Fibroblasts
TMEM59	1.06E-93	0.539876751	0.536	0.262	2.27E-89	Fibroblasts
UGCG	3.83E-92	0.513751683	0.304	0.111	8.17E-88	Fibroblasts
ANAPC11	7.52E-92	0.441220599	0.442	0.196	1.60E-87	Fibroblasts
HSP90AB1	7.92E-92	0.544777111	0.861	0.61	1.69E-87	Fibroblasts
SSR3	1.83E-91	0.556044151	0.454	0.208	3.90E-87	Fibroblasts
RWDD1	1.90E-91	0.417897603	0.493	0.229	4.05E-87	Fibroblasts
KDSR	2.03E-91	0.410079121	0.27	0.092	4.33E-87	Fibroblasts
CLTA	2.81E-91	0.457214591	0.471	0.214	5.99E-87	Fibroblasts
TTC3	5.10E-91	0.481146635	0.436	0.196	1.09E-86	Fibroblasts
SEC63	6.28E-91	0.384271659	0.266	0.09	1.34E-86	Fibroblasts
DAD1	1.38E-90	0.43033503	0.429	0.188	2.95E-86	Fibroblasts
UBB	1.52E-90	0.631844492	0.88	0.658	3.24E-86	Fibroblasts
ZNF106	2.32E-90	0.582304313	0.299	0.11	4.95E-86	Fibroblasts
CAMTA1	2.37E-90	0.482841792	0.332	0.13	5.06E-86	Fibroblasts
CHMP3	2.75E-90	0.469876559	0.346	0.137	5.86E-86	Fibroblasts
EIF4A1	3.24E-90	0.541789682	0.495	0.235	6.93E-86	Fibroblasts
S100A11	4.47E-90	0.454429423	0.775	0.475	9.54E-86	Fibroblasts
FLNA	5.37E-90	0.668568363	0.385	0.164	1.15E-85	Fibroblasts
C4orf3	1.07E-89	0.504171111	0.53	0.262	2.29E-85	Fibroblasts
TMBIM4	5.02E-89	0.437719023	0.381	0.159	1.07E-84	Fibroblasts
ATP2A2	8.75E-89	0.407448951	0.286	0.103	1.87E-84	Fibroblasts
NDUFA11	2.39E-88	0.471699387	0.498	0.238	5.11E-84	Fibroblasts
CHMP4B	9.09E-88	0.482380647	0.418	0.185	1.94E-83	Fibroblasts
SBDS	1.02E-87	0.445169011	0.341	0.136	2.17E-83	Fibroblasts
MIF	1.10E-86	0.569775913	0.575	0.299	2.36E-82	Fibroblasts
LAMP1	1.37E-86	0.392291983	0.381	0.159	2.93E-82	Fibroblasts
MRFAP1	1.42E-86	0.368758899	0.465	0.211	3.04E-82	Fibroblasts
PKM	1.72E-86	0.468024782	0.494	0.237	3.67E-82	Fibroblasts
TACC1	3.10E-86	0.485038062	0.427	0.189	6.62E-82	Fibroblasts
SAR1A	1.85E-85	0.374658058	0.346	0.14	3.95E-81	Fibroblasts
07-Sep	2.81E-85	0.538073117	0.549	0.286	6.00E-81	Fibroblasts
MINOS1	3.11E-85	0.462499129	0.433	0.195	6.64E-81	Fibroblasts
DDX1	7.41E-85	0.388546365	0.265	0.093	1.58E-80	Fibroblasts
RPS27L	1.29E-84	0.53407529	0.619	0.346	2.76E-80	Fibroblasts
PHACTR2	1.97E-84	0.376041181	0.3	0.112	4.21E-80	Fibroblasts
CAPZB	5.98E-83	0.442209635	0.498	0.245	1.28E-78	Fibroblasts
TMED9	4.55E-82	0.408191197	0.354	0.148	9.72E-78	Fibroblasts

CLTB	6.87E-82	0.40126036	0.362	0.153	1.47E-77	Fibroblasts
WSB1	8.82E-82	0.474671374	0.525	0.262	1.88E-77	Fibroblasts
ASPH	1.23E-81	0.3705181	0.26	0.093	2.63E-77	Fibroblasts
YWHAE	2.55E-81	0.486307974	0.555	0.283	5.45E-77	Fibroblasts
GRN	2.88E-81	0.306682866	0.335	0.135	6.14E-77	Fibroblasts
PRDX1	3.32E-81	0.384443098	0.571	0.292	7.09E-77	Fibroblasts
SELENOW	1.03E-80	0.426803054	0.565	0.291	2.20E-76	Fibroblasts
SQSTM1	1.83E-79	0.482105206	0.604	0.328	3.90E-75	Fibroblasts
LRPAP1	3.17E-79	0.407621862	0.293	0.113	6.76E-75	Fibroblasts
NEU1	3.45E-79	0.362384794	0.274	0.101	7.37E-75	Fibroblasts
NME3	1.52E-78	0.375052328	0.271	0.1	3.25E-74	Fibroblasts
DYNC1LI2	4.25E-78	0.333447505	0.262	0.096	9.06E-74	Fibroblasts
KRT10	5.36E-78	0.348707486	0.443	0.207	1.15E-73	Fibroblasts
AFF4	6.56E-78	0.347612837	0.345	0.145	1.40E-73	Fibroblasts
PPP1R15A	1.15E-77	0.460015121	0.641	0.366	2.44E-73	Fibroblasts
NDUFA4	1.65E-77	0.646853773	0.679	0.416	3.52E-73	Fibroblasts
BRK1	2.25E-77	0.383102049	0.535	0.271	4.80E-73	Fibroblasts
SLC38A2	2.37E-77	0.536899878	0.511	0.259	5.06E-73	Fibroblasts
SELENOF	2.92E-77	0.360596625	0.349	0.147	6.23E-73	Fibroblasts
RHEB	1.53E-76	0.325552217	0.385	0.17	3.26E-72	Fibroblasts
GRHPR	2.42E-76	0.347632682	0.25	0.09	5.16E-72	Fibroblasts
BNIP3L	2.77E-76	0.353710334	0.308	0.123	5.92E-72	Fibroblasts
GABARAPL	1.37E-75	0.3446592	0.428	0.2	2.92E-71	Fibroblasts
PDIA3	1.73E-75	0.372823123	0.412	0.19	3.69E-71	Fibroblasts
CTNNB1	4.14E-75	0.622014509	0.387	0.179	8.83E-71	Fibroblasts
CHURC1	9.69E-75	0.346089174	0.364	0.159	2.07E-70	Fibroblasts
DYNLT1	1.61E-74	0.342523801	0.362	0.158	3.43E-70	Fibroblasts
PSMC5	2.01E-74	0.303563481	0.3	0.119	4.29E-70	Fibroblasts
ADD3	2.29E-74	0.410423984	0.285	0.111	4.90E-70	Fibroblasts
MYL6	3.82E-74	0.651903121	0.859	0.664	8.16E-70	Fibroblasts
ZFAND5	6.88E-74	0.481292611	0.516	0.267	1.47E-69	Fibroblasts
RNASEH2C	2.48E-73	0.37941153	0.279	0.109	5.30E-69	Fibroblasts
CHMP5	3.27E-73	0.30299869	0.293	0.116	6.97E-69	Fibroblasts
SH3BGRL	3.41E-73	0.365581956	0.405	0.186	7.28E-69	Fibroblasts
C6orf48	3.43E-73	0.339041282	0.526	0.271	7.31E-69	Fibroblasts
ATP6V0E1	3.76E-73	0.434904283	0.574	0.313	8.03E-69	Fibroblasts
PSMG2	5.94E-73	0.302546698	0.268	0.102	1.27E-68	Fibroblasts
ERH	6.27E-73	0.357927358	0.451	0.217	1.34E-68	Fibroblasts
STMP1	1.63E-72	0.354339339	0.336	0.144	3.48E-68	Fibroblasts
MAFF	1.90E-72	0.610987914	0.326	0.142	4.06E-68	Fibroblasts
ARID5B1	2.11E-72	0.323379287	0.66	0.362	4.49E-68	Fibroblasts
PSMC3	1.18E-71	0.320510529	0.319	0.134	2.51E-67	Fibroblasts
TIMM13	2.78E-71	0.335191277	0.371	0.166	5.94E-67	Fibroblasts

RAB1A	4.20E-71	0.380238279	0.349	0.155	8.96E-67	Fibroblasts
C19orf70	5.54E-71	0.334926535	0.346	0.152	1.18E-66	Fibroblasts
YWHAQ	6.38E-71	0.322682085	0.426	0.202	1.36E-66	Fibroblasts
CCDC50	8.25E-71	0.333798644	0.251	0.094	1.76E-66	Fibroblasts
AHNAK	1.21E-70	0.350003137	0.51	0.261	2.58E-66	Fibroblasts
DCTN3	1.77E-70	0.31538966	0.293	0.118	3.79E-66	Fibroblasts
HIF1A	3.43E-70	0.378648835	0.348	0.153	7.32E-66	Fibroblasts
MRPL51	6.34E-70	0.316371638	0.371	0.168	1.35E-65	Fibroblasts
SEC11A	9.20E-70	0.382219902	0.481	0.245	1.96E-65	Fibroblasts
CALM2	2.57E-69	0.528686018	0.746	0.473	5.49E-65	Fibroblasts
PSMB1	3.07E-69	0.391363061	0.452	0.225	6.56E-65	Fibroblasts
GDI2	3.21E-69	0.268372436	0.346	0.151	6.86E-65	Fibroblasts
NDUFB1	7.01E-69	0.455526438	0.567	0.304	1.50E-64	Fibroblasts
STAT1	9.02E-69	0.459971414	0.292	0.121	1.93E-64	Fibroblasts
ARMCX3	1.91E-68	0.326563383	0.263	0.102	4.07E-64	Fibroblasts
NDUFB7	2.55E-68	0.361997623	0.456	0.228	5.44E-64	Fibroblasts
ASAP1	6.76E-68	0.304831789	0.251	0.096	1.44E-63	Fibroblasts
DYNLL1	1.21E-67	0.448779455	0.703	0.448	2.59E-63	Fibroblasts
LAMTOR1	1.31E-67	0.31211867	0.321	0.139	2.79E-63	Fibroblasts
ATP5F1C	3.68E-67	0.333337356	0.393	0.185	7.85E-63	Fibroblasts
POLR2F	8.78E-67	0.315379957	0.312	0.133	1.87E-62	Fibroblasts
CFDP1	1.09E-66	0.387032527	0.346	0.156	2.34E-62	Fibroblasts
MTDH	2.31E-66	0.381618853	0.6	0.339	4.92E-62	Fibroblasts
ELOC	2.97E-66	0.372833933	0.395	0.188	6.35E-62	Fibroblasts
TPI1	3.05E-66	0.388489339	0.639	0.359	6.52E-62	Fibroblasts
CDKN1A	3.35E-66	0.52346619	0.436	0.223	7.15E-62	Fibroblasts
TMEM258	3.58E-65	0.448818862	0.585	0.333	7.65E-61	Fibroblasts
02-Sep	5.06E-65	0.291745891	0.361	0.165	1.08E-60	Fibroblasts
TMEM147	5.13E-65	0.337944079	0.33	0.148	1.09E-60	Fibroblasts
SERPINB6	5.13E-65	0.33134118	0.266	0.108	1.09E-60	Fibroblasts
SARS	8.68E-65	0.400523388	0.304	0.133	1.85E-60	Fibroblasts
CITED2	8.75E-65	0.605421691	0.31	0.138	1.87E-60	Fibroblasts
SDF4	8.81E-65	0.315098606	0.29	0.122	1.88E-60	Fibroblasts
FLOT1	1.48E-64	0.281511359	0.278	0.115	3.16E-60	Fibroblasts
PSMB7	6.52E-64	0.329228599	0.343	0.156	1.39E-59	Fibroblasts
WASF2	1.94E-63	0.28894156	0.425	0.21	4.13E-59	Fibroblasts
ATP5PD	7.37E-63	0.277094162	0.417	0.204	1.57E-58	Fibroblasts
GADD45A	1.11E-62	0.375247936	0.341	0.157	2.37E-58	Fibroblasts
PHLDA2	1.18E-62	0.560259529	0.331	0.153	2.51E-58	Fibroblasts
MPG	2.29E-62	0.254170097	0.257	0.104	4.88E-58	Fibroblasts
PYURF	5.13E-62	0.250396669	0.314	0.139	1.10E-57	Fibroblasts
KRTCAP2	5.43E-62	0.407954547	0.338	0.157	1.16E-57	Fibroblasts
HP1BP3	7.97E-62	0.280825599	0.425	0.21	1.70E-57	Fibroblasts

TUBA1B	1.08E-61	0.329713495	0.547	0.305	2.31E-57	Fibroblasts
SEM1	1.13E-61	0.375716141	0.509	0.272	2.42E-57	Fibroblasts
MRPS21	1.81E-61	0.330962066	0.422	0.212	3.86E-57	Fibroblasts
TMCO1	1.86E-61	0.332555484	0.351	0.162	3.98E-57	Fibroblasts
SIVA1	5.21E-61	0.260405453	0.37	0.175	1.11E-56	Fibroblasts
ST13	1.68E-60	0.383877872	0.54	0.298	3.59E-56	Fibroblasts
TMEM165	1.75E-60	0.350217738	0.316	0.143	3.73E-56	Fibroblasts
APLP2	3.64E-60	0.430758044	0.385	0.19	7.76E-56	Fibroblasts
MESD	4.26E-60	0.301034419	0.284	0.123	9.10E-56	Fibroblasts
EIF5A	4.40E-60	0.310976308	0.439	0.223	9.39E-56	Fibroblasts
AP2S1	5.75E-60	0.334173923	0.409	0.206	1.23E-55	Fibroblasts
PDIA6	7.17E-60	0.336655542	0.365	0.177	1.53E-55	Fibroblasts
CYC1	1.28E-59	0.263816375	0.288	0.125	2.73E-55	Fibroblasts
POLR2I	1.82E-59	0.351040243	0.297	0.132	3.89E-55	Fibroblasts
CHMP2A	3.74E-59	0.28010826	0.361	0.173	7.98E-55	Fibroblasts
NDUFC1	3.87E-59	0.334338151	0.341	0.161	8.27E-55	Fibroblasts
LRP10	1.55E-58	0.253289796	0.283	0.123	3.30E-54	Fibroblasts
ATP5MC1	2.12E-58	0.250275288	0.348	0.164	4.52E-54	Fibroblasts
GPX4	3.04E-58	0.312158175	0.568	0.318	6.49E-54	Fibroblasts
VAPA	7.86E-58	0.280642727	0.473	0.249	1.68E-53	Fibroblasts
TMED10	3.44E-57	0.30047127	0.358	0.174	7.34E-53	Fibroblasts
RBX1	1.50E-56	0.291792117	0.427	0.219	3.20E-52	Fibroblasts
UQCRQ	2.16E-56	0.283840418	0.649	0.382	4.60E-52	Fibroblasts
S100A10	2.20E-56	0.559462176	0.743	0.527	4.70E-52	Fibroblasts
NDUFB4	2.94E-56	0.311775966	0.496	0.27	6.28E-52	Fibroblasts
SAT2	3.68E-56	0.250308609	0.25	0.105	7.86E-52	Fibroblasts
ISCU	6.45E-56	0.271857322	0.397	0.199	1.38E-51	Fibroblasts
SEC61G	6.72E-56	0.377458185	0.558	0.318	1.44E-51	Fibroblasts
PDCD5	7.50E-56	0.305114879	0.302	0.138	1.60E-51	Fibroblasts
WDR83OS	7.83E-56	0.272449263	0.382	0.189	1.67E-51	Fibroblasts
UBXN4	1.17E-55	0.317581635	0.48	0.26	2.49E-51	Fibroblasts
FKBP2	1.58E-55	0.279053426	0.394	0.197	3.37E-51	Fibroblasts
PPP3CA	2.33E-55	0.348009815	0.26	0.112	4.98E-51	Fibroblasts
YWHAG	2.99E-55	0.34968515	0.285	0.129	6.37E-51	Fibroblasts
ZNHIT1	4.33E-55	0.272930427	0.406	0.205	9.25E-51	Fibroblasts
ROMO1	5.03E-55	0.257767023	0.47	0.252	1.07E-50	Fibroblasts
NDUFAB1	1.92E-54	0.26950049	0.304	0.141	4.11E-50	Fibroblasts
CTSC	2.20E-54	0.56238197	0.344	0.17	4.70E-50	Fibroblasts
NMT1	2.83E-54	0.379113726	0.34	0.166	6.04E-50	Fibroblasts
FIS1	2.85E-54	0.299385599	0.382	0.194	6.09E-50	Fibroblasts
PGAM1	3.33E-54	0.260601201	0.37	0.184	7.11E-50	Fibroblasts
TRAM1	3.77E-54	0.308346471	0.359	0.178	8.06E-50	Fibroblasts
CALR	6.37E-54	0.343199449	0.572	0.337	1.36E-49	Fibroblasts

ANXA11	7.70E-54	0.311539474	0.542	0.307	1.64E-49	Fibroblasts
RALBP1	8.09E-54	0.259181816	0.322	0.152	1.73E-49	Fibroblasts
GADD45G	9.02E-54	0.258592691	0.393	0.201	1.92E-49	Fibroblasts
PSMC1	1.64E-53	0.327697845	0.338	0.164	3.49E-49	Fibroblasts
TAGLN2	1.43E-52	0.290668203	0.564	0.326	3.06E-48	Fibroblasts
NDUFS5	8.76E-52	0.312425339	0.684	0.428	1.87E-47	Fibroblasts
NEDD8	1.14E-51	0.257327135	0.497	0.275	2.43E-47	Fibroblasts
INTS6	1.91E-51	0.276600239	0.295	0.137	4.09E-47	Fibroblasts
TLN1	3.81E-51	0.325164266	0.401	0.209	8.14E-47	Fibroblasts
PPP1R12A	3.07E-50	0.333334388	0.336	0.166	6.54E-46	Fibroblasts
DNAJA1	7.94E-50	0.413754194	0.614	0.387	1.70E-45	Fibroblasts
SKP1	1.18E-49	0.361822455	0.65	0.412	2.53E-45	Fibroblasts
TBCA	3.22E-49	0.323558009	0.611	0.375	6.88E-45	Fibroblasts
CXCL2	3.23E-49	0.37756613	0.253	0.116	6.90E-45	Fibroblasts
COPS9	1.32E-48	0.269462701	0.459	0.255	2.82E-44	Fibroblasts
NDUFB9	1.56E-48	0.258608362	0.445	0.238	3.34E-44	Fibroblasts
TMEM219	1.86E-48	0.273411916	0.283	0.135	3.98E-44	Fibroblasts
PRDX2	3.39E-47	0.256468243	0.365	0.188	7.23E-43	Fibroblasts
CCNI	4.33E-47	0.363990739	0.679	0.466	9.23E-43	Fibroblasts
POLR2L	1.33E-46	0.375578942	0.629	0.393	2.84E-42	Fibroblasts
RBBP6	1.60E-46	0.339776188	0.305	0.152	3.41E-42	Fibroblasts
METAP2	2.94E-46	0.293911592	0.489	0.278	6.28E-42	Fibroblasts
HSPA5	3.19E-46	0.463926572	0.383	0.206	6.81E-42	Fibroblasts
ZFP36	3.97E-46	0.67998172	0.62	0.441	8.48E-42	Fibroblasts
NDUFA13	8.49E-46	0.339217755	0.454	0.257	1.81E-41	Fibroblasts
EIF4A2	1.77E-45	0.250885338	0.569	0.342	3.78E-41	Fibroblasts
UBC	2.30E-44	0.551294327	0.87	0.675	4.91E-40	Fibroblasts
ELOB	1.32E-43	0.312221238	0.683	0.439	2.82E-39	Fibroblasts
NFKBIA	2.09E-43	0.40835851	0.674	0.467	4.47E-39	Fibroblasts
ACTB	2.13E-43	0.352305611	0.929	0.806	4.54E-39	Fibroblasts
RHOA	3.34E-43	0.269379488	0.602	0.373	7.13E-39	Fibroblasts
ACTG1	4.77E-39	0.322023821	0.808	0.628	1.02E-34	Fibroblasts
ATF4	2.15E-38	0.304908021	0.585	0.374	4.59E-34	Fibroblasts
CARHSP1	5.55E-38	0.263704469	0.272	0.138	1.18E-33	Fibroblasts
LDHA	2.12E-37	0.294412483	0.641	0.423	4.53E-33	Fibroblasts
BTG2	3.28E-37	0.504614175	0.404	0.247	7.00E-33	Fibroblasts
TRA2B	1.11E-35	0.250506613	0.563	0.363	2.36E-31	Fibroblasts
PPP1CB	3.56E-32	0.321082516	0.448	0.276	7.59E-28	Fibroblasts
MIR4435-2	1.04E-28	0.366027743	0.288	0.164	2.23E-24	Fibroblasts
ZFAND2A	2.30E-26	0.459143817	0.265	0.153	4.90E-22	Fibroblasts
HNRNPH1	8.39E-26	0.278257367	0.454	0.293	1.79E-21	Fibroblasts
S100A41	1.09E-23	0.256872751	0.644	0.484	2.32E-19	Fibroblasts
IRF1	6.60E-21	0.308695246	0.458	0.321	1.41E-16	Fibroblasts

MZB1	0	2.393229041	0.346	0.028	0	Bcell
CD79A	0	2.12722322	0.339	0.012	0	Bcell
JCHAIN	3.84E-239	5.217669089	0.365	0.104	8.20E-235	Bcell
IGHG1	7.32E-209	5.952038717	0.335	0.096	1.56E-204	Bcell
IGKC	3.69E-173	5.979797293	0.623	0.363	7.88E-169	Bcell
IGHM	7.49E-170	4.311830456	0.252	0.06	1.60E-165	Bcell
IGLC2	3.31E-145	6.264334254	0.48	0.248	7.06E-141	Bcell
IGHG3	5.24E-113	4.391413798	0.297	0.118	1.12E-108	Bcell
CD74	3.25E-110	0.8757893	0.699	0.464	6.93E-106	Bcell
FKBP11	4.57E-110	1.108491453	0.294	0.111	9.75E-106	Bcell
HLA-DRA	6.96E-69	0.605584805	0.495	0.308	1.49E-64	Bcell
SEC11C	2.93E-64	0.789127448	0.283	0.137	6.25E-60	Bcell
IGLC3	2.70E-63	5.799397075	0.261	0.127	5.77E-59	Bcell
SSR4	1.64E-59	1.74382059	0.505	0.468	3.50E-55	Bcell
XBP1	8.10E-45	1.040237962	0.311	0.193	1.73E-40	Bcell
IGHA1	4.52E-41	4.809762565	0.388	0.274	9.64E-37	Bcell
HERPUD1	1.14E-38	0.825161353	0.453	0.352	2.44E-34	Bcell
HSPA1A1	4.86E-36	1.157049859	0.441	0.334	1.04E-31	Bcell
HSPA1B1	1.75E-31	1.225085267	0.384	0.287	3.73E-27	Bcell
DNAJB11	3.93E-30	1.458570175	0.547	0.492	8.39E-26	Bcell
RPS4Y1	1.15E-27	0.574742518	0.257	0.154	2.45E-23	Bcell
HLA-DPB1	1.03E-26	0.657094685	0.357	0.261	2.19E-22	Bcell
FKBP21	8.72E-23	0.59233702	0.294	0.211	1.86E-18	Bcell
RPS8	1.79E-18	0.395581629	0.938	0.956	3.82E-14	Bcell
RPS5	8.78E-14	0.33802691	0.82	0.869	1.87E-09	Bcell
CD371	1.06E-13	0.910118383	0.303	0.244	2.26E-09	Bcell
ANKRD28	1.14E-13	0.408440506	0.288	0.216	2.44E-09	Bcell
HSPB11	1.87E-13	0.961040236	0.48	0.438	4.00E-09	Bcell
RPL18A	1.95E-13	0.293174497	0.929	0.959	4.16E-09	Bcell
RPS15A1	1.14E-10	0.261310042	0.976	0.975	2.43E-06	Bcell
SSR31	9.33E-10	0.343537057	0.287	0.232	1.99E-05	Bcell
SMAP21	6.71E-09	0.431826264	0.356	0.293	0.000143	Bcell
ISG201	6.79E-09	0.316821672	0.399	0.332	0.000145	Bcell
SPCS2	2.99E-08	0.334007019	0.304	0.254	0.000639	Bcell
SERP1	5.31E-08	0.435974511	0.475	0.449	0.001134	Bcell
HLA-DPA1	4.28E-07	0.321310263	0.275	0.232	0.009126	Bcell
RPL21	4.76E-07	0.279038444	0.955	0.963	0.01017	Bcell
DNAJA11	5.89E-07	0.448197111	0.335	0.432	0.01257	Bcell
HSPE1	9.05E-07	0.984128621	0.424	0.416	0.019322	Bcell
SOD1	4.72E-06	0.479380324	0.375	0.478	0.100644	Bcell
RPS11	2.33E-05	0.308214247	0.857	0.908	0.498064	Bcell
RPL12	3.58E-05	0.333235951	0.937	0.947	0.76367	Bcell
HSP90AB1	4.80E-05	0.355258575	0.568	0.657	1	Bcell

RPL32	5.02E-05	0.259729038	0.961	0.972	1	Bcell
BTG21	0.000148	0.292898411	0.301	0.262	1	Bcell
HSPD1	0.000775	0.784099484	0.34	0.333	1	Bcell
TRA2B1	0.001497	0.562771985	0.325	0.401	1	Bcell
EEF1B2	0.002948	0.315680595	0.626	0.657	1	Bcell
HNRNPH1	0.00315	0.334681534	0.275	0.322	1	Bcell
CXCL8	0	5.046507348	0.642	0.075	0	Macrophage
G0S2	0	4.54234362	0.602	0.052	0	Macrophage
S100A9	0	4.458540435	0.609	0.047	0	Macrophage
S100A8	0	4.377773179	0.419	0.043	0	Macrophage
SPP1	0	3.976255352	0.295	0.019	0	Macrophage
IL1B	0	3.760337162	0.462	0.032	0	Macrophage
FTL	0	3.749715592	0.994	0.846	0	Macrophage
TYROBP	0	3.721279621	0.936	0.114	0	Macrophage
CCL3	0	3.553640335	0.445	0.073	0	Macrophage
C1QB	0	3.532870861	0.447	0.027	0	Macrophage
APOC1	0	3.431475771	0.407	0.041	0	Macrophage
HLA-DRA1	0	3.412388512	0.88	0.29	0	Macrophage
FCER1G	0	3.398447618	0.815	0.059	0	Macrophage
C1QA	0	3.326997171	0.456	0.025	0	Macrophage
LYZ	0	3.309224055	0.84	0.152	0	Macrophage
CTSB	0	3.185381858	0.734	0.195	0	Macrophage
AIF1	0	3.156871568	0.704	0.017	0	Macrophage
HLA-DPB1	0	2.850725287	0.766	0.233	0	Macrophage
PLAUR	0	2.843969786	0.715	0.106	0	Macrophage
HLA-DPA1	0	2.833082989	0.752	0.194	0	Macrophage
CXCL21	0	2.817834627	0.525	0.1	0	Macrophage
FTH1	0	2.743520885	0.997	0.907	0	Macrophage
EREG	0	2.723988515	0.355	0.02	0	Macrophage
CD741	0	2.717068237	0.889	0.466	0	Macrophage
C15orf48	0	2.709361164	0.617	0.087	0	Macrophage
HLA-DRB1	0	2.669278755	0.786	0.238	0	Macrophage
PSAP1	0	2.620978702	0.789	0.281	0	Macrophage
LST1	0	2.569715085	0.658	0.027	0	Macrophage
C1QC	0	2.492198105	0.388	0.008	0	Macrophage
FCGR2A	0	2.446810935	0.549	0.016	0	Macrophage
SOD21	0	2.441458622	0.722	0.181	0	Macrophage
IL1RN	0	2.312075777	0.307	0.025	0	Macrophage
CTSZ	0	2.309544953	0.584	0.114	0	Macrophage
C5AR1	0	2.291031944	0.49	0.012	0	Macrophage
MS4A7	0	2.28343428	0.505	0.008	0	Macrophage
CD68	0	2.245413952	0.524	0.038	0	Macrophage
CTSS	0	2.230096942	0.691	0.137	0	Macrophage

SAT1	0	2.218990528	0.977	0.637	0	Macrophage
BCL2A1	0	2.213320242	0.475	0.033	0	Macrophage
AC020656.	0	2.19124979	0.512	0.088	0	Macrophage
CTSL1	0	2.165256192	0.498	0.094	0	Macrophage
HLA-DQB1	0	2.156153776	0.577	0.095	0	Macrophage
GLUL1	0	2.152107621	0.7	0.175	0	Macrophage
HLA-DQA1	0	2.13285064	0.536	0.064	0	Macrophage
PLEK	0	2.130559578	0.458	0.028	0	Macrophage
BASP1	0	2.128936119	0.523	0.069	0	Macrophage
NPC21	0	2.114045208	0.728	0.236	0	Macrophage
HLA-DRB5	0	1.964845314	0.586	0.095	0	Macrophage
MS4A6A	0	1.911818335	0.391	0.005	0	Macrophage
OLR1	0	1.857482012	0.337	0.007	0	Macrophage
GRN1	0	1.800890286	0.573	0.125	0	Macrophage
PPIF	0	1.779650807	0.436	0.069	0	Macrophage
IFI30	0	1.779436717	0.437	0.019	0	Macrophage
SLC11A1	0	1.755986357	0.367	0.012	0	Macrophage
IGSF6	0	1.722870903	0.398	0.006	0	Macrophage
ASAH1	0	1.685236855	0.535	0.105	0	Macrophage
CYBB	0	1.663720139	0.401	0.008	0	Macrophage
CD14	0	1.638907902	0.321	0.011	0	Macrophage
MAFB1	0	1.599991492	0.415	0.053	0	Macrophage
HLA-DMA	0	1.530018937	0.524	0.094	0	Macrophage
SMIM25	0	1.512973297	0.267	0.003	0	Macrophage
TGFBI1	0	1.476178234	0.406	0.053	0	Macrophage
SDS	0	1.462834268	0.257	0.003	0	Macrophage
CXCL16	0	1.450906764	0.443	0.04	0	Macrophage
C1orf162	0	1.42834369	0.365	0.022	0	Macrophage
SPI1	0	1.421190278	0.396	0.008	0	Macrophage
GRINA	0	1.408288302	0.486	0.087	0	Macrophage
PLA2G7	0	1.383103962	0.287	0.002	0	Macrophage
SLC16A10	0	1.334193786	0.311	0.006	0	Macrophage
LILRB4	0	1.323952655	0.34	0.004	0	Macrophage
CSTA	0	1.309959903	0.282	0.007	0	Macrophage
RAB311	0	1.299417857	0.47	0.073	0	Macrophage
LGALS2	0	1.28200083	0.307	0.027	0	Macrophage
CLEC7A	0	1.278829919	0.32	0.008	0	Macrophage
CD83	0	1.26649316	0.508	0.088	0	Macrophage
CTSH	0	1.264651424	0.455	0.074	0	Macrophage
FBP1	0	1.246876927	0.254	0.015	0	Macrophage
ITGAX	0	1.24271384	0.301	0.014	0	Macrophage
RNF130	0	1.241579329	0.41	0.049	0	Macrophage
SERPINA1	0	1.227432155	0.438	0.066	0	Macrophage

CSF2RA	0	1.210285486	0.278	0.003	0	Macrophage
SNX10	0	1.187736854	0.328	0.032	0	Macrophage
RNF144B	0	1.142383639	0.278	0.012	0	Macrophage
SLC43A2	0	1.12201528	0.299	0.019	0	Macrophage
CD86	0	1.099721008	0.308	0.005	0	Macrophage
GM2A	0	1.084815326	0.295	0.021	0	Macrophage
HLA-DMB	0	1.077640131	0.338	0.03	0	Macrophage
NCF2	0	1.003353473	0.274	0.004	0	Macrophage
KYNU	0	1.000563644	0.277	0.007	0	Macrophage
SLC31A2	0	0.776683672	0.27	0.016	0	Macrophage
LAIR1	0	0.750996606	0.255	0.017	0	Macrophage
APOE	8.60E-299	3.469023993	0.386	0.058	1.84E-294	Macrophage
MXD1	5.75E-297	1.537202874	0.477	0.086	1.23E-292	Macrophage
RASSF4	1.29E-291	0.860936597	0.267	0.023	2.75E-287	Macrophage
TNFSF13B	5.53E-279	1.031276262	0.321	0.038	1.18E-274	Macrophage
NAMPT	1.00E-275	1.885901813	0.698	0.217	2.14E-271	Macrophage
CST31	2.20E-269	1.737860793	0.811	0.302	4.69E-265	Macrophage
CREG1	2.97E-269	1.288822439	0.395	0.066	6.34E-265	Macrophage
CXCL3	9.26E-269	2.669011512	0.416	0.075	1.98E-264	Macrophage
BRI31	1.71E-267	1.608086979	0.626	0.182	3.65E-263	Macrophage
FAM49A	2.34E-266	0.826038142	0.292	0.032	4.99E-262	Macrophage
OGFRL1	2.39E-259	0.812923179	0.286	0.032	5.10E-255	Macrophage
FGL2	7.47E-259	1.203929863	0.326	0.043	1.60E-254	Macrophage
ACSL1	2.69E-257	1.136805413	0.25	0.024	5.75E-253	Macrophage
HBEGF	3.25E-256	1.36425817	0.386	0.065	6.95E-252	Macrophage
IER31	3.36E-255	1.863485532	0.712	0.236	7.18E-251	Macrophage
RBM47	6.05E-253	0.852583489	0.3	0.037	1.29E-248	Macrophage
ALCAM	1.23E-249	0.832451068	0.287	0.034	2.62E-245	Macrophage
LAPTM5	1.69E-249	1.539404418	0.762	0.278	3.61E-245	Macrophage
GPNMB1	1.23E-246	1.598561892	0.329	0.049	2.63E-242	Macrophage
LIPA	1.70E-245	1.201814694	0.284	0.035	3.63E-241	Macrophage
ACP5	2.95E-245	1.521074034	0.382	0.065	6.30E-241	Macrophage
CTSD	3.84E-242	2.618445063	0.621	0.213	8.19E-238	Macrophage
MGAT1	1.09E-241	1.230351663	0.496	0.113	2.32E-237	Macrophage
VEGFA	3.68E-238	1.366385154	0.405	0.076	7.86E-234	Macrophage
NINJ1	7.83E-238	1.272855518	0.441	0.09	1.67E-233	Macrophage
TNFAIP21	1.01E-236	1.390454414	0.433	0.089	2.15E-232	Macrophage
LYN	4.86E-234	0.821461254	0.353	0.056	1.04E-229	Macrophage
CSTB	1.05E-231	2.200023345	0.758	0.332	2.25E-227	Macrophage
PHACTR1	5.23E-230	1.274072106	0.254	0.028	1.12E-225	Macrophage
TYMP	5.84E-230	1.510036684	0.637	0.206	1.25E-225	Macrophage
TPP1	3.70E-228	0.949872788	0.435	0.088	7.89E-224	Macrophage
ABCA1	9.81E-225	0.822757536	0.272	0.034	2.09E-220	Macrophage

FNIP2	5.50E-224	1.160492997	0.381	0.072	1.17E-219	Macrophage
FCGR3A	4.85E-221	1.032818907	0.317	0.046	1.03E-216	Macrophage
GK	9.67E-217	1.124678005	0.338	0.055	2.06E-212	Macrophage
ITGB2	5.95E-215	1.049324828	0.442	0.093	1.27E-210	Macrophage
MFSD1	4.58E-213	0.847674165	0.283	0.04	9.78E-209	Macrophage
CAPG	1.36E-210	1.437678383	0.455	0.11	2.90E-206	Macrophage
HMOX1	2.56E-209	1.274376098	0.274	0.038	5.46E-205	Macrophage
ATP6V1B2	6.27E-209	0.902153993	0.277	0.039	1.34E-204	Macrophage
TMSB10	7.58E-209	1.046859458	0.987	0.921	1.62E-204	Macrophage
PTGS2	7.08E-205	1.794176447	0.266	0.037	1.51E-200	Macrophage
PPT1	1.78E-204	0.940739624	0.336	0.059	3.81E-200	Macrophage
LGMN	1.81E-204	1.208318767	0.301	0.05	3.86E-200	Macrophage
ARRB2	3.23E-203	0.814198257	0.303	0.047	6.90E-199	Macrophage
FCGRT1	5.44E-203	1.258117923	0.454	0.114	1.16E-198	Macrophage
GPR183	1.15E-202	1.305011468	0.495	0.125	2.45E-198	Macrophage
SGK1	1.21E-200	1.313057842	0.414	0.093	2.58E-196	Macrophage
TMEM176I	9.12E-192	1.139357199	0.383	0.082	1.95E-187	Macrophage
CD4	2.33E-191	0.674356204	0.292	0.045	4.97E-187	Macrophage
CCDC88A	2.99E-191	0.8364151	0.327	0.059	6.38E-187	Macrophage
HNMT	6.08E-191	0.783340113	0.25	0.035	1.30E-186	Macrophage
PLIN2	8.55E-191	1.570498241	0.502	0.139	1.82E-186	Macrophage
CEBPB1	1.46E-189	1.478748742	0.728	0.295	3.11E-185	Macrophage
ATP13A3	4.09E-189	1.046378745	0.37	0.078	8.73E-185	Macrophage
GNS	5.41E-188	0.781457003	0.271	0.042	1.15E-183	Macrophage
S100A111	5.83E-186	1.44730348	0.852	0.485	1.24E-181	Macrophage
CCL3L1	2.71E-178	3.951986759	0.281	0.048	5.79E-174	Macrophage
ETS2	2.86E-178	1.20809212	0.464	0.125	6.10E-174	Macrophage
MARCKS1	3.29E-176	1.196029395	0.483	0.137	7.02E-172	Macrophage
TMEM176/	2.19E-174	1.091859088	0.372	0.084	4.67E-170	Macrophage
TFRC	8.29E-172	1.225861314	0.418	0.107	1.77E-167	Macrophage
LY96	2.93E-171	0.905201463	0.302	0.057	6.26E-167	Macrophage
CCL20	1.74E-170	2.249408126	0.34	0.074	3.71E-166	Macrophage
GNA13	2.61E-169	0.84195259	0.359	0.077	5.56E-165	Macrophage
BID	6.33E-169	0.950099953	0.326	0.067	1.35E-164	Macrophage
IFNGR2	1.22E-167	0.892875613	0.324	0.067	2.61E-163	Macrophage
ATP6V1F	4.63E-167	1.264955442	0.57	0.209	9.89E-163	Macrophage
LCP2	3.78E-163	0.932225808	0.288	0.052	8.08E-159	Macrophage
SLC16A3	2.76E-161	0.889155091	0.391	0.096	5.90E-157	Macrophage
PGD	9.03E-161	0.729933124	0.257	0.043	1.93E-156	Macrophage
ACTB1	1.99E-159	1.029092596	0.953	0.811	4.24E-155	Macrophage
LIMS1	9.67E-159	1.126266213	0.536	0.181	2.06E-154	Macrophage
PDE4DIP	1.82E-157	0.680292326	0.267	0.047	3.88E-153	Macrophage
KCTD12	3.89E-156	0.891576165	0.286	0.056	8.30E-152	Macrophage

LGALS9	2.74E-154	0.735319989	0.303	0.062	5.85E-150	Macrophage
ATP6V0B	1.88E-152	1.069805344	0.531	0.184	4.02E-148	Macrophage
PYCARD	3.54E-152	0.93425317	0.383	0.099	7.56E-148	Macrophage
UPP1	4.43E-151	1.042045374	0.454	0.132	9.45E-147	Macrophage
LITAF	1.02E-149	1.222449164	0.659	0.273	2.18E-145	Macrophage
GPX41	7.51E-149	1.18912685	0.693	0.322	1.60E-144	Macrophage
HIF1A1	1.23E-145	1.160538544	0.479	0.153	2.63E-141	Macrophage
ICAM1	6.27E-143	0.951155746	0.35	0.086	1.34E-138	Macrophage
LCP1	1.62E-142	0.770990909	0.515	0.157	3.46E-138	Macrophage
LGALS11	1.53E-141	0.912030825	0.837	0.46	3.26E-137	Macrophage
H2AFY	2.59E-140	1.01256371	0.511	0.179	5.54E-136	Macrophage
PLD31	3.00E-140	1.115832473	0.316	0.077	6.40E-136	Macrophage
RNF13	6.56E-139	0.828719177	0.32	0.075	1.40E-134	Macrophage
STXBP2	2.91E-138	0.834991927	0.266	0.052	6.21E-134	Macrophage
ABL21	7.86E-137	0.894100762	0.396	0.112	1.68E-132	Macrophage
ATP6AP1	3.81E-134	0.692365273	0.297	0.067	8.13E-130	Macrophage
ATOX11	2.96E-131	1.047660703	0.525	0.197	6.33E-127	Macrophage
VAMP8	4.33E-131	0.993829178	0.533	0.195	9.25E-127	Macrophage
ARHGAP18	2.15E-130	0.716750078	0.327	0.081	4.58E-126	Macrophage
COTL11	5.07E-130	0.985581384	0.654	0.279	1.08E-125	Macrophage
STX11	4.90E-129	0.628111675	0.259	0.052	1.05E-124	Macrophage
RGS10	2.45E-126	0.824127604	0.544	0.196	5.23E-122	Macrophage
APLP21	6.75E-126	1.058955139	0.513	0.19	1.44E-121	Macrophage
AP2S11	1.72E-125	1.002262235	0.53	0.207	3.68E-121	Macrophage
GSTO1	5.27E-125	0.974795505	0.478	0.169	1.13E-120	Macrophage
CMTM6	5.40E-125	0.774395918	0.496	0.17	1.15E-120	Macrophage
QKI	2.54E-124	0.66492339	0.345	0.092	5.42E-120	Macrophage
ATP6V0D1	3.40E-124	0.711228234	0.384	0.111	7.26E-120	Macrophage
GRB2	6.72E-124	0.724696425	0.434	0.138	1.43E-119	Macrophage
ANXA51	1.49E-123	0.907919504	0.534	0.203	3.17E-119	Macrophage
SERPINB1	1.75E-122	0.888238381	0.468	0.159	3.74E-118	Macrophage
ZFYVE16	2.61E-120	0.644323804	0.251	0.053	5.57E-116	Macrophage
ALDH2	1.87E-118	0.727411993	0.275	0.065	4.00E-114	Macrophage
GLIPR2	5.99E-118	0.602112844	0.296	0.072	1.28E-113	Macrophage
RNASE1	1.14E-117	1.521884877	0.271	0.066	2.43E-113	Macrophage
EFHD2	4.21E-114	0.741710907	0.462	0.159	8.98E-110	Macrophage
DAB21	1.09E-113	0.906178867	0.34	0.099	2.34E-109	Macrophage
RNASET2	1.11E-113	0.891111695	0.485	0.177	2.36E-109	Macrophage
NFKB1	1.41E-113	0.853795008	0.456	0.158	3.02E-109	Macrophage
SYNGR2	1.51E-112	0.665330004	0.364	0.108	3.21E-108	Macrophage
CORO1C	7.90E-111	0.593741659	0.262	0.062	1.69E-106	Macrophage
INSIG1	2.42E-110	0.980451057	0.403	0.134	5.17E-106	Macrophage
PTPRE	9.09E-110	0.891589143	0.358	0.107	1.94E-105	Macrophage

ARL8B	4.19E-109	0.544166716	0.261	0.061	8.95E-105	Macrophage
TIMP11	6.13E-109	1.354327305	0.639	0.292	1.31E-104	Macrophage
GNG5	6.39E-107	0.876177474	0.61	0.272	1.36E-102	Macrophage
NFKBIA1	4.82E-106	1.211190641	0.769	0.47	1.03E-101	Macrophage
SPINT2	3.38E-105	0.552958545	0.312	0.084	7.21E-101	Macrophage
SERPINB9	1.43E-104	0.763918528	0.371	0.115	3.04E-100	Macrophage
AP1S2	1.56E-104	0.792183913	0.343	0.104	3.33E-100	Macrophage
SRGN1	5.11E-102	0.958062757	0.922	0.595	1.09E-97	Macrophage
RILPL2	1.22E-101	0.858171156	0.408	0.139	2.60E-97	Macrophage
CYBA	1.39E-101	1.022045926	0.785	0.498	2.96E-97	Macrophage
OAZ1	2.40E-101	0.778917663	0.87	0.682	5.13E-97	Macrophage
PEA15	2.81E-100	0.541565786	0.258	0.065	6.01E-96	Macrophage
S100A101	3.59E-100	0.892616327	0.825	0.532	7.66E-96	Macrophage
SERF2	9.63E-100	0.723045127	0.902	0.752	2.06E-95	Macrophage
NEAT11	1.67E-99	0.798111136	0.958	0.838	3.57E-95	Macrophage
M6PR	5.54E-98	0.476728211	0.269	0.069	1.18E-93	Macrophage
GNAI2	2.65E-96	0.746238627	0.533	0.224	5.65E-92	Macrophage
THBS11	6.18E-95	2.076528117	0.357	0.129	1.32E-90	Macrophage
RAC11	3.73E-92	0.814629442	0.724	0.396	7.96E-88	Macrophage
LGALS31	9.83E-92	0.984075626	0.603	0.294	2.10E-87	Macrophage
FABP5	1.20E-91	1.071149574	0.412	0.151	2.57E-87	Macrophage
PLEKHO1	3.19E-91	0.452183346	0.289	0.082	6.81E-87	Macrophage
CTSC1	1.24E-90	0.863969002	0.439	0.171	2.65E-86	Macrophage
ARPC3	1.84E-88	0.831675618	0.703	0.402	3.92E-84	Macrophage
LAMP2	9.91E-88	0.693151685	0.284	0.086	2.12E-83	Macrophage
GPSM3	1.80E-86	0.498159664	0.348	0.112	3.84E-82	Macrophage
SH3BP5	2.73E-86	0.680355713	0.271	0.079	5.83E-82	Macrophage
SDCBP	3.35E-86	0.787305308	0.616	0.298	7.16E-82	Macrophage
PMP221	5.39E-86	0.645250121	0.329	0.107	1.15E-81	Macrophage
ARPC5	1.06E-85	0.756384018	0.532	0.236	2.26E-81	Macrophage
NR4A3	1.43E-85	0.638490637	0.315	0.099	3.05E-81	Macrophage
HEXB1	3.16E-85	0.714866853	0.288	0.09	6.75E-81	Macrophage
MYO9B	5.22E-85	0.473901011	0.27	0.077	1.11E-80	Macrophage
TXN	1.09E-84	1.158892202	0.697	0.388	2.34E-80	Macrophage
RALA	1.13E-84	0.71797643	0.353	0.126	2.41E-80	Macrophage
CFLAR	4.21E-83	0.741380494	0.585	0.279	8.98E-79	Macrophage
MAP3K8	7.98E-83	0.618554655	0.314	0.102	1.70E-78	Macrophage
BLVRB	1.52E-82	0.813506223	0.29	0.093	3.24E-78	Macrophage
PLSCR1	7.90E-82	0.612741256	0.276	0.085	1.69E-77	Macrophage
GPCPD1	1.41E-81	0.613184091	0.29	0.089	3.00E-77	Macrophage
ATP6AP21	2.46E-81	0.605894658	0.35	0.124	5.25E-77	Macrophage
PKM1	7.88E-80	0.662640663	0.538	0.247	1.68E-75	Macrophage
RHOA1	8.56E-80	0.765505998	0.684	0.379	1.83E-75	Macrophage

TALDO1	6.89E-79	0.606016347	0.365	0.136	1.47E-74	Macrophage
AREG	8.32E-79	1.073538885	0.446	0.193	1.78E-74	Macrophage
CEBPD1	7.14E-78	0.685094655	0.452	0.189	1.52E-73	Macrophage
SKAP2	7.60E-78	0.485899909	0.269	0.082	1.62E-73	Macrophage
FNDC3B1	1.03E-77	0.791850205	0.285	0.094	2.19E-73	Macrophage
ZYX	4.22E-77	0.584055249	0.314	0.107	9.01E-73	Macrophage
ZEB2	5.32E-77	0.436990981	0.502	0.208	1.14E-72	Macrophage
SH3BGRL3	2.47E-76	0.768096412	0.829	0.6	5.28E-72	Macrophage
METRNL	3.06E-76	0.491200917	0.337	0.115	6.54E-72	Macrophage
CTSA	3.24E-76	0.680404072	0.305	0.105	6.92E-72	Macrophage
KLF41	7.12E-76	0.641866253	0.348	0.126	1.52E-71	Macrophage
PDXK	1.32E-75	0.550521938	0.268	0.084	2.81E-71	Macrophage
PLEKHB2	1.42E-75	0.505196063	0.335	0.117	3.04E-71	Macrophage
PFKFB3	1.48E-74	0.694575418	0.288	0.094	3.16E-70	Macrophage
IER5	2.07E-74	0.867860118	0.355	0.133	4.42E-70	Macrophage
FLOT11	9.06E-74	0.688277918	0.329	0.12	1.93E-69	Macrophage
LAMP11	9.30E-74	0.756755793	0.403	0.17	1.98E-69	Macrophage
DBI	1.08E-72	0.714575424	0.621	0.332	2.31E-68	Macrophage
CD631	1.30E-72	0.827207049	0.68	0.384	2.77E-68	Macrophage
GAPDH	1.62E-71	0.639051213	0.924	0.753	3.45E-67	Macrophage
VIM1	1.70E-71	0.819892148	0.826	0.583	3.63E-67	Macrophage
CHMP4B1	2.56E-71	0.620086081	0.451	0.195	5.46E-67	Macrophage
BNIP3L1	2.79E-71	0.762766576	0.342	0.131	5.95E-67	Macrophage
GNAQ	3.08E-71	0.513805139	0.274	0.09	6.58E-67	Macrophage
MOB1A	3.72E-71	0.48165846	0.364	0.137	7.95E-67	Macrophage
JARID2	4.58E-71	0.490559032	0.266	0.084	9.77E-67	Macrophage
H3F3A	4.78E-71	0.717431434	0.89	0.697	1.02E-66	Macrophage
YBX1	1.75E-68	0.758339043	0.78	0.587	3.72E-64	Macrophage
CAP1	1.31E-67	0.653581257	0.488	0.226	2.80E-63	Macrophage
LAMTOR2	3.29E-67	0.563508552	0.34	0.131	7.03E-63	Macrophage
PNRC1	1.66E-66	0.925599073	0.738	0.497	3.53E-62	Macrophage
ANXA21	2.03E-65	0.596970599	0.688	0.388	4.32E-61	Macrophage
STAT11	2.88E-65	0.607079054	0.331	0.128	6.15E-61	Macrophage
SLC2A3	8.44E-65	0.896682408	0.471	0.221	1.80E-60	Macrophage
HCLS1	1.18E-64	0.425298881	0.31	0.109	2.52E-60	Macrophage
SMS	2.78E-64	0.441910174	0.316	0.117	5.94E-60	Macrophage
ENO1	4.07E-64	0.638805999	0.594	0.315	8.69E-60	Macrophage
CHMP1B	4.49E-64	0.660033816	0.447	0.204	9.59E-60	Macrophage
RHOG	4.77E-64	0.476005073	0.367	0.145	1.02E-59	Macrophage
FAM49B	8.23E-64	0.427131731	0.423	0.175	1.76E-59	Macrophage
ARPC1B	2.26E-63	0.633184207	0.59	0.32	4.82E-59	Macrophage
SLC25A37	2.76E-63	1.056197848	0.26	0.094	5.88E-59	Macrophage
CD811	3.04E-63	0.579069156	0.417	0.186	6.50E-59	Macrophage

EMP31	7.66E-63	0.739599463	0.507	0.248	1.63E-58	Macrophage
KDM6B	9.73E-63	0.531608016	0.463	0.211	2.08E-58	Macrophage
IFNGR1	9.91E-63	0.453931653	0.265	0.09	2.12E-58	Macrophage
COMT	1.53E-62	0.523586205	0.267	0.094	3.27E-58	Macrophage
ATG3	1.81E-62	0.496513413	0.259	0.088	3.86E-58	Macrophage
ACTR2	4.86E-62	0.495483652	0.476	0.222	1.04E-57	Macrophage
NOP10	5.00E-62	0.702225694	0.508	0.255	1.07E-57	Macrophage
RAB7A1	2.12E-61	0.560062073	0.373	0.157	4.52E-57	Macrophage
LAMTOR11	6.14E-61	0.479861278	0.357	0.146	1.31E-56	Macrophage
ALOX5AP	8.75E-61	0.695901267	0.378	0.158	1.87E-56	Macrophage
DUSP11	1.17E-59	0.6717239	0.686	0.397	2.50E-55	Macrophage
TNFRSF1B1	2.31E-59	0.451194913	0.434	0.185	4.94E-55	Macrophage
RAB131	6.67E-59	0.539790022	0.316	0.126	1.42E-54	Macrophage
PTPN1	3.49E-58	0.374550518	0.376	0.156	7.46E-54	Macrophage
BLOC1S1	9.09E-58	0.426491507	0.485	0.23	1.94E-53	Macrophage
CDKN1A1	1.19E-57	0.662209468	0.47	0.232	2.54E-53	Macrophage
TPM41	1.20E-57	0.63826796	0.578	0.318	2.56E-53	Macrophage
CCL4L2	1.43E-57	1.678851181	0.274	0.099	3.04E-53	Macrophage
SUMO3	1.90E-57	0.438915148	0.343	0.142	4.06E-53	Macrophage
GSTP11	2.45E-57	0.553806833	0.64	0.349	5.23E-53	Macrophage
PFN1	1.80E-56	0.547239769	0.799	0.586	3.83E-52	Macrophage
ATP5F1E	4.17E-56	0.549740138	0.925	0.794	8.89E-52	Macrophage
SFT2D1	6.57E-56	0.444458116	0.288	0.11	1.40E-51	Macrophage
NFKBIZ1	6.92E-56	0.632851854	0.416	0.194	1.48E-51	Macrophage
UCP2	3.88E-55	0.259511906	0.325	0.124	8.29E-51	Macrophage
TPM3	6.40E-55	0.551154667	0.674	0.399	1.37E-50	Macrophage
SAMHD1	2.84E-54	0.32153628	0.351	0.144	6.06E-50	Macrophage
LAP3	3.12E-54	0.52416453	0.289	0.114	6.67E-50	Macrophage
RAB5C	5.85E-54	0.429713625	0.364	0.159	1.25E-49	Macrophage
HMGA1	2.79E-53	0.312319933	0.309	0.121	5.95E-49	Macrophage
MTHFD2	3.90E-53	0.331547352	0.333	0.135	8.33E-49	Macrophage
PCBP1	9.38E-53	0.572771031	0.568	0.312	2.00E-48	Macrophage
LSP1	1.01E-52	0.613271756	0.44	0.209	2.15E-48	Macrophage
YWHAH	2.17E-52	0.669585226	0.403	0.196	4.64E-48	Macrophage
CD441	2.18E-52	0.695511307	0.653	0.399	4.65E-48	Macrophage
WTAP	5.08E-52	0.553771158	0.46	0.227	1.08E-47	Macrophage
IVNS1ABP	1.13E-51	0.690499322	0.354	0.154	2.41E-47	Macrophage
RNF149	1.49E-51	0.407840923	0.353	0.151	3.17E-47	Macrophage
TSPO	2.82E-51	0.625148397	0.515	0.277	6.02E-47	Macrophage
CANX	2.97E-51	0.528120079	0.41	0.195	6.35E-47	Macrophage
SPG21	2.09E-50	0.324646774	0.251	0.093	4.46E-46	Macrophage
TKT	5.93E-50	0.438155395	0.312	0.132	1.26E-45	Macrophage
RAB1A1	6.19E-50	0.466733373	0.365	0.165	1.32E-45	Macrophage

ASAP11	1.52E-49	0.407192933	0.267	0.104	3.24E-45	Macrophage
RTN41	5.78E-49	0.507889093	0.502	0.263	1.23E-44	Macrophage
SOCS31	1.44E-48	0.380876691	0.368	0.164	3.07E-44	Macrophage
GDI21	1.51E-48	0.418614978	0.355	0.162	3.22E-44	Macrophage
ZFAND51	9.68E-48	0.60883999	0.515	0.282	2.07E-43	Macrophage
PHLDA21	1.26E-47	0.419470754	0.36	0.161	2.69E-43	Macrophage
MT2A1	2.05E-47	0.594991586	0.652	0.431	4.39E-43	Macrophage
TPI11	2.16E-47	0.528570872	0.639	0.375	4.62E-43	Macrophage
RHOB1	3.91E-47	0.568587126	0.424	0.21	8.35E-43	Macrophage
RAB10	1.13E-46	0.39710103	0.276	0.114	2.41E-42	Macrophage
RGS2	7.17E-46	0.637473706	0.456	0.237	1.53E-41	Macrophage
TIMP21	1.51E-45	0.3628397	0.311	0.134	3.22E-41	Macrophage
PRR13	1.80E-45	0.332204558	0.427	0.207	3.84E-41	Macrophage
ZC3H12A	5.33E-45	0.412316399	0.267	0.108	1.14E-40	Macrophage
NR4A2	5.49E-45	0.427370877	0.466	0.239	1.17E-40	Macrophage
SAMSN11	6.91E-45	0.458696428	0.47	0.231	1.47E-40	Macrophage
BCAP31	1.70E-44	0.384312907	0.279	0.118	3.62E-40	Macrophage
CAPZB1	5.52E-44	0.420806298	0.491	0.26	1.18E-39	Macrophage
SNX31	7.39E-44	0.447291008	0.498	0.271	1.58E-39	Macrophage
SPAG9	9.01E-44	0.444728113	0.384	0.187	1.92E-39	Macrophage
TNFRSF14	2.65E-43	0.270971042	0.265	0.107	5.65E-39	Macrophage
YWHAG1	5.30E-43	0.319825107	0.308	0.136	1.13E-38	Macrophage
MT-CO11	3.25E-42	0.323155288	0.974	0.968	6.93E-38	Macrophage
ITM2B1	4.18E-42	0.634862494	0.703	0.474	8.92E-38	Macrophage
HSPA1A2	5.78E-42	0.904907686	0.561	0.332	1.23E-37	Macrophage
ATP1B31	1.68E-41	0.442306808	0.568	0.325	3.59E-37	Macrophage
ATP6V0E1	3.27E-41	0.518521915	0.557	0.329	6.99E-37	Macrophage
HSBP11	5.17E-41	0.404350371	0.36	0.175	1.10E-36	Macrophage
GNB1	6.82E-41	0.281449245	0.376	0.181	1.46E-36	Macrophage
PGK1	8.77E-41	0.446402121	0.477	0.258	1.87E-36	Macrophage
RHEB1	9.59E-41	0.425085847	0.368	0.184	2.05E-36	Macrophage
GNB2	1.52E-40	0.324357437	0.302	0.137	3.25E-36	Macrophage
HSPA6	1.71E-40	0.590666049	0.274	0.119	3.66E-36	Macrophage
CPEB4	4.46E-39	0.317051669	0.282	0.125	9.51E-35	Macrophage
MAP2K3	7.49E-39	0.342728768	0.266	0.115	1.60E-34	Macrophage
PIM3	1.33E-38	0.497706204	0.349	0.171	2.84E-34	Macrophage
COX8A	1.50E-38	0.410971977	0.647	0.401	3.20E-34	Macrophage
NFE2L2	1.78E-38	0.418060857	0.495	0.275	3.80E-34	Macrophage
ETF1	3.20E-38	0.326798483	0.277	0.122	6.84E-34	Macrophage
PPP1R15A	3.63E-38	0.441134342	0.637	0.382	7.75E-34	Macrophage
CDC42	7.11E-38	0.435357518	0.638	0.393	1.52E-33	Macrophage
ARPC21	1.39E-37	0.487911473	0.734	0.554	2.97E-33	Macrophage
ACTR3	1.70E-37	0.402039225	0.374	0.19	3.62E-33	Macrophage

C4orf31	4.91E-37	0.404591855	0.499	0.28	1.05E-32	Macrophage	
CD91	5.25E-37	0.430561172	0.369	0.189	1.12E-32	Macrophage	
ELL2	1.22E-36	0.378677304	0.325	0.154	2.61E-32	Macrophage	
ATF31	1.88E-36	0.293876766	0.383	0.194	4.02E-32	Macrophage	
CLTA1	2.74E-36	0.343909617	0.435	0.232	5.85E-32	Macrophage	
MT-CO3	3.80E-36	0.316519757	0.947	0.925	8.11E-32	Macrophage	
GLRX	4.80E-36	0.299305431	0.304	0.142	1.02E-31	Macrophage	
CFL1	7.90E-36	0.4137722	0.822	0.622	1.69E-31	Macrophage	
BAG31	1.31E-35	1.182956132	0.266	0.129	2.80E-31	Macrophage	
TMBIM6	1.75E-35	0.389834536	0.545	0.323	3.74E-31	Macrophage	
PGLS	3.36E-35	0.271571009	0.282	0.13	7.18E-31	Macrophage	
SSR1	3.76E-35	0.270298586	0.277	0.127	8.03E-31	Macrophage	
MAT2A	4.06E-35	0.337522474	0.291	0.136	8.67E-31	Macrophage	
RNF181	7.51E-35	0.417513382	0.286	0.135	1.60E-30	Macrophage	
CALM3	1.49E-34	0.295387784	0.389	0.205	3.17E-30	Macrophage	
MTPN	2.58E-34	0.30085341	0.4	0.211	5.51E-30	Macrophage	
CAPNS1	3.13E-34	0.34287157	0.3	0.144	6.68E-30	Macrophage	
TUBA1C	5.20E-34	0.348874217	0.315	0.154	1.11E-29	Macrophage	
HSPA1B2	9.66E-34	0.900698839	0.481	0.287	2.06E-29	Macrophage	
B4GALT1	1.56E-33	0.343042934	0.315	0.154	3.33E-29	Macrophage	
WDR1	3.99E-33	0.267132961	0.303	0.147	8.52E-29	Macrophage	
COX6B1	7.33E-33	0.358351105	0.683	0.444	1.57E-28	Macrophage	
CLIC1	1.14E-32	0.371338166	0.627	0.397	2.42E-28	Macrophage	
ZNF706	2.07E-32	0.311097411	0.404	0.224	4.41E-28	Macrophage	
DAZAP2	2.24E-32	0.315784259	0.497	0.285	4.77E-28	Macrophage	
CD164	5.18E-32	0.317786019	0.389	0.209	1.11E-27	Macrophage	
SERPINB61	5.39E-32	0.302384962	0.254	0.118	1.15E-27	Macrophage	
TMEM167/	5.65E-32	0.266564225	0.297	0.144	1.21E-27	Macrophage	
ATP2B1	5.78E-32	0.446675089	0.379	0.206	1.23E-27	Macrophage	
TAPBP	6.51E-32	0.296218609	0.401	0.219	1.39E-27	Macrophage	
PRELID1	8.06E-32	0.368953579	0.469	0.276	1.72E-27	Macrophage	
HSPB12	8.98E-32	0.992951284	0.652	0.426	1.92E-27	Macrophage	
CTNNB11	1.09E-31	0.415658986	0.362	0.193	2.33E-27	Macrophage	
UQCR10	1.24E-31	0.305686693	0.505	0.293	2.65E-27	Macrophage	
FOSL2	1.45E-31	0.283058519	0.42	0.229	3.09E-27	Macrophage	
NDUFB11	3.27E-31	0.377047726	0.536	0.322	6.98E-27	Macrophage	
REL1	3.80E-31	0.287639661	0.649	0.416	8.12E-27	Macrophage	
SH3BGRL1	4.50E-31	0.300565191	0.379	0.201	9.60E-27	Macrophage	
RNH11	7.86E-31	0.314830981	0.292	0.144	1.68E-26	Macrophage	
SPCS3	2.33E-30	0.263520394	0.294	0.146	4.98E-26	Macrophage	
C4orf48	3.76E-30	0.330901208	0.351	0.186	8.03E-26	Macrophage	
LAMTOR4	1.16E-29	0.299988873	0.466	0.27	2.48E-25	Macrophage	
H2AFJ1	2.06E-29	0.271364666	0.283	0.14	4.40E-25	Macrophage	

ATP6V1G1	1.53E-28	0.402480564	0.555	0.357	3.26E-24	Macrophage
PSME2	1.97E-27	0.434381494	0.416	0.243	4.20E-23	Macrophage
EIF4E	3.97E-27	0.355848751	0.271	0.138	8.47E-23	Macrophage
MT-CO2	4.01E-27	0.271444693	0.958	0.949	8.55E-23	Macrophage
SERP11	1.97E-26	0.39007751	0.648	0.436	4.21E-22	Macrophage
UBA52	4.21E-26	0.320824988	0.937	0.883	8.99E-22	Macrophage
TMSB4X1	5.09E-26	0.384372961	0.984	0.983	1.09E-21	Macrophage
SQSTM11	6.64E-26	0.354759688	0.549	0.349	1.42E-21	Macrophage
CCL41	7.78E-26	0.492739726	0.366	0.206	1.66E-21	Macrophage
CKLF	1.12E-25	0.324465636	0.321	0.174	2.38E-21	Macrophage
MAP1LC3B	1.44E-25	0.348070801	0.444	0.26	3.07E-21	Macrophage
POMP	2.61E-25	0.332165359	0.573	0.364	5.57E-21	Macrophage
COX5B	9.31E-25	0.298847272	0.6	0.397	1.99E-20	Macrophage
PABPC4	1.02E-24	0.425569857	0.487	0.304	2.18E-20	Macrophage
PFDN5	1.62E-24	0.334785327	0.844	0.678	3.45E-20	Macrophage
CSNK2B	8.12E-24	0.260029109	0.286	0.153	1.73E-19	Macrophage
TUBA1B1	8.99E-24	0.330477373	0.51	0.322	1.92E-19	Macrophage
IRS2	1.44E-23	0.301812386	0.269	0.141	3.07E-19	Macrophage
GADD45B1	1.74E-22	0.34833776	0.413	0.251	3.71E-18	Macrophage
TMBIM41	2.64E-22	0.278082507	0.312	0.178	5.63E-18	Macrophage
MYL61	2.96E-22	0.353943792	0.828	0.678	6.32E-18	Macrophage
PPA1	2.17E-21	0.444583548	0.346	0.21	4.62E-17	Macrophage
PRDX11	5.40E-21	0.457454513	0.473	0.316	1.15E-16	Macrophage
CALR1	9.18E-21	0.304687112	0.531	0.354	1.96E-16	Macrophage
SEC61B	1.03E-20	0.306275635	0.544	0.362	2.20E-16	Macrophage
ZFP36L11	2.28E-19	0.282312142	0.544	0.362	4.86E-15	Macrophage
TNFAIP31	6.03E-19	0.331808589	0.609	0.431	1.29E-14	Macrophage
CSRNP1	6.97E-19	0.279098647	0.273	0.155	1.49E-14	Macrophage
SEC61G1	8.30E-17	0.256510456	0.505	0.336	1.77E-12	Macrophage
MARCKSL1	6.91E-16	0.284642877	0.251	0.147	1.48E-11	Macrophage
EIF1B	9.52E-16	0.277237415	0.362	0.23	2.03E-11	Macrophage
PSMA7	7.52E-15	0.268368999	0.644	0.468	1.61E-10	Macrophage
DNAJB12	4.61E-14	0.438633741	0.641	0.488	9.83E-10	Macrophage
VAMP51	1.35E-12	0.265333394	0.278	0.178	2.88E-08	Macrophage
CD55	5.56E-11	0.335959362	0.397	0.285	1.19E-06	Macrophage
HSPH1	5.79E-11	0.321239654	0.451	0.332	1.24E-06	Macrophage
SNHG25	4.62E-10	0.280161808	0.397	0.297	9.87E-06	Macrophage
TFF1	0	4.17260699	0.632	0.131	0	adenocarcinoma
TFF3	0	3.721730099	0.64	0.137	0	adenocarcinoma
SPINK1	0	3.473994195	0.605	0.07	0	adenocarcinoma
PHGR1	0	3.319420879	0.607	0.134	0	adenocarcinoma
S100P	0	3.292016908	0.817	0.135	0	adenocarcinoma
KRT19	0	3.281345529	0.814	0.146	0	adenocarcinoma

AGR2	0	3.265430912	0.781	0.14	0	adenocarcinoma
TSPAN8	0	3.16783073	0.721	0.072	0	adenocarcinoma
KRT18	0	3.164201639	0.868	0.173	0	adenocarcinoma
KRT8	0	3.131621134	0.87	0.19	0	adenocarcinoma
FXYD3	0	2.972912061	0.787	0.112	0	adenocarcinoma
LCN2	0	2.710263757	0.573	0.094	0	adenocarcinoma
LGALS4	0	2.672125057	0.693	0.088	0	adenocarcinoma
CD24	0	2.627708908	0.68	0.102	0	adenocarcinoma
C19orf33	0	2.562944831	0.606	0.06	0	adenocarcinoma
IFI271	0	2.518966085	0.889	0.23	0	adenocarcinoma
S100A14	0	2.443043285	0.631	0.082	0	adenocarcinoma
EPCAM	0	2.391555793	0.64	0.064	0	adenocarcinoma
S100A61	0	2.367780604	0.994	0.728	0	adenocarcinoma
GPX2	0	2.343362283	0.443	0.04	0	adenocarcinoma
CYSTM1	0	2.286434354	0.726	0.181	0	adenocarcinoma
ELF3	0	2.275354754	0.6	0.063	0	adenocarcinoma
CLDN4	0	2.218514122	0.603	0.095	0	adenocarcinoma
KRT7	0	2.208008484	0.365	0.032	0	adenocarcinoma
TMC5	0	2.104658232	0.472	0.03	0	adenocarcinoma
ATP1B1	0	2.094894185	0.61	0.1	0	adenocarcinoma
CLDN18	0	1.978859444	0.324	0.022	0	adenocarcinoma
PDZK1IP1	0	1.937870249	0.445	0.029	0	adenocarcinoma
CLDN7	0	1.89982099	0.512	0.045	0	adenocarcinoma
CLDN3	0	1.869056335	0.484	0.037	0	adenocarcinoma
SMIM22	0	1.85527456	0.518	0.058	0	adenocarcinoma
TSPAN1	0	1.851700685	0.442	0.04	0	adenocarcinoma
PRSS3	0	1.774651611	0.458	0.043	0	adenocarcinoma
GPRC5A	0	1.751281019	0.504	0.083	0	adenocarcinoma
MAL2	0	1.648686812	0.432	0.022	0	adenocarcinoma
NQO1	0	1.6105019	0.427	0.039	0	adenocarcinoma
LMO7	0	1.557372021	0.417	0.054	0	adenocarcinoma
LIPH	0	1.548699326	0.32	0.009	0	adenocarcinoma
MUC13	0	1.514828853	0.333	0.032	0	adenocarcinoma
SPINT21	0	1.505257918	0.492	0.071	0	adenocarcinoma
SFN	0	1.492854559	0.384	0.045	0	adenocarcinoma
SDCBP2	0	1.446498354	0.346	0.023	0	adenocarcinoma
AGR3	0	1.424654725	0.281	0.011	0	adenocarcinoma
KLF5	0	1.415914037	0.429	0.042	0	adenocarcinoma
TMEM54	0	1.397459204	0.404	0.047	0	adenocarcinoma
DSP	0	1.39680398	0.376	0.02	0	adenocarcinoma
CYP3A5	0	1.35374553	0.282	0.016	0	adenocarcinoma
CTSE	0	1.332989775	0.259	0.009	0	adenocarcinoma
SLPI	0	1.285479381	0.332	0.032	0	adenocarcinoma

DSG2	0	1.223652894	0.363	0.034	0	adenocarcinoma
MYH14	0	1.15009142	0.298	0.02	0	adenocarcinoma
DSC2	0	1.107219684	0.288	0.023	0	adenocarcinoma
MISP	0	1.059169629	0.326	0.026	0	adenocarcinoma
SPINT1	0	1.038146475	0.3	0.027	0	adenocarcinoma
RAB25	0	1.003599151	0.303	0.012	0	adenocarcinoma
JUP	0	0.974303306	0.3	0.026	0	adenocarcinoma
PLS1	0	0.968844603	0.293	0.018	0	adenocarcinoma
SERPINB5	0	0.920959493	0.251	0.013	0	adenocarcinoma
LAD1	0	0.880822251	0.266	0.014	0	adenocarcinoma
CDH1	0	0.838816848	0.258	0.013	0	adenocarcinoma
TACSTD2	3.99E-307	1.221874274	0.25	0.017	8.52E-303	adenocarcinoma
SLC44A4	2.27E-306	0.988927695	0.291	0.025	4.85E-302	adenocarcinoma
REG4	2.41E-305	2.428359229	0.435	0.069	5.15E-301	adenocarcinoma
PKP3	1.96E-298	0.92313831	0.274	0.022	4.18E-294	adenocarcinoma
PIGR	1.11E-297	2.491945021	0.522	0.109	2.37E-293	adenocarcinoma
PPP1R16A	1.24E-291	0.807800123	0.273	0.023	2.65E-287	adenocarcinoma
MUC1	6.92E-290	1.642132038	0.372	0.049	1.48E-285	adenocarcinoma
PERP	1.31E-288	1.522388312	0.511	0.099	2.80E-284	adenocarcinoma
LAMB3	4.71E-285	1.065656525	0.302	0.03	1.00E-280	adenocarcinoma
SERINC2	1.78E-279	0.946228958	0.281	0.027	3.79E-275	adenocarcinoma
S100A161	3.93E-261	1.508110344	0.514	0.113	8.38E-257	adenocarcinoma
MGST1	1.80E-258	1.194831454	0.371	0.055	3.85E-254	adenocarcinoma
MT1G	3.88E-256	1.70693963	0.328	0.043	8.29E-252	adenocarcinoma
STARD10	8.04E-252	1.190292074	0.335	0.045	1.72E-247	adenocarcinoma
ST14	1.02E-247	0.849796973	0.253	0.024	2.18E-243	adenocarcinoma
TM4SF1	3.45E-237	1.45034088	0.605	0.163	7.36E-233	adenocarcinoma
MDK1	7.45E-229	1.489203184	0.435	0.089	1.59E-224	adenocarcinoma
FHL21	7.96E-228	1.309900666	0.365	0.061	1.70E-223	adenocarcinoma
LSR	7.25E-225	0.914330014	0.265	0.03	1.55E-220	adenocarcinoma
RPL8	7.80E-221	1.205809255	0.971	0.89	1.67E-216	adenocarcinoma
ASS1	2.42E-220	1.121280609	0.323	0.048	5.17E-216	adenocarcinoma
CAMK2N1	3.26E-220	1.183130195	0.382	0.069	6.95E-216	adenocarcinoma
C15orf481	7.63E-212	1.706566038	0.456	0.103	1.63E-207	adenocarcinoma
HMGA11	9.13E-212	1.356889345	0.47	0.109	1.95E-207	adenocarcinoma
SDC4	9.36E-212	1.384665708	0.347	0.058	2.00E-207	adenocarcinoma
SH3YL1	4.31E-211	0.823270759	0.253	0.029	9.19E-207	adenocarcinoma
MYO6	1.22E-207	0.987982131	0.293	0.042	2.59E-203	adenocarcinoma
LYZ1	1.73E-205	2.168602133	0.592	0.176	3.70E-201	adenocarcinoma
TXN1	4.59E-205	1.701730428	0.767	0.384	9.79E-201	adenocarcinoma
CES2	1.16E-200	1.062241467	0.307	0.048	2.49E-196	adenocarcinoma
AGPAT2	2.30E-190	1.141627318	0.355	0.069	4.92E-186	adenocarcinoma
PHLDA22	4.89E-190	1.427781075	0.523	0.149	1.04E-185	adenocarcinoma

KRT20	9.79E-189	1.392413513	0.264	0.038	2.09E-184	adenocarcinoma
RBM471	1.35E-186	0.802009297	0.275	0.041	2.89E-182	adenocarcinoma
GSTP12	1.22E-182	1.634076797	0.719	0.345	2.61E-178	adenocarcinoma
TPD52	3.13E-180	1.031494367	0.352	0.068	6.68E-176	adenocarcinoma
GOLM1	6.90E-179	0.948752237	0.29	0.048	1.47E-174	adenocarcinoma
S100A112	6.54E-176	1.428523123	0.843	0.488	1.40E-171	adenocarcinoma
MT1E1	2.70E-174	1.279712212	0.511	0.147	5.76E-170	adenocarcinoma
ANXA22	3.13E-174	1.536450344	0.752	0.384	6.68E-170	adenocarcinoma
NET1	7.06E-169	0.696040156	0.267	0.042	1.51E-164	adenocarcinoma
LGALS32	1.76E-165	1.620354082	0.67	0.29	3.76E-161	adenocarcinoma
TSTA3	2.75E-163	1.004626376	0.384	0.09	5.88E-159	adenocarcinoma
CHCHD10	5.48E-162	1.274900329	0.541	0.178	1.17E-157	adenocarcinoma
NAPRT	4.11E-161	0.927519475	0.315	0.062	8.78E-157	adenocarcinoma
TPM11	2.85E-160	0.816301387	0.571	0.18	6.08E-156	adenocarcinoma
PGC	2.87E-157	3.083689517	0.341	0.076	6.14E-153	adenocarcinoma
CD2AP	1.02E-153	0.866579641	0.307	0.061	2.17E-149	adenocarcinoma
ACTG11	1.52E-152	1.116907501	0.891	0.633	3.25E-148	adenocarcinoma
CDC42EP5	1.14E-151	0.829298163	0.286	0.054	2.44E-147	adenocarcinoma
PCBD1	3.95E-150	0.881441292	0.361	0.085	8.42E-146	adenocarcinoma
ANXA4	3.58E-148	1.006605678	0.31	0.066	7.65E-144	adenocarcinoma
MPST	3.45E-146	0.877007822	0.325	0.071	7.37E-142	adenocarcinoma
SERPINA11	2.66E-145	1.870448494	0.329	0.076	5.68E-141	adenocarcinoma
ASPH1	5.29E-145	0.965059776	0.372	0.094	1.13E-140	adenocarcinoma
TSPAN3	8.45E-145	0.982334397	0.379	0.097	1.80E-140	adenocarcinoma
ETHE1	1.44E-143	1.01195809	0.41	0.113	3.06E-139	adenocarcinoma
ITGA6	1.83E-143	0.729525914	0.271	0.05	3.90E-139	adenocarcinoma
CST32	7.87E-142	0.852931008	0.758	0.309	1.68E-137	adenocarcinoma
FABP1	4.25E-136	2.483484973	0.359	0.096	9.08E-132	adenocarcinoma
PI3	6.20E-133	1.109857315	0.255	0.049	1.32E-128	adenocarcinoma
REG1A	2.50E-132	2.101144434	0.269	0.056	5.33E-128	adenocarcinoma
PLAC8	2.50E-132	1.033647262	0.316	0.072	5.33E-128	adenocarcinoma
COMTD1	3.75E-132	0.755325172	0.25	0.047	8.01E-128	adenocarcinoma
SERPINB62	2.28E-131	0.950231533	0.387	0.108	4.87E-127	adenocarcinoma
EIF5A1	4.60E-129	1.261557774	0.567	0.226	9.82E-125	adenocarcinoma
BLVRB1	1.66E-128	0.87840785	0.345	0.089	3.55E-124	adenocarcinoma
TMEM141	3.00E-127	0.797776695	0.297	0.067	6.40E-123	adenocarcinoma
RPL36	1.62E-124	0.701931848	0.968	0.92	3.45E-120	adenocarcinoma
LGALS3BP1	3.52E-123	1.013092636	0.377	0.107	7.51E-119	adenocarcinoma
CXCL31	1.01E-118	0.647492857	0.328	0.084	2.17E-114	adenocarcinoma
LIPF	2.75E-118	2.468339526	0.286	0.067	5.86E-114	adenocarcinoma
RPL29	8.22E-117	0.81440132	0.95	0.889	1.76E-112	adenocarcinoma
DSTN1	1.04E-116	0.795745954	0.725	0.365	2.22E-112	adenocarcinoma
SSFA2	8.78E-115	0.625926342	0.259	0.056	1.87E-110	adenocarcinoma

MGST2	4.27E-114	0.822668506	0.286	0.069	9.11E-110	adenocarcinoma
S100A102	5.75E-114	1.25582604	0.785	0.537	1.23E-109	adenocarcinoma
ID1	9.33E-114	1.11833402	0.352	0.1	1.99E-109	adenocarcinoma
FLNB	1.37E-112	0.733830994	0.282	0.067	2.92E-108	adenocarcinoma
MIF1	2.88E-112	1.179608939	0.636	0.311	6.14E-108	adenocarcinoma
BPIFB1	3.42E-111	2.612097268	0.313	0.083	7.30E-107	adenocarcinoma
FDFT1	2.17E-109	0.702158513	0.319	0.084	4.62E-105	adenocarcinoma
CTNND1	3.37E-109	0.700741792	0.254	0.057	7.19E-105	adenocarcinoma
PPDPF	1.65E-108	1.141512846	0.738	0.437	3.52E-104	adenocarcinoma
CTTN1	4.44E-108	0.754176353	0.277	0.067	9.48E-104	adenocarcinoma
JPT1	5.26E-108	1.22322748	0.568	0.247	1.12E-103	adenocarcinoma
EPS81	6.27E-108	0.696757223	0.287	0.071	1.34E-103	adenocarcinoma
C12orf75	1.29E-107	0.757268219	0.386	0.116	2.76E-103	adenocarcinoma
COX6B11	1.55E-107	1.076049504	0.734	0.441	3.31E-103	adenocarcinoma
COX5B1	2.62E-107	1.027910193	0.699	0.39	5.59E-103	adenocarcinoma
RPL121	2.30E-106	0.70108518	0.971	0.943	4.90E-102	adenocarcinoma
TUBA1C1	2.43E-105	0.884985919	0.427	0.146	5.18E-101	adenocarcinoma
TSPO1	3.49E-105	1.097801284	0.585	0.273	7.44E-101	adenocarcinoma
EIF6	3.60E-105	0.920978582	0.381	0.119	7.68E-101	adenocarcinoma
COX6A1	1.74E-104	1.102178162	0.687	0.394	3.71E-100	adenocarcinoma
UQCRQ1	5.34E-104	1.166024378	0.693	0.395	1.14E-99	adenocarcinoma
SERPINB11	5.89E-103	0.953485191	0.446	0.163	1.26E-98	adenocarcinoma
CYC11	2.06E-102	0.858986006	0.385	0.127	4.41E-98	adenocarcinoma
TXNDC17	6.39E-102	0.826642727	0.447	0.161	1.36E-97	adenocarcinoma
RPS191	2.78E-101	0.627767594	0.991	0.973	5.94E-97	adenocarcinoma
ATP5F1D	6.71E-101	1.076859018	0.688	0.37	1.43E-96	adenocarcinoma
PRDX12	2.11E-100	1.113772675	0.626	0.305	4.51E-96	adenocarcinoma
CSTB1	9.47E-100	1.109647917	0.64	0.344	2.02E-95	adenocarcinoma
PPA11	1.16E-99	1.051373746	0.488	0.2	2.47E-95	adenocarcinoma
HEBP2	1.40E-99	0.795592177	0.427	0.152	3.00E-95	adenocarcinoma
CCND1	2.71E-98	0.643453874	0.252	0.06	5.77E-94	adenocarcinoma
ADIRF1	9.32E-98	0.533397542	0.421	0.144	1.99E-93	adenocarcinoma
RPL35	1.28E-97	0.661993728	0.962	0.904	2.72E-93	adenocarcinoma
SLC12A2	1.65E-97	0.736989753	0.25	0.06	3.51E-93	adenocarcinoma
ABHD2	2.50E-97	0.614343251	0.262	0.065	5.34E-93	adenocarcinoma
RBCK1	1.56E-96	0.787494722	0.354	0.111	3.34E-92	adenocarcinoma
RAB11FIP1	1.94E-96	1.014602625	0.472	0.176	4.13E-92	adenocarcinoma
TIMM131	2.84E-96	0.966749056	0.451	0.172	6.06E-92	adenocarcinoma
CLTB1	2.99E-94	0.9837364	0.429	0.16	6.38E-90	adenocarcinoma
RPS51	1.30E-93	0.741726844	0.936	0.856	2.78E-89	adenocarcinoma
IFI61	4.13E-93	1.682107361	0.437	0.181	8.81E-89	adenocarcinoma
ARPC1A	5.78E-93	0.754576127	0.305	0.089	1.23E-88	adenocarcinoma
PLCG2	1.31E-92	2.620284525	0.369	0.123	2.80E-88	adenocarcinoma

ZNF593	5.35E-92	0.703549892	0.311	0.091	1.14E-87	adenocarcinoma
ATP5MC3	3.91E-91	1.005496733	0.593	0.299	8.35E-87	adenocarcinoma
SPTBN11	4.30E-90	0.663038961	0.42	0.148	9.19E-86	adenocarcinoma
CTNNA1	4.38E-90	0.721887888	0.308	0.092	9.36E-86	adenocarcinoma
TMBIM1	3.41E-89	0.656293488	0.291	0.083	7.28E-85	adenocarcinoma
LIMA11	1.13E-88	0.698673653	0.381	0.13	2.42E-84	adenocarcinoma
GAPDH1	1.18E-88	0.880210728	0.884	0.757	2.52E-84	adenocarcinoma
CYB5A1	5.20E-87	0.727091817	0.314	0.097	1.11E-82	adenocarcinoma
TKT1	7.95E-87	0.849144628	0.367	0.128	1.70E-82	adenocarcinoma
CISD3	1.16E-86	0.602548642	0.287	0.083	2.48E-82	adenocarcinoma
VDAC1	2.84E-86	0.916692201	0.5	0.219	6.06E-82	adenocarcinoma
MUC6	3.17E-86	2.1261891	0.264	0.073	6.78E-82	adenocarcinoma
IER32	3.77E-86	0.665984141	0.567	0.251	8.05E-82	adenocarcinoma
PRDX51	1.20E-85	0.9359173	0.54	0.249	2.57E-81	adenocarcinoma
COX5A	1.90E-85	0.89946551	0.534	0.244	4.05E-81	adenocarcinoma
COX6C	1.91E-85	0.988894843	0.687	0.422	4.07E-81	adenocarcinoma
RPS18	4.93E-85	0.61628271	0.99	0.982	1.05E-80	adenocarcinoma
GFPT1	1.00E-84	0.557915534	0.253	0.067	2.14E-80	adenocarcinoma
IFITM31	1.59E-84	0.546584961	0.657	0.294	3.39E-80	adenocarcinoma
SPATS2L1	1.91E-84	0.805409293	0.409	0.151	4.08E-80	adenocarcinoma
CD92	2.48E-84	0.679054564	0.462	0.183	5.29E-80	adenocarcinoma
MGST31	5.04E-84	0.924402349	0.462	0.193	1.08E-79	adenocarcinoma
CD1511	7.73E-84	0.799364884	0.359	0.124	1.65E-79	adenocarcinoma
PDLIM11	9.71E-84	0.558254012	0.402	0.141	2.07E-79	adenocarcinoma
ATP5MF	2.05E-83	0.905924963	0.58	0.287	4.37E-79	adenocarcinoma
RAC12	1.40E-82	1.033807222	0.669	0.403	2.99E-78	adenocarcinoma
ALDH21	3.04E-82	0.602733191	0.251	0.068	6.50E-78	adenocarcinoma
OCIAD2	4.51E-82	0.783843115	0.408	0.152	9.63E-78	adenocarcinoma
DNAJC15	1.41E-80	0.758150662	0.405	0.155	3.00E-76	adenocarcinoma
PLA2G16	1.43E-80	0.817416468	0.292	0.091	3.05E-76	adenocarcinoma
RPL18A1	3.70E-80	0.489088854	0.974	0.953	7.91E-76	adenocarcinoma
TPI12	7.83E-80	0.89103092	0.661	0.374	1.67E-75	adenocarcinoma
SRI	8.64E-80	0.991678391	0.403	0.159	1.84E-75	adenocarcinoma
POLR2H	1.26E-79	0.614124307	0.286	0.086	2.70E-75	adenocarcinoma
RPLP0	4.75E-79	0.743790455	0.868	0.768	1.01E-74	adenocarcinoma
MRPL23	2.00E-78	0.641304304	0.3	0.095	4.27E-74	adenocarcinoma
TM9SF3	4.52E-78	0.760450346	0.41	0.162	9.65E-74	adenocarcinoma
ATP5ME	4.83E-78	0.999445111	0.701	0.452	1.03E-73	adenocarcinoma
CD551	1.64E-77	1.003314024	0.556	0.273	3.49E-73	adenocarcinoma
KDELR21	2.65E-77	0.822681131	0.46	0.195	5.65E-73	adenocarcinoma
PRR131	6.77E-77	0.758262789	0.475	0.204	1.44E-72	adenocarcinoma
HMGCS1	1.02E-76	0.612567407	0.265	0.078	2.17E-72	adenocarcinoma
ATP5MC11	3.07E-76	0.87193517	0.416	0.17	6.55E-72	adenocarcinoma

AC020656.	5.13E-76	1.770148077	0.31	0.106	1.10E-71	adenocarcinoma
NDUFB91	6.26E-76	0.831174598	0.515	0.246	1.34E-71	adenocarcinoma
STOML2	1.35E-75	0.718922477	0.328	0.114	2.87E-71	adenocarcinoma
PRDX21	3.75E-75	0.880741114	0.448	0.192	8.01E-71	adenocarcinoma
ECHS1	6.13E-74	0.601938347	0.316	0.108	1.31E-69	adenocarcinoma
RPL22L1	3.68E-73	0.970781241	0.556	0.297	7.85E-69	adenocarcinoma
RPL37A	4.07E-73	0.562707667	0.966	0.939	8.70E-69	adenocarcinoma
DDT	5.42E-73	0.803886694	0.478	0.218	1.16E-68	adenocarcinoma
SLIRP	2.96E-72	0.78927774	0.443	0.19	6.31E-68	adenocarcinoma
P4HB1	4.33E-72	0.863817456	0.531	0.255	9.23E-68	adenocarcinoma
MRPL12	9.24E-72	0.616561849	0.254	0.077	1.97E-67	adenocarcinoma
ELOB1	1.14E-71	0.848221964	0.715	0.452	2.43E-67	adenocarcinoma
ATF32	2.65E-70	0.874810474	0.442	0.191	5.65E-66	adenocarcinoma
MDH2	3.41E-70	0.656991176	0.365	0.139	7.29E-66	adenocarcinoma
LSM4	3.74E-70	0.646139961	0.35	0.128	7.98E-66	adenocarcinoma
UQCR101	1.86E-69	0.823713943	0.563	0.29	3.96E-65	adenocarcinoma
GPI	6.42E-69	0.601275479	0.308	0.107	1.37E-64	adenocarcinoma
RPL41	2.02E-67	0.489561307	0.987	0.993	4.30E-63	adenocarcinoma
TMSB101	7.36E-66	0.578629806	0.959	0.924	1.57E-61	adenocarcinoma
ETS21	7.94E-66	0.719413017	0.353	0.136	1.70E-61	adenocarcinoma
RAB11A	8.09E-66	0.612012677	0.353	0.135	1.73E-61	adenocarcinoma
COX7B	3.59E-65	0.874671209	0.609	0.345	7.65E-61	adenocarcinoma
MRPL52	4.82E-65	0.668380038	0.394	0.162	1.03E-60	adenocarcinoma
UQCRC1	9.39E-65	0.595441744	0.293	0.102	2.00E-60	adenocarcinoma
PLEC	9.95E-65	0.685462909	0.319	0.117	2.12E-60	adenocarcinoma
ZNHIT11	1.20E-64	0.719040467	0.464	0.213	2.56E-60	adenocarcinoma
PDIA4	1.22E-64	0.72241288	0.31	0.114	2.60E-60	adenocarcinoma
COX7A2	2.27E-64	0.848964015	0.686	0.476	4.84E-60	adenocarcinoma
UQCRH	3.95E-64	0.881952146	0.648	0.415	8.43E-60	adenocarcinoma
PKM2	4.37E-64	0.866534823	0.506	0.252	9.34E-60	adenocarcinoma
RPL18	5.72E-64	0.596301104	0.939	0.886	1.22E-59	adenocarcinoma
MRPL14	1.93E-63	0.497568772	0.31	0.111	4.12E-59	adenocarcinoma
SLC25A5	2.38E-63	0.87063589	0.595	0.354	5.09E-59	adenocarcinoma
ZFAS1	7.26E-63	1.09274036	0.671	0.452	1.55E-58	adenocarcinoma
ROMO11	9.04E-63	0.775884669	0.521	0.261	1.93E-58	adenocarcinoma
MARCKSL1	1.13E-62	0.641684081	0.356	0.139	2.41E-58	adenocarcinoma
PPP2CB	1.37E-62	0.513289018	0.25	0.08	2.92E-58	adenocarcinoma
LDLR	2.93E-62	0.593061788	0.285	0.098	6.26E-58	adenocarcinoma
SUCLG1	1.30E-61	0.475868455	0.264	0.088	2.77E-57	adenocarcinoma
VDAC2	7.43E-61	0.71881937	0.446	0.213	1.59E-56	adenocarcinoma
NME1	8.60E-60	0.687002629	0.294	0.109	1.84E-55	adenocarcinoma
CHMP51	2.33E-59	0.604934462	0.321	0.125	4.97E-55	adenocarcinoma
AURKAIP1	2.57E-59	0.712928439	0.46	0.214	5.48E-55	adenocarcinoma

POLR2I1	5.32E-59	0.628590747	0.342	0.138	1.14E-54	adenocarcinoma
ATP5MPL	6.36E-59	0.765200056	0.642	0.388	1.36E-54	adenocarcinoma
FAM162A	1.60E-58	0.573227469	0.292	0.107	3.41E-54	adenocarcinoma
LYPLA1	1.71E-58	0.416383491	0.293	0.106	3.65E-54	adenocarcinoma
YWHAE1	2.01E-58	0.674714473	0.566	0.299	4.28E-54	adenocarcinoma
RND31	2.84E-58	0.386767189	0.256	0.086	6.07E-54	adenocarcinoma
PRDX3	3.47E-58	0.481108396	0.261	0.089	7.40E-54	adenocarcinoma
SYPL1	1.44E-57	0.555831745	0.293	0.109	3.07E-53	adenocarcinoma
C1QBP	1.89E-56	0.632523407	0.358	0.152	4.03E-52	adenocarcinoma
HES11	2.15E-56	0.415113689	0.399	0.168	4.59E-52	adenocarcinoma
CLTA2	6.92E-56	0.644440022	0.471	0.23	1.48E-51	adenocarcinoma
NDUFC11	8.94E-56	0.523065989	0.386	0.169	1.91E-51	adenocarcinoma
PHB	1.49E-55	0.642865567	0.291	0.111	3.19E-51	adenocarcinoma
CHMP4B2	2.05E-55	0.641128061	0.425	0.199	4.37E-51	adenocarcinoma
EFHD21	3.57E-55	0.545172013	0.387	0.167	7.61E-51	adenocarcinoma
SMS1	6.23E-55	0.455447967	0.308	0.119	1.33E-50	adenocarcinoma
RPS15	8.73E-55	0.386784634	0.978	0.967	1.86E-50	adenocarcinoma
NDUFS6	9.01E-55	0.792995465	0.464	0.232	1.92E-50	adenocarcinoma
BSG1	1.14E-54	0.589961756	0.46	0.222	2.42E-50	adenocarcinoma
COX7C	1.85E-54	0.639885295	0.788	0.668	3.95E-50	adenocarcinoma
USP53	2.62E-54	0.487412371	0.271	0.098	5.58E-50	adenocarcinoma
CCDC25	4.42E-54	0.481724058	0.264	0.094	9.44E-50	adenocarcinoma
ATP5F1B	4.82E-54	0.657413879	0.465	0.234	1.03E-49	adenocarcinoma
CENPX	1.59E-53	0.534608744	0.261	0.094	3.39E-49	adenocarcinoma
RHOC1	1.84E-53	0.655621788	0.418	0.192	3.92E-49	adenocarcinoma
ATP5MD	2.57E-53	0.758629192	0.581	0.331	5.48E-49	adenocarcinoma
ACADVL	7.43E-53	0.577054632	0.32	0.13	1.59E-48	adenocarcinoma
ATP5IF1	9.18E-53	0.655289323	0.503	0.263	1.96E-48	adenocarcinoma
KDELR11	3.38E-52	0.602749994	0.342	0.147	7.21E-48	adenocarcinoma
WDR45B	5.42E-52	0.40412248	0.306	0.121	1.16E-47	adenocarcinoma
COX4I1	6.86E-52	0.627247708	0.774	0.651	1.46E-47	adenocarcinoma
ERGIC3	1.13E-51	0.690661528	0.394	0.18	2.40E-47	adenocarcinoma
SMIM26	1.49E-51	0.51539611	0.374	0.164	3.18E-47	adenocarcinoma
HINT11	1.61E-50	0.730101344	0.713	0.537	3.44E-46	adenocarcinoma
COX8A1	3.50E-50	0.659719375	0.632	0.403	7.46E-46	adenocarcinoma
SEM11	3.96E-49	0.631353323	0.523	0.285	8.46E-45	adenocarcinoma
NDUFB71	8.26E-49	0.7436173	0.471	0.241	1.76E-44	adenocarcinoma
NUTF2	1.73E-48	0.453992677	0.268	0.102	3.68E-44	adenocarcinoma
CD1641	4.16E-48	0.569538347	0.425	0.207	8.89E-44	adenocarcinoma
RPS21	6.79E-48	0.52551807	0.894	0.845	1.45E-43	adenocarcinoma
RRBP11	8.22E-48	0.702662042	0.518	0.28	1.75E-43	adenocarcinoma
RHOF	1.42E-47	0.434808397	0.271	0.103	3.03E-43	adenocarcinoma
RPL7A	3.57E-47	0.532277413	0.909	0.873	7.62E-43	adenocarcinoma

ETFB	7.79E-47	0.619191653	0.297	0.124	1.66E-42	adenocarcinoma
ACTB2	9.17E-47	0.463452216	0.922	0.814	1.96E-42	adenocarcinoma
TRIB1	1.35E-45	0.489993738	0.284	0.117	2.88E-41	adenocarcinoma
PYCARD1	1.54E-45	0.427804945	0.275	0.109	3.28E-41	adenocarcinoma
PLAUR1	2.14E-45	0.299258365	0.328	0.14	4.57E-41	adenocarcinoma
MT-CO21	2.22E-45	0.413683591	0.955	0.949	4.73E-41	adenocarcinoma
PDCD51	4.14E-45	0.519542419	0.329	0.146	8.84E-41	adenocarcinoma
CHMP2B	4.75E-45	0.381619342	0.291	0.119	1.01E-40	adenocarcinoma
BRI32	5.02E-45	0.479902464	0.412	0.201	1.07E-40	adenocarcinoma
SCP2	1.14E-44	0.509978063	0.329	0.145	2.44E-40	adenocarcinoma
HDGF	1.44E-44	0.424207662	0.268	0.107	3.08E-40	adenocarcinoma
SELENOW1	1.71E-44	0.557729449	0.544	0.309	3.64E-40	adenocarcinoma
RPL37	5.01E-44	0.393754672	0.955	0.941	1.07E-39	adenocarcinoma
ATP5PO	1.14E-43	0.648664134	0.462	0.243	2.44E-39	adenocarcinoma
EDF1	4.11E-43	0.632298854	0.673	0.449	8.78E-39	adenocarcinoma
MPC2	4.12E-43	0.413530017	0.34	0.152	8.79E-39	adenocarcinoma
ZNF7061	5.66E-43	0.537403007	0.434	0.222	1.21E-38	adenocarcinoma
TMBIM61	1.43E-42	0.624248317	0.543	0.324	3.06E-38	adenocarcinoma
STK24	2.18E-42	0.391031402	0.281	0.116	4.66E-38	adenocarcinoma
CHMP2A1	3.29E-42	0.64635241	0.374	0.184	7.02E-38	adenocarcinoma
SEC61G2	8.58E-42	0.707704346	0.543	0.334	1.83E-37	adenocarcinoma
MRPL41	9.36E-42	0.605595166	0.397	0.198	2.00E-37	adenocarcinoma
ISG15	1.24E-41	1.077481191	0.47	0.268	2.64E-37	adenocarcinoma
CLIC11	1.68E-41	0.640143077	0.616	0.399	3.59E-37	adenocarcinoma
VAMP81	1.71E-41	0.479798108	0.42	0.206	3.66E-37	adenocarcinoma
KLF42	2.13E-41	0.726323334	0.297	0.131	4.55E-37	adenocarcinoma
MXD11	3.33E-41	0.453703829	0.261	0.106	7.11E-37	adenocarcinoma
MYL12B	3.71E-41	0.684808399	0.643	0.443	7.92E-37	adenocarcinoma
TSTD1	3.84E-41	0.351510806	0.327	0.143	8.19E-37	adenocarcinoma
VMP11	8.04E-41	0.579322759	0.471	0.255	1.72E-36	adenocarcinoma
SFT2D11	2.80E-40	0.358411714	0.273	0.113	5.98E-36	adenocarcinoma
GNG51	2.83E-40	0.528763106	0.503	0.282	6.04E-36	adenocarcinoma
SYNGR21	3.33E-40	0.373690907	0.278	0.116	7.10E-36	adenocarcinoma
NDUFS4	3.60E-40	0.359798769	0.25	0.1	7.68E-36	adenocarcinoma
NDRG1	5.67E-40	0.789950302	0.413	0.218	1.21E-35	adenocarcinoma
CHCHD5	6.36E-40	0.339019304	0.263	0.108	1.36E-35	adenocarcinoma
NDUFB10	1.54E-39	0.541637973	0.436	0.231	3.29E-35	adenocarcinoma
CLINT1	1.82E-39	0.366099876	0.274	0.116	3.88E-35	adenocarcinoma
NDUFB41	1.82E-39	0.58926492	0.498	0.283	3.89E-35	adenocarcinoma
NARS	2.09E-39	0.426695756	0.278	0.119	4.46E-35	adenocarcinoma
NDUFAB11	3.95E-39	0.441053549	0.325	0.15	8.44E-35	adenocarcinoma
ANXA111	4.23E-39	0.55013215	0.369	0.183	9.04E-35	adenocarcinoma
BAG1	8.65E-39	0.504059685	0.325	0.152	1.85E-34	adenocarcinoma

FAM120A	1.88E-38	0.522801352	0.272	0.119	4.01E-34	adenocarcinoma
EZR1	2.38E-38	0.565658192	0.609	0.383	5.08E-34	adenocarcinoma
PHB2	2.73E-38	0.349843117	0.289	0.127	5.82E-34	adenocarcinoma
RPS6	1.34E-37	0.413623641	0.963	0.941	2.85E-33	adenocarcinoma
RPS27L1	1.36E-37	0.555696056	0.585	0.366	2.91E-33	adenocarcinoma
HSBP12	3.28E-37	0.514576908	0.355	0.176	6.99E-33	adenocarcinoma
LSM5	3.40E-37	0.543615228	0.318	0.15	7.25E-33	adenocarcinoma
NDUFA41	7.85E-37	0.636061636	0.633	0.436	1.67E-32	adenocarcinoma
PSMA4	9.29E-37	0.60760897	0.351	0.176	1.98E-32	adenocarcinoma
RPS111	1.51E-36	0.362071766	0.924	0.898	3.21E-32	adenocarcinoma
KLF3	1.78E-36	0.430669115	0.339	0.161	3.79E-32	adenocarcinoma
ATP5PD1	1.89E-36	0.515907486	0.412	0.218	4.03E-32	adenocarcinoma
LSM7	2.30E-36	0.454020419	0.389	0.2	4.92E-32	adenocarcinoma
TALDO11	2.35E-36	0.554666677	0.304	0.142	5.01E-32	adenocarcinoma
ATP5F1C1	4.24E-36	0.505816455	0.381	0.199	9.06E-32	adenocarcinoma
NHP2	4.38E-36	0.392080781	0.3	0.138	9.35E-32	adenocarcinoma
FIS11	9.57E-36	0.519775065	0.393	0.205	2.04E-31	adenocarcinoma
RAB132	1.18E-35	0.448566988	0.287	0.129	2.51E-31	adenocarcinoma
SNRPD1	1.20E-35	0.545740324	0.386	0.2	2.56E-31	adenocarcinoma
ACTN4	1.69E-35	0.402559821	0.42	0.22	3.62E-31	adenocarcinoma
UQCRFS1	2.01E-35	0.440820094	0.34	0.164	4.28E-31	adenocarcinoma
POLR1D	2.47E-35	0.454218402	0.409	0.218	5.26E-31	adenocarcinoma
PRELID11	2.92E-35	0.511280083	0.477	0.277	6.23E-31	adenocarcinoma
TXN2	3.89E-35	0.409900198	0.263	0.115	8.29E-31	adenocarcinoma
RPS121	4.35E-35	0.316852315	0.977	0.974	9.29E-31	adenocarcinoma
PPP1R14B	8.26E-35	0.338341215	0.25	0.107	1.76E-30	adenocarcinoma
RPS20	8.87E-35	0.422431649	0.939	0.904	1.89E-30	adenocarcinoma
SRP9	1.19E-34	0.502194541	0.303	0.144	2.55E-30	adenocarcinoma
AIMP1	1.67E-34	0.399953232	0.252	0.11	3.56E-30	adenocarcinoma
ENO11	2.33E-34	0.567796882	0.521	0.322	4.98E-30	adenocarcinoma
MT-CO31	2.51E-34	0.418454744	0.925	0.927	5.36E-30	adenocarcinoma
POMP1	2.78E-34	0.56467624	0.571	0.365	5.94E-30	adenocarcinoma
CANX1	5.22E-34	0.418471362	0.381	0.199	1.12E-29	adenocarcinoma
TMED21	5.41E-34	0.488959029	0.438	0.242	1.15E-29	adenocarcinoma
SNRPD3	7.45E-34	0.308047951	0.291	0.134	1.59E-29	adenocarcinoma
CAPNS11	1.51E-33	0.357832619	0.306	0.145	3.23E-29	adenocarcinoma
SNRPE	1.98E-33	0.466518176	0.38	0.198	4.22E-29	adenocarcinoma
MIEN1	2.94E-33	0.334461582	0.259	0.114	6.29E-29	adenocarcinoma
AP2S12	5.72E-33	0.492575863	0.409	0.218	1.22E-28	adenocarcinoma
MZT2B	1.08E-32	0.50767947	0.542	0.334	2.30E-28	adenocarcinoma
RER1	1.25E-32	0.420071344	0.301	0.145	2.67E-28	adenocarcinoma
RPL281	2.01E-32	0.322482022	0.989	0.974	4.28E-28	adenocarcinoma
RAB2A1	2.42E-32	0.500012287	0.413	0.229	5.16E-28	adenocarcinoma

MLEC	3.72E-32	0.383683216	0.299	0.145	7.94E-28	adenocarcinoma
SNRPF	5.83E-32	0.464027685	0.431	0.241	1.24E-27	adenocarcinoma
RPS161	6.16E-32	0.329121575	0.959	0.938	1.31E-27	adenocarcinoma
PSME21	9.35E-32	0.513327345	0.429	0.243	2.00E-27	adenocarcinoma
CHCHD2	9.43E-32	0.584318327	0.675	0.511	2.01E-27	adenocarcinoma
ALKBH7	1.35E-31	0.365145687	0.304	0.148	2.87E-27	adenocarcinoma
BANF1	1.91E-31	0.304023916	0.303	0.146	4.07E-27	adenocarcinoma
PSMB71	2.74E-31	0.426918616	0.331	0.169	5.85E-27	adenocarcinoma
LAMTOR21	4.14E-31	0.39231423	0.287	0.137	8.83E-27	adenocarcinoma
GNB21	4.22E-31	0.358278377	0.29	0.139	9.01E-27	adenocarcinoma
DYNLRB1	7.63E-31	0.588248386	0.378	0.205	1.63E-26	adenocarcinoma
MRPL511	1.08E-30	0.494161449	0.348	0.183	2.31E-26	adenocarcinoma
ATP5PB	1.23E-30	0.403384653	0.306	0.152	2.63E-26	adenocarcinoma
PTGES3	1.47E-30	0.438852496	0.457	0.267	3.14E-26	adenocarcinoma
CD632	1.61E-30	0.305138406	0.627	0.39	3.44E-26	adenocarcinoma
MRPL21	2.06E-30	0.28661178	0.252	0.114	4.40E-26	adenocarcinoma
PA2G4	2.46E-30	0.452119732	0.47	0.274	5.25E-26	adenocarcinoma
LRP101	3.35E-30	0.445582976	0.277	0.133	7.15E-26	adenocarcinoma
PTTG1IP1	7.43E-30	0.397754492	0.306	0.153	1.59E-25	adenocarcinoma
TUBB1	9.49E-30	0.494473116	0.419	0.238	2.03E-25	adenocarcinoma
MYDGF1	9.82E-30	0.417079279	0.363	0.193	2.10E-25	adenocarcinoma
C19orf701	1.09E-29	0.45487012	0.324	0.166	2.32E-25	adenocarcinoma
EIF4E2	1.64E-29	0.313404869	0.252	0.116	3.50E-25	adenocarcinoma
CMPK1	1.76E-29	0.410379939	0.353	0.187	3.76E-25	adenocarcinoma
APP1	3.97E-29	0.257213752	0.3	0.145	8.48E-25	adenocarcinoma
PSMB8	5.68E-29	0.367403291	0.36	0.189	1.21E-24	adenocarcinoma
UQCR11	6.23E-29	0.542252935	0.631	0.448	1.33E-24	adenocarcinoma
TMEM2191	8.46E-29	0.423982392	0.29	0.144	1.81E-24	adenocarcinoma
AKR1A1	8.73E-29	0.350426756	0.256	0.121	1.86E-24	adenocarcinoma
RPS81	9.84E-29	0.33238785	0.963	0.952	2.10E-24	adenocarcinoma
NAA38	1.12E-28	0.440824498	0.353	0.188	2.40E-24	adenocarcinoma
MZT2A	2.11E-28	0.430429646	0.4	0.227	4.50E-24	adenocarcinoma
FABP51	2.15E-28	0.462220158	0.311	0.161	4.59E-24	adenocarcinoma
GDI22	2.74E-28	0.450972185	0.32	0.166	5.86E-24	adenocarcinoma
RANBP1	3.19E-28	0.520500556	0.35	0.19	6.80E-24	adenocarcinoma
METTL26	3.21E-28	0.37594832	0.253	0.12	6.84E-24	adenocarcinoma
ACP1	3.52E-28	0.338641408	0.275	0.134	7.52E-24	adenocarcinoma
NDUFS7	4.16E-28	0.395816519	0.32	0.166	8.87E-24	adenocarcinoma
RPL7	4.19E-28	0.571701573	0.904	0.857	8.95E-24	adenocarcinoma
C8orf59	4.40E-28	0.481534983	0.293	0.149	9.40E-24	adenocarcinoma
RPS24	5.85E-28	0.340678553	0.947	0.92	1.25E-23	adenocarcinoma
FKBP22	2.50E-27	0.435183997	0.378	0.211	5.34E-23	adenocarcinoma
RPS9	2.68E-27	0.317248973	0.94	0.904	5.73E-23	adenocarcinoma

PLP2	4.01E-27	0.384455773	0.352	0.191	8.55E-23	adenocarcinoma
ADRM1	4.24E-27	0.382478423	0.276	0.137	9.05E-23	adenocarcinoma
TUFM	6.54E-27	0.346607087	0.32	0.167	1.40E-22	adenocarcinoma
COX17	9.21E-27	0.33912574	0.403	0.23	1.97E-22	adenocarcinoma
EIF2S2	1.15E-26	0.406367902	0.489	0.301	2.45E-22	adenocarcinoma
SNHG251	1.54E-26	0.734111595	0.447	0.294	3.29E-22	adenocarcinoma
MT-ND4L	2.01E-26	0.384659	0.529	0.337	4.28E-22	adenocarcinoma
MT-ATP8	2.72E-26	0.297262778	0.258	0.126	5.81E-22	adenocarcinoma
HADHA	4.09E-26	0.288180043	0.281	0.14	8.74E-22	adenocarcinoma
SNRPD2	5.95E-26	0.550167341	0.579	0.393	1.27E-21	adenocarcinoma
TMED101	7.66E-26	0.423571404	0.342	0.187	1.63E-21	adenocarcinoma
SLC25A3	7.69E-26	0.512683695	0.554	0.38	1.64E-21	adenocarcinoma
GADD45G	1.03E-25	0.414318024	0.378	0.214	2.19E-21	adenocarcinoma
CSKMT	1.35E-25	0.290139471	0.297	0.152	2.88E-21	adenocarcinoma
SNRPG	2.67E-25	0.489396498	0.435	0.263	5.70E-21	adenocarcinoma
SCAND1	3.61E-25	0.382362347	0.369	0.21	7.70E-21	adenocarcinoma
ARF41	4.03E-25	0.272940605	0.376	0.209	8.60E-21	adenocarcinoma
MT-ND4	4.34E-25	0.329727031	0.936	0.926	9.26E-21	adenocarcinoma
FBL	6.20E-25	0.283260086	0.321	0.171	1.32E-20	adenocarcinoma
MRPL54	6.31E-25	0.33633878	0.285	0.146	1.35E-20	adenocarcinoma
CIB11	9.26E-25	0.377119285	0.46	0.279	1.98E-20	adenocarcinoma
EIF3I	1.15E-24	0.357590617	0.335	0.185	2.45E-20	adenocarcinoma
SELENOH	1.22E-24	0.424904257	0.379	0.217	2.61E-20	adenocarcinoma
DBI1	1.64E-24	0.428591459	0.523	0.342	3.50E-20	adenocarcinoma
GPX42	2.30E-24	0.429675207	0.527	0.337	4.91E-20	adenocarcinoma
PPP1CA	3.00E-24	0.302345927	0.333	0.182	6.41E-20	adenocarcinoma
LAMTOR41	3.29E-24	0.419241618	0.445	0.273	7.02E-20	adenocarcinoma
CALR2	4.30E-24	0.447531255	0.541	0.354	9.18E-20	adenocarcinoma
SNRPB	6.01E-24	0.382169686	0.407	0.239	1.28E-19	adenocarcinoma
RPS22	1.02E-23	0.406964707	0.966	0.96	2.18E-19	adenocarcinoma
NDUFA12	1.58E-23	0.285677334	0.297	0.158	3.37E-19	adenocarcinoma
CHMP31	2.63E-23	0.25477181	0.292	0.154	5.60E-19	adenocarcinoma
NENF1	3.39E-23	0.284874822	0.358	0.2	7.24E-19	adenocarcinoma
SF3B6	5.22E-23	0.422842489	0.38	0.224	1.11E-18	adenocarcinoma
RAB1A2	5.30E-23	0.306102994	0.312	0.17	1.13E-18	adenocarcinoma
RPL31	9.67E-23	0.277459399	0.907	0.899	2.06E-18	adenocarcinoma
CAMTA11	2.52E-22	0.304859502	0.278	0.147	5.37E-18	adenocarcinoma
NDUFA2	2.71E-22	0.339681903	0.387	0.23	5.79E-18	adenocarcinoma
CCT6A	3.21E-22	0.31547986	0.261	0.137	6.85E-18	adenocarcinoma
TMEM1471	3.22E-22	0.392909457	0.294	0.163	6.87E-18	adenocarcinoma
NDUFB2	3.58E-22	0.449720155	0.495	0.323	7.64E-18	adenocarcinoma
TIMM8B	6.87E-22	0.383424948	0.258	0.136	1.47E-17	adenocarcinoma
MRPL20	7.54E-22	0.260130212	0.368	0.211	1.61E-17	adenocarcinoma

PSMB3	2.06E-21	0.424630856	0.32	0.183	4.39E-17	adenocarcinoma
EEF2	2.17E-21	0.431369176	0.701	0.566	4.63E-17	adenocarcinoma
DYNLT11	3.05E-21	0.311111151	0.315	0.175	6.52E-17	adenocarcinoma
LY6E1	3.41E-21	0.545499111	0.38	0.235	7.28E-17	adenocarcinoma
CALM21	3.54E-21	0.466245043	0.661	0.496	7.55E-17	adenocarcinoma
LAMTOR5	3.66E-21	0.277025049	0.383	0.229	7.80E-17	adenocarcinoma
NDUFV2	4.03E-21	0.263032259	0.37	0.216	8.61E-17	adenocarcinoma
NOP101	5.75E-21	0.288244984	0.429	0.263	1.23E-16	adenocarcinoma
MRPL57	1.53E-20	0.370850661	0.323	0.186	3.26E-16	adenocarcinoma
SQSTM12	1.72E-20	0.460139031	0.535	0.351	3.67E-16	adenocarcinoma
NDUFB12	2.26E-20	0.399640134	0.473	0.328	4.83E-16	adenocarcinoma
YWHAH1	2.93E-20	0.308335442	0.345	0.202	6.26E-16	adenocarcinoma
GUK1	4.26E-20	0.392490571	0.618	0.452	9.08E-16	adenocarcinoma
ANAPC111	4.65E-20	0.377279645	0.363	0.217	9.93E-16	adenocarcinoma
HDLBP1	1.04E-19	0.285823006	0.279	0.154	2.21E-15	adenocarcinoma
CD591	1.05E-19	0.281539047	0.29	0.161	2.23E-15	adenocarcinoma
ATP5PF1	1.12E-19	0.379111254	0.445	0.289	2.39E-15	adenocarcinoma
DAD11	1.21E-19	0.287707244	0.352	0.21	2.58E-15	adenocarcinoma
YWHAZ	1.39E-19	0.374120235	0.639	0.502	2.97E-15	adenocarcinoma
RPL141	1.53E-19	0.336887896	0.89	0.882	3.27E-15	adenocarcinoma
NDUFA3	2.02E-19	0.359847766	0.399	0.253	4.31E-15	adenocarcinoma
PDIA61	2.27E-19	0.388435014	0.326	0.192	4.85E-15	adenocarcinoma
NDUFA1	3.02E-19	0.420821304	0.494	0.337	6.44E-15	adenocarcinoma
BLOC1S11	3.42E-19	0.364376001	0.385	0.24	7.29E-15	adenocarcinoma
VPS28	3.95E-19	0.331415564	0.373	0.227	8.43E-15	adenocarcinoma
MRPS211	5.49E-19	0.289760685	0.374	0.228	1.17E-14	adenocarcinoma
SLC25A6	7.33E-19	0.345187338	0.707	0.56	1.56E-14	adenocarcinoma
STAU1	8.26E-19	0.271240722	0.293	0.167	1.76E-14	adenocarcinoma
NSA2	8.93E-19	0.288091821	0.475	0.309	1.91E-14	adenocarcinoma
NDUFS51	1.56E-18	0.372702589	0.609	0.45	3.33E-14	adenocarcinoma
NDUFS8	1.63E-18	0.340600767	0.299	0.172	3.47E-14	adenocarcinoma
GSTO11	1.95E-18	0.288628611	0.315	0.183	4.16E-14	adenocarcinoma
SET	2.24E-18	0.473418417	0.537	0.371	4.78E-14	adenocarcinoma
TAX1BP11	2.31E-18	0.377284807	0.38	0.238	4.93E-14	adenocarcinoma
RPL26	6.64E-18	0.313671353	0.933	0.934	1.42E-13	adenocarcinoma
POLR2L1	9.96E-18	0.380230509	0.577	0.411	2.13E-13	adenocarcinoma
PEBP11	2.44E-17	0.304198321	0.432	0.279	5.20E-13	adenocarcinoma
TBCA1	2.45E-17	0.32723309	0.552	0.394	5.22E-13	adenocarcinoma
PFN11	3.15E-17	0.310873356	0.742	0.592	6.72E-13	adenocarcinoma
PPIA	3.36E-17	0.415140866	0.713	0.609	7.17E-13	adenocarcinoma
ATP5MC2	4.00E-17	0.364369301	0.649	0.539	8.54E-13	adenocarcinoma
COX7A2L	6.34E-17	0.276750601	0.317	0.189	1.35E-12	adenocarcinoma
NORAD1	7.20E-17	0.334102402	0.253	0.143	1.54E-12	adenocarcinoma

SSNA1	8.65E-17	0.2840021	0.255	0.144	1.85E-12	adenocarcinoma
TUBB4B	9.59E-17	0.424865883	0.491	0.343	2.05E-12	adenocarcinoma
POLR2J	9.84E-17	0.28068872	0.291	0.17	2.10E-12	adenocarcinoma
EIF3E	9.98E-17	0.449661108	0.556	0.416	2.13E-12	adenocarcinoma
AUP1	1.29E-16	0.274184799	0.25	0.141	2.74E-12	adenocarcinoma
ATP5F1A	1.86E-16	0.257375513	0.325	0.194	3.98E-12	adenocarcinoma
ANP32B	2.37E-16	0.391942222	0.403	0.264	5.06E-12	adenocarcinoma
SSBP1	2.65E-16	0.275457701	0.317	0.193	5.66E-12	adenocarcinoma
MT-ND5	2.75E-16	0.341776673	0.74	0.617	5.86E-12	adenocarcinoma
HIST1H4C	7.69E-16	0.392358133	0.434	0.293	1.64E-11	adenocarcinoma
C4orf481	1.73E-15	0.381543434	0.303	0.191	3.68E-11	adenocarcinoma
HSP90B11	1.91E-15	0.337788951	0.538	0.377	4.08E-11	adenocarcinoma
LSM3	2.00E-15	0.263196067	0.262	0.154	4.28E-11	adenocarcinoma
RPN2	2.15E-15	0.297901347	0.254	0.147	4.59E-11	adenocarcinoma
CFL11	4.33E-15	0.321426123	0.745	0.629	9.25E-11	adenocarcinoma
SSR32	5.55E-15	0.351023111	0.361	0.231	1.18E-10	adenocarcinoma
RPS28	7.91E-15	0.260936154	0.959	0.962	1.69E-10	adenocarcinoma
RALY	8.10E-15	0.300670876	0.254	0.149	1.73E-10	adenocarcinoma
UQCRB	8.40E-15	0.406419238	0.697	0.556	1.79E-10	adenocarcinoma
TUBA1B2	1.44E-14	0.384174223	0.47	0.327	3.08E-10	adenocarcinoma
RAN	2.05E-14	0.315426827	0.502	0.358	4.38E-10	adenocarcinoma
PSMB11	2.32E-14	0.261884105	0.378	0.245	4.94E-10	adenocarcinoma
NDUFA111	5.82E-14	0.251457634	0.398	0.262	1.24E-09	adenocarcinoma
CYCS	9.41E-14	0.264432205	0.476	0.33	2.01E-09	adenocarcinoma
RPL10A	1.24E-13	0.300636425	0.873	0.861	2.65E-09	adenocarcinoma
PHPT11	1.33E-13	0.273826448	0.328	0.21	2.84E-09	adenocarcinoma
TMEM160	3.39E-13	0.350204354	0.254	0.154	7.23E-09	adenocarcinoma
COPE	8.59E-13	0.287395608	0.355	0.234	1.83E-08	adenocarcinoma
EEF1B21	1.41E-12	0.286300719	0.725	0.647	3.00E-08	adenocarcinoma
MINOS11	4.65E-12	0.260524677	0.331	0.218	9.93E-08	adenocarcinoma
HSPA51	4.99E-12	0.354888865	0.33	0.221	1.07E-07	adenocarcinoma
ARPC4	2.77E-11	0.269496953	0.292	0.189	5.91E-07	adenocarcinoma
NDUFC21	9.73E-11	0.268189426	0.287	0.191	2.08E-06	adenocarcinoma
NACA	2.16E-10	0.267788428	0.786	0.738	4.62E-06	adenocarcinoma
C9orf16	2.62E-10	0.295270915	0.445	0.318	5.60E-06	adenocarcinoma
LDHA1	5.35E-09	0.279810515	0.553	0.443	0.000114	adenocarcinoma
PSMA71	1.35E-06	0.314973233	0.566	0.475	0.028889	adenocarcinoma
NPM11	1.74E-06	0.322849861	0.616	0.55	0.03708	adenocarcinoma
H2AFZ	0.000131	0.258105048	0.503	0.413	1	adenocarcinoma
PPP1R15A	0.002924	0.267031866	0.468	0.397	1	adenocarcinoma
STC1	0	4.476574166	0.536	0.015	0	Endothelial
TM4SF11	0	3.613082821	0.915	0.155	0	Endothelial
ACKR1	0	3.267556472	0.509	0.007	0	Endothelial

SPRY11	0	3.239980286	0.83	0.118	0	Endothelial
PECAM1	0	3.17698626	0.817	0.043	0	Endothelial
PLVAP	0	3.065925963	0.694	0.013	0	Endothelial
VWF	0	3.010470698	0.732	0.014	0	Endothelial
CLDN5	0	2.963869878	0.568	0.006	0	Endothelial
AQP1	0	2.963167054	0.647	0.013	0	Endothelial
PCAT19	0	2.922162791	0.77	0.007	0	Endothelial
RAMP2	0	2.803754824	0.739	0.017	0	Endothelial
SPARCL11	0	2.76203821	0.888	0.125	0	Endothelial
HSPG2	0	2.750171056	0.726	0.059	0	Endothelial
GNG111	0	2.710767303	0.78	0.064	0	Endothelial
HES12	0	2.666106161	0.773	0.152	0	Endothelial
TCF41	0	2.640391017	0.826	0.122	0	Endothelial
CAV11	0	2.541185318	0.72	0.082	0	Endothelial
ADGRL4	0	2.527472866	0.659	0.005	0	Endothelial
CRIP21	0	2.465676062	0.741	0.065	0	Endothelial
PDLIM12	0	2.419143786	0.771	0.126	0	Endothelial
COL4A11	0	2.400167539	0.617	0.071	0	Endothelial
IGFBP71	0	2.364847355	0.902	0.196	0	Endothelial
SERPINE11	0	2.3352676	0.574	0.065	0	Endothelial
ENG	0	2.30107222	0.647	0.049	0	Endothelial
FKBP1A	0	2.299766827	0.855	0.26	0	Endothelial
SLC9A3R2	0	2.298301951	0.485	0.043	0	Endothelial
COL15A11	0	2.288651608	0.579	0.041	0	Endothelial
INSR	0	2.247682781	0.532	0.047	0	Endothelial
EMP11	0	2.245707431	0.774	0.139	0	Endothelial
EPAS1	0	2.229306703	0.671	0.067	0	Endothelial
PALMD	0	2.229152134	0.558	0.005	0	Endothelial
COL4A21	0	2.228411729	0.636	0.071	0	Endothelial
RAMP3	0	2.203660607	0.586	0.008	0	Endothelial
CALCRL	0	2.185796327	0.583	0.012	0	Endothelial
CD93	0	2.170601561	0.627	0.023	0	Endothelial
SELE	0	2.134919403	0.294	0.002	0	Endothelial
CD592	0	2.089411637	0.748	0.138	0	Endothelial
ECSCR	0	2.070034022	0.623	0.004	0	Endothelial
IFITM32	0	2.004682235	0.924	0.287	0	Endothelial
NPDC1	0	1.982739485	0.638	0.056	0	Endothelial
ARHGAP29	0	1.968855962	0.577	0.025	0	Endothelial
CAVIN2	0	1.938647808	0.515	0.014	0	Endothelial
EGFL7	0	1.894808649	0.483	0.01	0	Endothelial
A2M1	0	1.838054286	0.606	0.095	0	Endothelial
VWA1	0	1.837494501	0.486	0.016	0	Endothelial
PRSS231	0	1.832714983	0.589	0.069	0	Endothelial

ESAM	0	1.824756478	0.526	0.016	0	Endothelial
MTUS1	0	1.824234637	0.545	0.044	0	Endothelial
SLCO2A1	0	1.796809566	0.456	0.004	0	Endothelial
CAVIN11	0	1.783668727	0.676	0.1	0	Endothelial
NFIB1	0	1.76666408	0.585	0.057	0	Endothelial
CLEC14A	0	1.753271299	0.48	0.007	0	Endothelial
HYAL2	0	1.741962734	0.488	0.021	0	Endothelial
F2RL3	0	1.729093708	0.37	0.006	0	Endothelial
STOM1	0	1.723627849	0.645	0.115	0	Endothelial
FLT1	0	1.718106307	0.418	0.012	0	Endothelial
ADAMTS9	0	1.667429782	0.436	0.014	0	Endothelial
TGFBR2	0	1.65952464	0.582	0.095	0	Endothelial
CNKSR3	0	1.652710995	0.386	0.014	0	Endothelial
WWTR1	0	1.645634582	0.506	0.042	0	Endothelial
RAB133	0	1.63304614	0.638	0.113	0	Endothelial
RNASE11	0	1.627987516	0.571	0.054	0	Endothelial
CCL14	0	1.616809487	0.276	0.001	0	Endothelial
PODXL	0	1.578926821	0.359	0.009	0	Endothelial
EMCN	0	1.551861292	0.447	0.001	0	Endothelial
PCDH17	0	1.540319198	0.392	0.004	0	Endothelial
SPARC1	0	1.51918427	0.727	0.146	0	Endothelial
CD34	0	1.514567987	0.408	0.017	0	Endothelial
BMPR2	0	1.512161776	0.508	0.062	0	Endothelial
DUSP6	0	1.48141547	0.498	0.065	0	Endothelial
SWAP70	0	1.468542844	0.497	0.056	0	Endothelial
CDH5	0	1.456723868	0.418	0.003	0	Endothelial
ADAM15	0	1.379933498	0.442	0.028	0	Endothelial
MCAM	0	1.367076334	0.429	0.038	0	Endothelial
C1orf54	0	1.363220389	0.45	0.049	0	Endothelial
MAGI1	0	1.358232466	0.391	0.018	0	Endothelial
CRIM1	0	1.343301184	0.433	0.039	0	Endothelial
ITGA61	0	1.342225872	0.421	0.047	0	Endothelial
S1PR1	0	1.330804004	0.444	0.028	0	Endothelial
MEF2C	0	1.328171184	0.552	0.074	0	Endothelial
PTPRB	0	1.327790635	0.368	0.004	0	Endothelial
GJA1	0	1.326308325	0.38	0.026	0	Endothelial
NOTCH4	0	1.30912287	0.305	0.007	0	Endothelial
HEG1	0	1.306050746	0.392	0.038	0	Endothelial
LDB2	0	1.305542379	0.392	0.009	0	Endothelial
PLXND1	0	1.304391296	0.424	0.036	0	Endothelial
TSPAN7	0	1.304177725	0.339	0.002	0	Endothelial
TGM2	0	1.302884973	0.439	0.048	0	Endothelial
RAI14	0	1.286619444	0.414	0.029	0	Endothelial

C2CD4B	0	1.269988363	0.3	0.007	0	Endothelial
JAM2	0	1.234481548	0.348	0.01	0	Endothelial
ADGRF5	0	1.233773632	0.314	0.007	0	Endothelial
ADCY4	0	1.230601547	0.329	0.004	0	Endothelial
TMEM255I	0	1.220213806	0.373	0.008	0	Endothelial
NUAK1	0	1.219776621	0.353	0.015	0	Endothelial
CXorf36	0	1.218072373	0.333	0.002	0	Endothelial
DOCK9	0	1.216965927	0.365	0.017	0	Endothelial
MCTP1	0	1.19732899	0.339	0.014	0	Endothelial
MMRN2	0	1.149051316	0.345	0.002	0	Endothelial
RHOJ	0	1.144446558	0.341	0.008	0	Endothelial
BCAM	0	1.129112231	0.317	0.01	0	Endothelial
FAM167B	0	1.119485331	0.318	0.004	0	Endothelial
DLC1	0	1.097565289	0.342	0.028	0	Endothelial
ACVRL1	0	1.075584271	0.348	0.028	0	Endothelial
ZNF385D	0	1.075131735	0.298	0.008	0	Endothelial
PKP4	0	1.070652753	0.344	0.027	0	Endothelial
SELP	0	1.068766168	0.262	0.001	0	Endothelial
GRB10	0	1.065946924	0.298	0.013	0	Endothelial
MYCT1	0	1.060798403	0.277	0.001	0	Endothelial
CYYR1	0	1.057343198	0.323	0.002	0	Endothelial
ERG	0	1.043421231	0.317	0.003	0	Endothelial
RND1	0	1.0368515	0.308	0.016	0	Endothelial
SASH1	0	1.012697668	0.333	0.017	0	Endothelial
MKL2	0	1.006058955	0.277	0.011	0	Endothelial
SOX7	0	1.005834367	0.318	0.003	0	Endothelial
KDR	0	1.005699859	0.265	0.001	0	Endothelial
FILIP1	0	0.989663791	0.28	0.014	0	Endothelial
TIE1	0	0.982730103	0.312	0.003	0	Endothelial
MGP1	0	0.972266755	0.8	0.127	0	Endothelial
LMO2	0	0.961858545	0.294	0.007	0	Endothelial
PTPRM	0	0.961319932	0.282	0.015	0	Endothelial
MECOM	0	0.959209011	0.277	0.017	0	Endothelial
THSD7A	0	0.955519841	0.261	0.002	0	Endothelial
LIMS2	0	0.949783822	0.297	0.014	0	Endothelial
SHROOM4	0	0.938747755	0.274	0.003	0	Endothelial
SNCG	0	0.910671134	0.259	0.008	0	Endothelial
RAPGEF4	0	0.904680604	0.255	0.001	0	Endothelial
SYNPO	0	0.87442224	0.28	0.014	0	Endothelial
PLXNA2	0	0.855758926	0.255	0.014	0	Endothelial
IL3RA	0	0.837910362	0.252	0.008	0	Endothelial
PRCP	9.60E-308	1.45781235	0.418	0.048	2.05E-303	Endothelial
ECE1	5.71E-304	1.085117273	0.394	0.041	1.22E-299	Endothelial

NEDD9	3.10E-301	1.444010111	0.533	0.08	6.61E-297	Endothelial
THBD	4.53E-297	1.267992376	0.386	0.04	9.68E-293	Endothelial
RDX1	5.66E-297	1.809695629	0.683	0.147	1.21E-292	Endothelial
APOLD1	2.74E-291	1.287159538	0.435	0.052	5.84E-287	Endothelial
EDN1	6.19E-291	1.991066431	0.297	0.023	1.32E-286	Endothelial
APP2	3.82E-287	1.621197563	0.644	0.129	8.15E-283	Endothelial
EFNA1	1.08E-286	1.149775551	0.302	0.024	2.30E-282	Endothelial
ITGA5	8.52E-286	1.386749382	0.459	0.061	1.82E-281	Endothelial
COL18A11	9.33E-281	1.167998271	0.465	0.063	1.99E-276	Endothelial
NOSTRIN	1.54E-279	0.861087088	0.253	0.016	3.29E-275	Endothelial
LAMA41	2.37E-277	1.297075233	0.394	0.046	5.05E-273	Endothelial
HTRA11	6.28E-275	1.292940848	0.48	0.069	1.34E-270	Endothelial
ICAM11	1.43E-274	1.617636911	0.515	0.085	3.05E-270	Endothelial
IFI272	6.30E-274	1.788031291	0.865	0.246	1.34E-269	Endothelial
IGFBP41	9.85E-272	1.336675924	0.676	0.135	2.10E-267	Endothelial
ETS22	1.27E-268	1.466234913	0.624	0.126	2.72E-264	Endothelial
HDGFL3	1.54E-268	1.002671994	0.35	0.037	3.29E-264	Endothelial
FAM198B	1.03E-266	1.065621446	0.358	0.039	2.20E-262	Endothelial
TINAGL1	2.04E-264	0.979308655	0.368	0.041	4.36E-260	Endothelial
KIAA0355	1.00E-261	1.281624194	0.539	0.091	2.14E-257	Endothelial
NRP1	1.41E-261	1.043331721	0.344	0.037	3.00E-257	Endothelial
TJP1	4.64E-261	1.113834477	0.423	0.057	9.90E-257	Endothelial
PLPP1	1.00E-259	1.502019719	0.365	0.042	2.14E-255	Endothelial
ID11	7.48E-258	1.769378086	0.536	0.096	1.60E-253	Endothelial
ELK3	4.64E-257	1.464139609	0.586	0.115	9.91E-253	Endothelial
DPYSL21	1.88E-254	1.36568808	0.514	0.091	4.01E-250	Endothelial
FAM213A	1.78E-244	1.240617168	0.376	0.048	3.80E-240	Endothelial
ID31	4.13E-242	1.610093675	0.629	0.138	8.81E-238	Endothelial
CAV2	2.07E-241	1.094435739	0.365	0.045	4.42E-237	Endothelial
YBX31	6.26E-238	1.678867674	0.747	0.215	1.34E-233	Endothelial
MALL	9.43E-238	0.915313277	0.327	0.036	2.01E-233	Endothelial
EHD2	1.91E-236	0.973597032	0.33	0.037	4.08E-232	Endothelial
KLF7	1.50E-232	0.953088783	0.302	0.031	3.21E-228	Endothelial
CLIC41	1.55E-232	1.204102983	0.409	0.061	3.32E-228	Endothelial
TNFAIP1	3.32E-232	0.859904997	0.311	0.033	7.08E-228	Endothelial
POSTN1	3.79E-231	1.481442338	0.368	0.047	8.10E-227	Endothelial
ADIRF2	4.85E-230	1.653575532	0.609	0.139	1.04E-225	Endothelial
CALD11	1.49E-227	0.404274655	0.732	0.159	3.19E-223	Endothelial
EFNB2	7.84E-225	1.340921365	0.32	0.037	1.67E-220	Endothelial
DUSP23	1.61E-224	1.321198992	0.415	0.066	3.44E-220	Endothelial
SPTBN12	4.41E-221	1.446240013	0.611	0.143	9.41E-217	Endothelial
PIK3C2A	2.00E-215	1.017130778	0.409	0.063	4.28E-211	Endothelial
CD94	3.13E-215	1.599223285	0.67	0.177	6.67E-211	Endothelial

PNP	4.16E-214	1.104109929	0.406	0.063	8.87E-210	Endothelial
RASAL2	4.78E-213	0.825129334	0.286	0.031	1.02E-208	Endothelial
NNMT1	6.95E-210	1.267884051	0.547	0.111	1.48E-205	Endothelial
ABL22	8.95E-210	1.261049827	0.536	0.113	1.91E-205	Endothelial
PLPP31	1.23E-209	1.127646134	0.338	0.044	2.63E-205	Endothelial
SOCS32	6.88E-203	1.783730269	0.603	0.156	1.47E-198	Endothelial
PKIG1	1.45E-202	1.065108045	0.415	0.069	3.10E-198	Endothelial
NCOA7	9.56E-202	1.631053591	0.571	0.139	2.04E-197	Endothelial
CTTNBP2N	1.14E-201	0.930005796	0.315	0.04	2.42E-197	Endothelial
CNN31	4.95E-201	1.072397981	0.488	0.094	1.06E-196	Endothelial
RPGR	1.15E-200	1.131453798	0.37	0.055	2.46E-196	Endothelial
EHD4	1.22E-197	0.953510731	0.389	0.062	2.61E-193	Endothelial
NECTIN2	1.72E-196	0.982437031	0.38	0.061	3.67E-192	Endothelial
CTGF1	4.00E-196	1.372392818	0.552	0.123	8.54E-192	Endothelial
DSTN2	8.62E-195	1.38457554	0.867	0.365	1.84E-190	Endothelial
S100A162	1.55E-191	1.180736076	0.542	0.12	3.30E-187	Endothelial
NR2F21	2.62E-188	1.126343438	0.373	0.061	5.59E-184	Endothelial
COX7A11	1.04E-187	1.061927598	0.4	0.069	2.22E-183	Endothelial
MGST21	6.22E-185	0.947575652	0.392	0.068	1.33E-180	Endothelial
TCIM	6.24E-185	1.112947585	0.314	0.043	1.33E-180	Endothelial
SHC1	8.61E-184	0.922231982	0.338	0.051	1.84E-179	Endothelial
CDC42EP3	1.53E-183	1.04940375	0.436	0.082	3.26E-179	Endothelial
DPYSL31	5.70E-183	0.939181267	0.297	0.039	1.22E-178	Endothelial
ICAM2	1.19E-180	1.015208461	0.288	0.036	2.54E-176	Endothelial
FAM241A	5.16E-176	0.947602966	0.305	0.042	1.10E-171	Endothelial
CAVIN31	9.56E-176	1.157598173	0.4	0.074	2.04E-171	Endothelial
MAP1B1	1.74E-174	0.983754793	0.352	0.056	3.70E-170	Endothelial
TRIOBP	1.17E-170	0.785298333	0.314	0.047	2.50E-166	Endothelial
SH3BP51	1.48E-165	0.957467299	0.398	0.077	3.17E-161	Endothelial
S100A131	5.63E-165	1.068724529	0.526	0.129	1.20E-160	Endothelial
ADGRG1	7.94E-165	0.631070724	0.261	0.032	1.70E-160	Endothelial
CTNND11	6.75E-164	0.86948096	0.338	0.057	1.44E-159	Endothelial
CCDC501	4.98E-163	0.948453111	0.445	0.097	1.06E-158	Endothelial
CYR611	5.69E-163	1.190747433	0.458	0.099	1.21E-158	Endothelial
CMIP	1.63E-162	1.000743651	0.486	0.113	3.48E-158	Endothelial
ADAMTS1	4.42E-162	1.248125832	0.32	0.051	9.43E-158	Endothelial
PTMS1	1.83E-161	1.253520318	0.7	0.232	3.90E-157	Endothelial
FLNB1	3.10E-161	0.956012261	0.367	0.067	6.63E-157	Endothelial
TSHZ21	7.59E-161	1.023769238	0.512	0.121	1.62E-156	Endothelial
NDRG11	1.83E-160	1.292531222	0.665	0.208	3.90E-156	Endothelial
PON2	2.02E-160	0.693176983	0.252	0.032	4.32E-156	Endothelial
PTPRK	4.55E-160	0.810380924	0.291	0.043	9.72E-156	Endothelial
PLS3	1.16E-159	0.876848915	0.264	0.035	2.47E-155	Endothelial

KLF10	1.96E-158	0.954016408	0.4	0.081	4.19E-154	Endothelial
SULF2	5.49E-156	0.90239013	0.335	0.058	1.17E-151	Endothelial
NRP2	6.09E-156	1.061103409	0.327	0.056	1.30E-151	Endothelial
PDLIM51	4.21E-155	1.019337529	0.427	0.094	9.00E-151	Endothelial
ACTN11	5.27E-154	1.096987931	0.424	0.093	1.13E-149	Endothelial
CCDC85B	8.42E-154	1.236577042	0.62	0.194	1.80E-149	Endothelial
TCEAL91	3.73E-152	1.064711828	0.541	0.142	7.97E-148	Endothelial
GIMAP4	4.31E-151	0.742022771	0.373	0.069	9.21E-147	Endothelial
VAMP52	1.09E-150	1.211268851	0.568	0.164	2.32E-146	Endothelial
TSC22D11	1.89E-148	1.31288237	0.532	0.144	4.03E-144	Endothelial
MTRNR2L1	6.38E-146	1.419241804	0.5	0.134	1.36E-141	Endothelial
RAB11A1	2.83E-145	0.962551967	0.503	0.132	6.04E-141	Endothelial
HIPK3	7.66E-145	0.886389298	0.332	0.06	1.63E-140	Endothelial
MEF2A	5.27E-143	0.886540713	0.382	0.08	1.12E-138	Endothelial
KTN1	6.87E-143	1.253023396	0.808	0.368	1.47E-138	Endothelial
CD320	2.69E-142	0.998704684	0.292	0.048	5.74E-138	Endothelial
RHOC2	1.77E-140	1.118008348	0.598	0.187	3.77E-136	Endothelial
SAV1	1.75E-139	0.667217008	0.288	0.047	3.74E-135	Endothelial
JAG1	6.12E-139	0.987457669	0.282	0.046	1.31E-134	Endothelial
YPEL2	2.23E-138	0.766984779	0.333	0.062	4.75E-134	Endothelial
PPFIBP11	3.40E-138	0.794928758	0.294	0.051	7.25E-134	Endothelial
GNAI21	4.71E-137	1.125148395	0.648	0.226	1.01E-132	Endothelial
BACE2	1.31E-136	0.801328143	0.312	0.057	2.80E-132	Endothelial
LUZP1	1.75E-136	1.007175988	0.512	0.14	3.74E-132	Endothelial
TFPI1	1.15E-134	1.107472853	0.333	0.066	2.45E-130	Endothelial
AFDN	2.17E-133	0.845153682	0.308	0.057	4.64E-129	Endothelial
SOX41	2.43E-133	0.934598909	0.586	0.179	5.19E-129	Endothelial
PDK4	4.02E-133	0.81731436	0.276	0.045	8.58E-129	Endothelial
PTTG1IP2	4.74E-133	1.013193417	0.509	0.145	1.01E-128	Endothelial
FLI1	9.29E-133	0.621167898	0.256	0.038	1.98E-128	Endothelial
SSFA21	1.09E-132	0.74267024	0.312	0.058	2.34E-128	Endothelial
ITM2B2	1.43E-132	1.342081845	0.841	0.473	3.05E-128	Endothelial
HDAC7	2.50E-132	0.804848985	0.305	0.055	5.33E-128	Endothelial
EMP21	3.03E-132	0.836678964	0.333	0.066	6.46E-128	Endothelial
KCTD121	6.30E-132	1.023304556	0.315	0.061	1.34E-127	Endothelial
RHOB2	7.63E-132	1.252397068	0.614	0.206	1.63E-127	Endothelial
IL6ST1	1.10E-131	0.97057466	0.523	0.15	2.36E-127	Endothelial
TCEAL41	3.04E-131	0.904899597	0.432	0.107	6.49E-127	Endothelial
ASAP12	1.72E-130	0.842620243	0.417	0.1	3.68E-126	Endothelial
FERMT21	2.03E-129	0.672042357	0.314	0.059	4.32E-125	Endothelial
KLF43	2.48E-129	1.120355621	0.464	0.126	5.29E-125	Endothelial
UACA1	5.42E-129	0.879254282	0.295	0.054	1.16E-124	Endothelial
VIM2	4.07E-128	1.270509716	0.905	0.586	8.68E-124	Endothelial

CLU1	9.51E-127	1.461947831	0.386	0.091	2.03E-122	Endothelial
MYOF	1.13E-126	0.776965057	0.288	0.053	2.41E-122	Endothelial
RBPMS1	1.74E-126	0.70151705	0.262	0.043	3.72E-122	Endothelial
NAA381	3.61E-126	1.036339719	0.561	0.18	7.70E-122	Endothelial
SELENOW2	7.48E-126	1.125090521	0.741	0.304	1.60E-121	Endothelial
PPIC1	1.09E-125	0.761686558	0.356	0.076	2.33E-121	Endothelial
NFE2L1	1.99E-124	0.82331954	0.355	0.079	4.24E-120	Endothelial
IFITM21	3.38E-124	1.011600888	0.823	0.374	7.22E-120	Endothelial
DYNC1I21	4.47E-124	0.98572918	0.542	0.169	9.55E-120	Endothelial
KLF2	7.04E-124	1.132193948	0.57	0.183	1.50E-119	Endothelial
PTPN12	7.22E-124	0.743019921	0.391	0.093	1.54E-119	Endothelial
TIMP31	8.78E-124	0.986803581	0.432	0.108	1.87E-119	Endothelial
CD812	3.06E-123	1.110438787	0.568	0.184	6.52E-119	Endothelial
GSN1	6.87E-123	0.334999986	0.521	0.152	1.47E-118	Endothelial
FNIP21	1.69E-122	0.850630012	0.362	0.081	3.62E-118	Endothelial
CD1512	5.39E-122	0.868285137	0.456	0.124	1.15E-117	Endothelial
CHMP32	7.05E-122	0.911775199	0.495	0.146	1.51E-117	Endothelial
SNTB2	7.06E-122	0.691343332	0.33	0.068	1.51E-117	Endothelial
NCKAP1	1.08E-121	0.743066697	0.271	0.048	2.31E-117	Endothelial
LAMC11	3.81E-121	0.645616677	0.283	0.052	8.13E-117	Endothelial
CYB5R31	9.01E-121	1.003682671	0.509	0.153	1.92E-116	Endothelial
BHLHE40	9.41E-121	0.972892926	0.491	0.144	2.01E-116	Endothelial
TNFRSF1A	9.83E-121	0.747037232	0.362	0.083	2.10E-116	Endothelial
TRIB11	1.40E-120	0.941180919	0.427	0.113	2.98E-116	Endothelial
UPP11	9.97E-120	1.170559683	0.48	0.14	2.13E-115	Endothelial
LMCD11	2.27E-117	0.707325249	0.294	0.056	4.84E-113	Endothelial
MAST4	6.99E-117	0.729919524	0.344	0.074	1.49E-112	Endothelial
MGLL	2.40E-115	0.807324894	0.3	0.062	5.13E-111	Endothelial
PLEKHA1	2.94E-115	0.723294983	0.336	0.074	6.27E-111	Endothelial
TMSB102	4.75E-114	0.809461312	0.995	0.923	1.01E-109	Endothelial
LMNA1	9.77E-113	1.20120478	0.771	0.384	2.09E-108	Endothelial
KLF91	7.87E-112	0.86029497	0.433	0.118	1.68E-107	Endothelial
LEPROT1	1.27E-111	0.860842787	0.426	0.117	2.70E-107	Endothelial
RCN11	2.00E-110	0.707218037	0.341	0.08	4.28E-106	Endothelial
MSN	3.93E-110	0.929802068	0.606	0.212	8.39E-106	Endothelial
FEZ2	4.00E-110	0.740860613	0.324	0.073	8.54E-106	Endothelial
ARHGEF12	2.10E-109	0.661906808	0.285	0.057	4.48E-105	Endothelial
NFIA1	2.01E-108	0.587730849	0.329	0.072	4.28E-104	Endothelial
PDLIM41	9.69E-108	0.766540779	0.264	0.051	2.07E-103	Endothelial
LDLR1	2.88E-107	0.786257407	0.38	0.097	6.15E-103	Endothelial
VAT1	3.74E-105	0.622172361	0.262	0.051	7.98E-101	Endothelial
CTTN2	1.84E-104	0.689931591	0.312	0.07	3.93E-100	Endothelial
RAC13	2.31E-104	0.997906025	0.812	0.401	4.94E-100	Endothelial

FSTL11	4.20E-104	0.67031096	0.374	0.093	8.97E-100	Endothelial
HIF1A2	7.47E-104	0.918388748	0.498	0.161	1.59E-99	Endothelial
F2R1	3.19E-103	0.561158531	0.341	0.079	6.80E-99	Endothelial
TACC11	1.66E-102	1.001670386	0.562	0.201	3.55E-98	Endothelial
MRPL33	1.76E-102	1.023980614	0.568	0.208	3.76E-98	Endothelial
MARCKSL1	2.52E-102	0.802381932	0.459	0.138	5.38E-98	Endothelial
IER33	8.48E-102	1.040886649	0.645	0.253	1.81E-97	Endothelial
ATOX12	1.38E-100	0.795947956	0.568	0.203	2.95E-96	Endothelial
SPAG91	1.18E-99	0.926261109	0.529	0.184	2.52E-95	Endothelial
CCL21	2.58E-99	1.297743823	0.265	0.055	5.51E-95	Endothelial
SRP14	2.87E-99	1.336665968	0.874	0.58	6.13E-95	Endothelial
TUBB61	2.13E-98	0.633389326	0.253	0.051	4.54E-94	Endothelial
QKI1	2.37E-98	0.735846296	0.367	0.098	5.06E-94	Endothelial
SEC14L1	2.39E-98	0.79723105	0.474	0.147	5.11E-94	Endothelial
ASPH2	1.19E-97	0.70012856	0.373	0.101	2.54E-93	Endothelial
ARL4A	2.92E-96	0.99963021	0.468	0.154	6.23E-92	Endothelial
ACTN41	9.66E-96	0.872542704	0.58	0.215	2.06E-91	Endothelial
GNAQ1	2.70E-95	0.687187524	0.345	0.091	5.76E-91	Endothelial
TSPAN41	5.45E-95	0.587300548	0.288	0.065	1.16E-90	Endothelial
SGK11	1.65E-94	0.825718529	0.376	0.104	3.53E-90	Endothelial
FABP52	5.72E-94	1.209735839	0.473	0.155	1.22E-89	Endothelial
AKAP121	1.28E-92	2.484634616	0.288	0.071	2.73E-88	Endothelial
MTRNR2L8	1.69E-92	0.82348184	0.352	0.096	3.60E-88	Endothelial
MARCKS2	5.45E-92	0.744072558	0.461	0.148	1.16E-87	Endothelial
CTNNA11	7.90E-92	0.676847291	0.348	0.095	1.69E-87	Endothelial
FLOT12	1.13E-91	0.704020404	0.405	0.122	2.40E-87	Endothelial
TSPAN13	2.78E-90	0.497557113	0.253	0.053	5.94E-86	Endothelial
ZEB11	1.45E-89	0.733987495	0.345	0.092	3.10E-85	Endothelial
ROCK2	1.73E-89	0.596000134	0.255	0.055	3.69E-85	Endothelial
PTMA	2.37E-88	0.575466895	0.995	0.965	5.07E-84	Endothelial
HBEGF1	2.44E-88	0.859045399	0.308	0.078	5.20E-84	Endothelial
PEA151	6.10E-88	0.624422655	0.285	0.069	1.30E-83	Endothelial
YWHAH2	1.09E-87	0.820363361	0.527	0.195	2.32E-83	Endothelial
PPP3CA1	1.89E-87	0.694343377	0.394	0.117	4.04E-83	Endothelial
LHFPL61	4.21E-86	0.422429362	0.315	0.078	8.99E-82	Endothelial
TPM42	2.14E-85	0.928509955	0.694	0.319	4.57E-81	Endothelial
PHACTR4	6.91E-85	0.568274136	0.326	0.086	1.48E-80	Endothelial
ATP1A1	7.36E-85	0.89328071	0.545	0.211	1.57E-80	Endothelial
TNFAIP22	3.94E-84	0.642746949	0.359	0.102	8.41E-80	Endothelial
VGLL41	9.41E-84	0.645052826	0.28	0.068	2.01E-79	Endothelial
NFIC1	1.50E-83	0.652939681	0.38	0.113	3.20E-79	Endothelial
WARS	2.27E-83	0.696649745	0.289	0.073	4.85E-79	Endothelial
ITGB11	2.49E-83	0.835083021	0.733	0.344	5.32E-79	Endothelial

ANXA23	3.71E-83	0.813085121	0.783	0.391	7.92E-79	Endothelial
RNF145	1.17E-82	0.874929159	0.585	0.24	2.49E-78	Endothelial
TCEAL81	3.06E-82	0.603843111	0.373	0.112	6.54E-78	Endothelial
ARL21	4.76E-82	0.622080721	0.318	0.087	1.02E-77	Endothelial
YWHAE2	3.99E-81	0.806091301	0.671	0.3	8.51E-77	Endothelial
AP1S21	6.02E-81	0.672854053	0.368	0.109	1.29E-76	Endothelial
CTNNB12	9.13E-80	0.80345882	0.508	0.189	1.95E-75	Endothelial
CCDC69	1.30E-79	0.520301604	0.274	0.066	2.77E-75	Endothelial
PRMT1	5.03E-79	0.638267626	0.311	0.085	1.07E-74	Endothelial
TAGLN21	6.13E-79	0.86214956	0.698	0.338	1.31E-74	Endothelial
TMEM173	2.43E-78	0.527525637	0.335	0.093	5.20E-74	Endothelial
BEX31	9.43E-78	0.713325307	0.388	0.122	2.01E-73	Endothelial
ENTPD1	1.20E-77	0.515253649	0.38	0.115	2.56E-73	Endothelial
UGCG1	6.37E-77	0.777765255	0.382	0.123	1.36E-72	Endothelial
FOS1	9.87E-77	0.933452686	0.835	0.485	2.11E-72	Endothelial
TPM31	4.25E-76	0.839741488	0.747	0.403	9.08E-72	Endothelial
MBNL2	4.29E-76	0.599337808	0.288	0.076	9.15E-72	Endothelial
DAB22	4.40E-76	0.554189135	0.355	0.105	9.39E-72	Endothelial
VAMP3	8.54E-76	0.552204049	0.274	0.071	1.82E-71	Endothelial
GIMAP7	2.32E-75	0.567330834	0.303	0.079	4.94E-71	Endothelial
PGM2L1	2.72E-75	0.485687256	0.306	0.083	5.81E-71	Endothelial
DAAM11	3.74E-75	0.668082109	0.327	0.095	7.99E-71	Endothelial
PICALM	6.01E-75	0.540600671	0.297	0.081	1.28E-70	Endothelial
CAPNS12	7.62E-75	0.627280576	0.415	0.142	1.63E-70	Endothelial
BNIP2	8.30E-75	0.464931051	0.324	0.093	1.77E-70	Endothelial
ANKRD281	9.47E-75	0.58912331	0.542	0.209	2.02E-70	Endothelial
PMP222	1.92E-74	0.563864674	0.365	0.111	4.10E-70	Endothelial
TNFSF10	5.93E-74	0.611542171	0.283	0.075	1.27E-69	Endothelial
HSPB13	4.05E-73	0.585609977	0.844	0.422	8.64E-69	Endothelial
LAPTM4A1	7.90E-73	0.646834409	0.594	0.244	1.69E-68	Endothelial
MIDN	1.42E-71	0.770102573	0.562	0.237	3.03E-67	Endothelial
GABARAPL	2.69E-71	0.665463947	0.533	0.213	5.74E-67	Endothelial
FOSB1	5.17E-71	0.820010849	0.792	0.437	1.10E-66	Endothelial
CAMTA12	1.22E-70	0.652518756	0.405	0.142	2.60E-66	Endothelial
EIF2S21	2.59E-70	0.771540317	0.635	0.297	5.53E-66	Endothelial
BCAP29	3.96E-70	0.606356973	0.261	0.068	8.45E-66	Endothelial
ZBTB201	4.62E-69	0.518761022	0.5	0.186	9.87E-65	Endothelial
DNAJB41	5.91E-69	0.536737649	0.306	0.089	1.26E-64	Endothelial
CDC37	7.78E-69	0.709797762	0.52	0.214	1.66E-64	Endothelial
SERPINH11	1.16E-68	0.533254152	0.362	0.116	2.47E-64	Endothelial
UTRN	1.16E-68	0.562464177	0.382	0.125	2.47E-64	Endothelial
MAFF1	1.93E-68	0.530034196	0.427	0.151	4.11E-64	Endothelial
ELMO1	3.51E-68	0.405855786	0.273	0.072	7.49E-64	Endothelial

JUNB1	6.44E-68	0.950866203	0.83	0.558	1.38E-63	Endothelial
SERTAD11	6.60E-68	0.716629613	0.429	0.157	1.41E-63	Endothelial
TMOD3	7.99E-68	0.568314822	0.385	0.131	1.71E-63	Endothelial
SNHG7	1.18E-67	0.568945448	0.389	0.133	2.52E-63	Endothelial
ZBTB161	4.73E-67	0.463698452	0.3	0.085	1.01E-62	Endothelial
ELF2	2.39E-66	0.439597274	0.323	0.098	5.11E-62	Endothelial
S100A103	6.93E-66	0.71547333	0.858	0.538	1.48E-61	Endothelial
GRN2	9.89E-66	0.640461521	0.409	0.147	2.11E-61	Endothelial
SLC2A31	1.98E-65	0.672764572	0.539	0.224	4.22E-61	Endothelial
PLSCR11	2.91E-65	0.539718117	0.297	0.089	6.22E-61	Endothelial
NAA10	3.17E-64	0.589648253	0.362	0.125	6.77E-60	Endothelial
ABLIM1	3.72E-64	0.58088797	0.303	0.091	7.94E-60	Endothelial
RAB5C1	2.78E-63	0.499491733	0.43	0.161	5.93E-59	Endothelial
SLK	1.66E-62	0.433987906	0.277	0.08	3.53E-58	Endothelial
HLA-E1	1.81E-62	0.724153448	0.891	0.654	3.86E-58	Endothelial
NPC22	2.64E-62	0.707545188	0.573	0.258	5.64E-58	Endothelial
NFKBIA2	4.53E-62	0.818222421	0.786	0.478	9.68E-58	Endothelial
GUK11	1.88E-61	0.760321788	0.759	0.448	4.01E-57	Endothelial
SLC25A37	2.18E-61	0.562856391	0.306	0.096	4.66E-57	Endothelial
XAF1	2.98E-61	0.571427016	0.295	0.091	6.36E-57	Endothelial
EBPL	6.18E-61	0.50565771	0.274	0.082	1.32E-56	Endothelial
TIMP12	1.44E-60	0.362806991	0.668	0.3	3.07E-56	Endothelial
PSMB51	1.52E-60	0.561135167	0.312	0.102	3.24E-56	Endothelial
FILIP1L1	3.28E-60	0.525331537	0.329	0.108	7.00E-56	Endothelial
RPL36A	4.63E-60	0.731393633	0.847	0.594	9.87E-56	Endothelial
TMEM30A	9.11E-60	0.48693792	0.267	0.079	1.95E-55	Endothelial
EID11	1.20E-59	0.62905495	0.591	0.269	2.56E-55	Endothelial
PHACTR21	5.93E-59	0.465996273	0.358	0.124	1.27E-54	Endothelial
FCGRT2	1.13E-58	0.607646667	0.361	0.129	2.41E-54	Endothelial
RPS281	1.44E-58	0.5166691	0.988	0.96	3.08E-54	Endothelial
MYL12B1	1.71E-58	0.733309848	0.758	0.441	3.66E-54	Endothelial
RTN42	2.04E-58	0.62603373	0.574	0.266	4.36E-54	Endothelial
MACF1	6.65E-58	0.545715724	0.426	0.164	1.42E-53	Endothelial
PIM31	7.55E-58	0.577388644	0.433	0.171	1.61E-53	Endothelial
C5orf24	1.46E-57	0.547784366	0.3	0.097	3.12E-53	Endothelial
VAPA1	2.20E-57	0.629843102	0.567	0.262	4.70E-53	Endothelial
SERPINB63	2.48E-57	0.564299436	0.335	0.117	5.29E-53	Endothelial
METAP21	3.37E-57	0.560764147	0.611	0.289	7.20E-53	Endothelial
SYPL11	3.46E-57	0.568628331	0.324	0.111	7.40E-53	Endothelial
HNRNPM	7.03E-57	0.570874874	0.589	0.275	1.50E-52	Endothelial
RRBP12	1.43E-56	0.54311654	0.609	0.28	3.06E-52	Endothelial
RALGDS	4.45E-56	0.395585479	0.303	0.097	9.50E-52	Endothelial
EGR11	4.59E-56	0.562936951	0.517	0.223	9.79E-52	Endothelial

DDX21	4.86E-56	0.778083595	0.685	0.359	1.04E-51	Endothelial
GADD45B2	1.25E-55	0.590004862	0.55	0.248	2.67E-51	Endothelial
CBX3	1.34E-55	0.609050529	0.642	0.326	2.86E-51	Endothelial
IFITM11	1.72E-55	0.979092806	0.344	0.126	3.68E-51	Endothelial
BAZ2B	2.30E-55	0.499423854	0.253	0.075	4.92E-51	Endothelial
SYNJ2	3.94E-55	0.449103629	0.261	0.078	8.40E-51	Endothelial
CSNK1A1	5.74E-55	0.606385778	0.565	0.264	1.23E-50	Endothelial
CRK	7.03E-55	0.407651628	0.305	0.101	1.50E-50	Endothelial
RBMS11	9.95E-55	0.50045771	0.415	0.161	2.12E-50	Endothelial
CHD91	3.98E-54	0.46557714	0.32	0.11	8.49E-50	Endothelial
ZFP361	1.55E-53	0.875559609	0.73	0.45	3.30E-49	Endothelial
BRI33	3.19E-53	0.537201877	0.476	0.203	6.81E-49	Endothelial
SKAP21	5.93E-53	0.407203733	0.273	0.087	1.26E-48	Endothelial
MYH9	7.27E-53	0.526790214	0.539	0.243	1.55E-48	Endothelial
AHR	1.10E-52	0.439406593	0.259	0.081	2.35E-48	Endothelial
SH3GLB1	1.24E-52	0.508343587	0.5	0.216	2.65E-48	Endothelial
EPS82	4.19E-52	0.520646588	0.252	0.078	8.94E-48	Endothelial
FNDC3B2	5.43E-52	0.516629451	0.292	0.099	1.16E-47	Endothelial
PFDN1	8.32E-52	0.522698276	0.339	0.124	1.78E-47	Endothelial
YWHAG2	1.18E-51	0.559014613	0.359	0.138	2.53E-47	Endothelial
EIF4G1	1.47E-51	0.41599939	0.303	0.104	3.15E-47	Endothelial
CD633	3.98E-51	0.354948269	0.764	0.388	8.50E-47	Endothelial
GNAS	2.88E-50	0.656878304	0.656	0.346	6.15E-46	Endothelial
POLR2L2	3.70E-50	0.609528671	0.726	0.407	7.91E-46	Endothelial
GNB11	3.97E-50	0.518844852	0.435	0.184	8.47E-46	Endothelial
ARRDC3	4.42E-50	0.494804665	0.274	0.091	9.43E-46	Endothelial
BCAP311	5.89E-50	0.473350393	0.327	0.12	1.26E-45	Endothelial
HSP90B12	6.07E-50	0.59805531	0.698	0.372	1.30E-45	Endothelial
MYL12A	3.22E-49	0.589637097	0.765	0.449	6.88E-45	Endothelial
RALBP11	4.33E-49	0.413486113	0.4	0.162	9.25E-45	Endothelial
GALNT1	4.38E-49	0.436772565	0.28	0.095	9.34E-45	Endothelial
ZFP36L12	4.42E-49	0.615438068	0.661	0.361	9.43E-45	Endothelial
MYADM1	5.50E-49	0.599950422	0.526	0.247	1.17E-44	Endothelial
CANX2	6.03E-49	0.54248387	0.453	0.199	1.29E-44	Endothelial
NSRP11	7.29E-49	0.589886139	0.345	0.131	1.56E-44	Endothelial
RPL37A1	1.42E-48	0.445922755	0.974	0.94	3.04E-44	Endothelial
SEM12	7.36E-48	0.525211967	0.579	0.287	1.57E-43	Endothelial
ARPC1B1	8.40E-48	0.559665602	0.624	0.326	1.79E-43	Endothelial
CAPZA2	1.33E-47	0.517431826	0.405	0.169	2.85E-43	Endothelial
PLEC1	1.51E-47	0.413028359	0.327	0.121	3.23E-43	Endothelial
DYNC1LI21	3.22E-47	0.429335531	0.3	0.107	6.87E-43	Endothelial
SNX32	3.46E-47	0.645996894	0.555	0.274	7.38E-43	Endothelial
LPP1	4.77E-47	0.427191954	0.385	0.154	1.02E-42	Endothelial

UPF3A	5.13E-47	0.320613139	0.306	0.11	1.10E-42	Endothelial
C8orf33	9.36E-47	0.516757758	0.28	0.098	2.00E-42	Endothelial
APLP22	1.03E-46	0.567935861	0.456	0.202	2.20E-42	Endothelial
TSPO2	1.50E-46	0.530342567	0.579	0.28	3.21E-42	Endothelial
SARS1	1.87E-46	0.478433916	0.364	0.144	3.99E-42	Endothelial
PITPNB	1.90E-46	0.397668504	0.302	0.109	4.06E-42	Endothelial
NBDY	2.96E-46	0.403538594	0.347	0.135	6.33E-42	Endothelial
NFAT5	3.04E-46	0.398613292	0.32	0.117	6.49E-42	Endothelial
CDKN1A2	4.71E-46	0.696370312	0.498	0.238	1.01E-41	Endothelial
IRF2BP2	5.82E-46	0.465701553	0.371	0.15	1.24E-41	Endothelial
EIF4A11	5.84E-46	0.423839712	0.535	0.254	1.25E-41	Endothelial
PWP1	9.12E-46	0.386223583	0.314	0.116	1.95E-41	Endothelial
CPEB41	2.23E-45	0.422491713	0.329	0.127	4.76E-41	Endothelial
ITM2A	4.42E-45	0.328426503	0.35	0.131	9.44E-41	Endothelial
MT-ATP81	7.62E-45	0.438285492	0.327	0.125	1.63E-40	Endothelial
HSP90AB1	2.17E-44	0.465022742	0.9	0.629	4.62E-40	Endothelial
PPP1R15A	2.21E-44	0.543053061	0.682	0.387	4.72E-40	Endothelial
ELOC1	2.93E-44	0.475048571	0.45	0.202	6.25E-40	Endothelial
SBDS1	3.49E-44	0.375904406	0.373	0.151	7.46E-40	Endothelial
PUM1	3.66E-44	0.428435301	0.344	0.136	7.81E-40	Endothelial
ENY2	4.50E-44	0.44661913	0.518	0.253	9.61E-40	Endothelial
PELI1	5.10E-44	0.450093192	0.274	0.096	1.09E-39	Endothelial
SLC38A21	1.45E-43	0.471688638	0.556	0.278	3.09E-39	Endothelial
MT2A2	2.69E-43	0.734439091	0.714	0.434	5.74E-39	Endothelial
NDUFB13	3.52E-43	0.481867894	0.626	0.322	7.52E-39	Endothelial
PSMC11	3.75E-43	0.478312113	0.403	0.175	8.01E-39	Endothelial
JMJD1C	4.75E-43	0.462197975	0.497	0.232	1.01E-38	Endothelial
RERE	6.02E-43	0.311019859	0.306	0.114	1.29E-38	Endothelial
TUBB2	1.01E-42	0.472389898	0.505	0.237	2.15E-38	Endothelial
CCDC251	1.05E-42	0.37693806	0.273	0.098	2.24E-38	Endothelial
CLEC2B	1.33E-42	0.446319614	0.405	0.17	2.85E-38	Endothelial
SEC621	1.64E-42	0.53453827	0.647	0.349	3.51E-38	Endothelial
NUCKS11	1.77E-42	0.434172555	0.602	0.301	3.78E-38	Endothelial
CD991	1.82E-42	0.531569511	0.615	0.313	3.87E-38	Endothelial
ARPC5L	2.01E-42	0.368463902	0.355	0.144	4.30E-38	Endothelial
CALU1	5.24E-42	0.419461293	0.341	0.136	1.12E-37	Endothelial
GNG52	5.54E-42	0.509745338	0.561	0.284	1.18E-37	Endothelial
NOP102	5.59E-42	0.443530212	0.535	0.261	1.19E-37	Endothelial
BTBD7	7.78E-42	0.320972356	0.252	0.086	1.66E-37	Endothelial
SRSF4	9.96E-42	0.426022727	0.414	0.183	2.13E-37	Endothelial
MAP3K13	1.77E-41	0.404061691	0.255	0.09	3.77E-37	Endothelial
HMGN3	1.87E-41	0.417193595	0.362	0.151	4.00E-37	Endothelial
XIST1	2.60E-41	0.558916154	0.55	0.287	5.55E-37	Endothelial

TAX1BP31	3.03E-41	0.372725552	0.252	0.089	6.46E-37	Endothelial
CEBPD2	5.53E-41	0.368451719	0.439	0.197	1.18E-36	Endothelial
PFDN4	6.61E-41	0.398340681	0.285	0.106	1.41E-36	Endothelial
PTPRE1	7.17E-41	0.441555523	0.306	0.117	1.53E-36	Endothelial
PSMD14	7.39E-41	0.406132026	0.352	0.147	1.58E-36	Endothelial
PYURF1	8.39E-41	0.412728927	0.359	0.151	1.79E-36	Endothelial
CFDP11	8.67E-41	0.460837238	0.389	0.17	1.85E-36	Endothelial
MTPN1	1.99E-40	0.38012673	0.458	0.213	4.25E-36	Endothelial
FIS12	2.16E-39	0.442661241	0.438	0.206	4.62E-35	Endothelial
UBE2R2	2.21E-39	0.329640563	0.289	0.111	4.71E-35	Endothelial
NMT11	2.27E-39	0.328761764	0.405	0.177	4.84E-35	Endothelial
SVIP	2.81E-39	0.26023699	0.374	0.156	6.00E-35	Endothelial
KLF61	5.36E-39	0.669838584	0.767	0.542	1.14E-34	Endothelial
CLTA3	5.77E-39	0.440680665	0.485	0.235	1.23E-34	Endothelial
PTBP1	6.13E-39	0.434237217	0.429	0.197	1.31E-34	Endothelial
SREK1	9.00E-39	0.291711822	0.362	0.152	1.92E-34	Endothelial
REXO21	9.10E-39	0.406994751	0.321	0.129	1.94E-34	Endothelial
NUCB11	1.99E-38	0.389135842	0.324	0.133	4.24E-34	Endothelial
TSPAN14	2.46E-38	0.317235222	0.271	0.099	5.24E-34	Endothelial
SERINC3	3.09E-38	0.27257876	0.323	0.131	6.60E-34	Endothelial
CRTAP1	3.15E-38	0.371389221	0.268	0.102	6.73E-34	Endothelial
NDUFA121	3.96E-38	0.416534935	0.362	0.157	8.45E-34	Endothelial
TES	1.00E-37	0.351291903	0.421	0.19	2.14E-33	Endothelial
ZNF207	1.21E-37	0.261496008	0.408	0.181	2.58E-33	Endothelial
CXCL22	1.75E-37	0.568703385	0.308	0.124	3.74E-33	Endothelial
MAP4K4	2.00E-37	0.313852644	0.283	0.109	4.28E-33	Endothelial
CYTH1	2.12E-37	0.38680384	0.32	0.129	4.53E-33	Endothelial
ZNHIT12	4.95E-37	0.376012938	0.455	0.219	1.06E-32	Endothelial
SHOC2	5.44E-37	0.307836901	0.345	0.145	1.16E-32	Endothelial
LAP31	5.72E-37	0.429871111	0.294	0.118	1.22E-32	Endothelial
SETD5	6.98E-37	0.271747204	0.297	0.117	1.49E-32	Endothelial
SNHG8	1.17E-36	0.378735663	0.548	0.282	2.49E-32	Endothelial
EIF5B	2.05E-36	0.425932733	0.621	0.337	4.38E-32	Endothelial
AP2S13	5.04E-36	0.418490982	0.455	0.22	1.08E-31	Endothelial
NEAT12	5.70E-36	0.514430741	0.968	0.841	1.22E-31	Endothelial
SELENOM1	5.92E-36	0.308315794	0.406	0.181	1.26E-31	Endothelial
CHIC2	6.46E-36	0.410866393	0.253	0.095	1.38E-31	Endothelial
HNRNPD	6.65E-36	0.34164125	0.397	0.182	1.42E-31	Endothelial
MT-ND4L1	6.99E-36	0.384365418	0.609	0.337	1.49E-31	Endothelial
SEC11A1	1.15E-35	0.444714829	0.509	0.263	2.45E-31	Endothelial
POMP2	1.88E-35	0.489718215	0.641	0.366	4.02E-31	Endothelial
MORF4L11	2.19E-35	0.479895885	0.676	0.395	4.67E-31	Endothelial
MAP1LC3E	2.24E-35	0.424906374	0.508	0.262	4.78E-31	Endothelial

IPO7	2.38E-35	0.307669617	0.265	0.102	5.08E-31	Endothelial
PFDN2	2.39E-35	0.374803191	0.38	0.172	5.11E-31	Endothelial
MAP41	2.94E-35	0.439646504	0.295	0.121	6.28E-31	Endothelial
DUT	4.16E-35	0.369002831	0.327	0.14	8.87E-31	Endothelial
RAB11B	6.73E-35	0.34185449	0.262	0.102	1.44E-30	Endothelial
MIR4435-2	7.78E-35	0.734894528	0.364	0.17	1.66E-30	Endothelial
PPA12	7.97E-35	0.481281069	0.429	0.21	1.70E-30	Endothelial
EI24	1.00E-34	0.357407239	0.25	0.097	2.13E-30	Endothelial
TSC22D2	1.85E-34	0.304953978	0.291	0.118	3.95E-30	Endothelial
21-Sep	1.88E-34	0.366616078	0.385	0.18	4.01E-30	Endothelial
G3BP1	2.63E-34	0.284783758	0.309	0.128	5.60E-30	Endothelial
AP2B1	2.89E-34	0.394805468	0.441	0.214	6.16E-30	Endothelial
ATP5PF2	3.43E-34	0.383495632	0.541	0.287	7.31E-30	Endothelial
SET1	3.66E-34	0.423559456	0.644	0.368	7.82E-30	Endothelial
PPP1R14B1	7.58E-34	0.39538948	0.27	0.109	1.62E-29	Endothelial
NDUFC22	9.01E-34	0.345194688	0.397	0.187	1.92E-29	Endothelial
CHMP2A2	1.18E-33	0.322080762	0.397	0.187	2.52E-29	Endothelial
RAB7A2	1.20E-33	0.417342906	0.359	0.164	2.57E-29	Endothelial
INSIG11	1.28E-33	0.427343413	0.335	0.145	2.72E-29	Endothelial
ARGLU11	1.59E-33	0.427349081	0.674	0.396	3.40E-29	Endothelial
CNIH1	1.80E-33	0.279197727	0.289	0.119	3.84E-29	Endothelial
AFF41	2.01E-33	0.322690418	0.356	0.161	4.29E-29	Endothelial
KPNA4	3.21E-33	0.306230602	0.274	0.111	6.86E-29	Endothelial
DDX11	3.80E-33	0.253721165	0.268	0.107	8.10E-29	Endothelial
RHOA2	4.27E-33	0.432164602	0.665	0.388	9.12E-29	Endothelial
RPS17	4.29E-33	0.432292598	0.892	0.743	9.17E-29	Endothelial
SREK1IP1	4.40E-33	0.302391641	0.283	0.117	9.40E-29	Endothelial
HP1BP31	4.66E-33	0.421463112	0.453	0.226	9.94E-29	Endothelial
HSBP13	5.15E-33	0.37795694	0.382	0.179	1.10E-28	Endothelial
PAFAH1B2	7.74E-33	0.256215834	0.271	0.109	1.65E-28	Endothelial
IER3IP1	1.16E-32	0.342696441	0.274	0.113	2.48E-28	Endothelial
TBCA2	1.34E-32	0.382815798	0.676	0.391	2.86E-28	Endothelial
LAMTOR51	1.75E-32	0.353844129	0.456	0.228	3.73E-28	Endothelial
KRAS	1.84E-32	0.281693345	0.323	0.14	3.93E-28	Endothelial
TAF11	2.59E-32	0.285360527	0.265	0.106	5.52E-28	Endothelial
COMT1	4.40E-32	0.380952487	0.25	0.1	9.39E-28	Endothelial
SNRPE1	4.47E-32	0.260797609	0.418	0.2	9.54E-28	Endothelial
GTF2I	1.04E-31	0.38056117	0.355	0.163	2.22E-27	Endothelial
SERBP1	1.07E-31	0.436797197	0.721	0.445	2.28E-27	Endothelial
LIMA12	1.99E-31	0.365058002	0.317	0.14	4.25E-27	Endothelial
HNRNPH1	2.19E-31	0.290582572	0.558	0.301	4.68E-27	Endothelial
MTCH11	2.79E-31	0.334097251	0.383	0.183	5.95E-27	Endothelial
EIF3J	3.55E-31	0.352344212	0.564	0.303	7.57E-27	Endothelial

COPS91	4.15E-31	0.343826727	0.511	0.269	8.86E-27	Endothelial
TRIM44	4.43E-31	0.307992613	0.262	0.107	9.45E-27	Endothelial
CREBRF	6.09E-31	0.295624292	0.368	0.171	1.30E-26	Endothelial
PDIA62	7.39E-31	0.36270463	0.392	0.191	1.58E-26	Endothelial
TMCO11	9.07E-31	0.373257794	0.371	0.177	1.94E-26	Endothelial
IER21	1.21E-30	0.398112262	0.588	0.324	2.58E-26	Endothelial
PDIA41	1.49E-30	0.277041369	0.282	0.12	3.18E-26	Endothelial
FDPS	1.84E-30	0.318806789	0.273	0.115	3.93E-26	Endothelial
RBM17	1.96E-30	0.317346591	0.382	0.186	4.19E-26	Endothelial
PRMT2	2.75E-30	0.292492733	0.465	0.238	5.87E-26	Endothelial
XRN2	2.97E-30	0.260461412	0.344	0.158	6.35E-26	Endothelial
WDR83OS	3.90E-30	0.367977241	0.409	0.204	8.33E-26	Endothelial
BZW1	4.17E-30	0.283481783	0.506	0.266	8.90E-26	Endothelial
SOD22	4.39E-30	0.33705194	0.426	0.213	9.36E-26	Endothelial
DUSP12	4.80E-30	0.317758918	0.685	0.405	1.02E-25	Endothelial
RTF1	7.88E-30	0.337854946	0.502	0.266	1.68E-25	Endothelial
GABARAP	1.27E-29	0.279699412	0.256	0.105	2.71E-25	Endothelial
SLIRP1	1.41E-29	0.363469416	0.4	0.198	3.01E-25	Endothelial
LAMP12	1.75E-29	0.313392717	0.373	0.178	3.73E-25	Endothelial
PCMTD1	1.93E-29	0.291709182	0.258	0.108	4.13E-25	Endothelial
PA2G41	3.12E-29	0.279042842	0.517	0.275	6.66E-25	Endothelial
ZNF428	3.56E-29	0.298900673	0.25	0.102	7.59E-25	Endothelial
CSRNP11	3.97E-29	0.289325131	0.336	0.155	8.47E-25	Endothelial
VMP12	4.17E-29	0.41508575	0.485	0.259	8.91E-25	Endothelial
TMEM219	5.11E-29	0.303016238	0.317	0.146	1.09E-24	Endothelial
NHP21	6.12E-29	0.356160214	0.309	0.141	1.31E-24	Endothelial
NUFIP2	7.00E-29	0.265411559	0.348	0.164	1.49E-24	Endothelial
ARF1	9.12E-29	0.313355027	0.505	0.274	1.95E-24	Endothelial
DPM3	1.09E-28	0.305358933	0.255	0.107	2.33E-24	Endothelial
RHEB2	1.15E-28	0.303612775	0.383	0.188	2.46E-24	Endothelial
TMBIM42	1.37E-28	0.289023402	0.371	0.178	2.93E-24	Endothelial
GNAI3	1.84E-28	0.309376316	0.294	0.131	3.92E-24	Endothelial
WDR45B1	2.56E-28	0.299420551	0.285	0.126	5.46E-24	Endothelial
ACAP2	6.14E-28	0.277349244	0.268	0.116	1.31E-23	Endothelial
NME11	7.12E-28	0.376893067	0.262	0.115	1.52E-23	Endothelial
GADD45G	1.31E-27	0.33562129	0.42	0.216	2.79E-23	Endothelial
HNRNPK	1.66E-27	0.325320332	0.585	0.34	3.54E-23	Endothelial
SDCBP1	1.76E-27	0.330889139	0.548	0.31	3.75E-23	Endothelial
GNB22	4.77E-27	0.298538463	0.306	0.141	1.02E-22	Endothelial
CCNL1	4.95E-27	0.441582066	0.62	0.381	1.06E-22	Endothelial
TMEM14C	6.21E-27	0.268470268	0.305	0.141	1.33E-22	Endothelial
NEDD81	3.26E-26	0.333299393	0.524	0.292	6.96E-22	Endothelial
COX171	7.30E-26	0.373732634	0.436	0.232	1.56E-21	Endothelial

PRKAR1A	8.78E-26	0.321093467	0.323	0.156	1.87E-21	Endothelial
TMEM2581	8.87E-26	0.267782533	0.609	0.353	1.89E-21	Endothelial
RAD23A	9.48E-26	0.262994419	0.35	0.173	2.02E-21	Endothelial
TMEM50A	1.22E-25	0.257461107	0.456	0.242	2.61E-21	Endothelial
ATF41	1.27E-25	0.314027568	0.652	0.388	2.72E-21	Endothelial
BSG2	2.57E-25	0.292092738	0.432	0.229	5.48E-21	Endothelial
MTIF3	2.72E-25	0.287340679	0.274	0.126	5.80E-21	Endothelial
SNHG9	4.01E-25	0.270362863	0.294	0.137	8.55E-21	Endothelial
NBEAL1	5.66E-25	0.269002215	0.302	0.143	1.21E-20	Endothelial
WASF21	7.93E-25	0.252164918	0.429	0.228	1.69E-20	Endothelial
CHURC11	9.36E-25	0.258852032	0.353	0.177	2.00E-20	Endothelial
NR4A11	1.05E-24	0.300308338	0.455	0.247	2.24E-20	Endothelial
AHCYL11	1.08E-24	0.271933133	0.277	0.129	2.30E-20	Endothelial
NDUFC12	1.25E-24	0.340632461	0.348	0.176	2.67E-20	Endothelial
POLE4	1.30E-24	0.252435576	0.267	0.122	2.77E-20	Endothelial
KDELR12	1.59E-24	0.253004461	0.314	0.153	3.40E-20	Endothelial
TMEM147	1.60E-24	0.302482834	0.329	0.164	3.42E-20	Endothelial
HNRNPA3	3.97E-24	0.253540503	0.512	0.285	8.47E-20	Endothelial
RPL34	5.40E-24	0.307933711	0.983	0.961	1.15E-19	Endothelial
GOLIM41	5.49E-24	0.306158273	0.261	0.12	1.17E-19	Endothelial
WSB11	6.88E-24	0.344933193	0.505	0.285	1.47E-19	Endothelial
MRPL521	8.90E-24	0.285128485	0.336	0.17	1.90E-19	Endothelial
MINOS12	1.03E-23	0.293518864	0.406	0.217	2.20E-19	Endothelial
SPCS31	1.21E-23	0.264625649	0.308	0.149	2.58E-19	Endothelial
AP2M11	1.55E-23	0.268797	0.367	0.188	3.32E-19	Endothelial
NDUFA112	1.79E-23	0.340570541	0.464	0.262	3.83E-19	Endothelial
LAMTOR22	2.54E-23	0.320320402	0.286	0.14	5.42E-19	Endothelial
GSTO12	2.61E-23	0.296826972	0.356	0.184	5.56E-19	Endothelial
C4orf482	3.44E-23	0.350738208	0.364	0.19	7.35E-19	Endothelial
VCP	4.86E-23	0.285114922	0.27	0.127	1.04E-18	Endothelial
LIMS11	5.02E-23	0.305532194	0.379	0.199	1.07E-18	Endothelial
PDIA31	7.18E-23	0.251939438	0.397	0.21	1.53E-18	Endothelial
MGST32	1.40E-22	0.324047528	0.383	0.203	2.99E-18	Endothelial
VKORC11	1.44E-22	0.367716864	0.289	0.142	3.07E-18	Endothelial
TBCB	1.61E-22	0.289502765	0.291	0.141	3.43E-18	Endothelial
TMED91	1.70E-22	0.326630722	0.326	0.167	3.63E-18	Endothelial
PDCD6	1.77E-22	0.256846725	0.265	0.125	3.78E-18	Endothelial
NDUFB21	2.19E-22	0.274733068	0.553	0.323	4.68E-18	Endothelial
PSMB4	2.41E-22	0.265300198	0.297	0.146	5.14E-18	Endothelial
CHMP4B3	2.72E-22	0.308243797	0.38	0.206	5.80E-18	Endothelial
CSDE1	3.29E-22	0.296263179	0.439	0.249	7.03E-18	Endothelial
RPL17	1.07E-21	0.318902913	0.742	0.516	2.29E-17	Endothelial
MRPS212	1.43E-21	0.280914625	0.415	0.229	3.06E-17	Endothelial

MLEC1	2.59E-21	0.322084103	0.294	0.149	5.53E-17	Endothelial
MYL62	9.43E-21	0.332209422	0.889	0.678	2.01E-16	Endothelial
CDC421	1.23E-20	0.253947048	0.635	0.4	2.63E-16	Endothelial
GADD45A1	2.29E-20	0.428906605	0.329	0.173	4.88E-16	Endothelial
PET100	2.70E-20	0.319177836	0.383	0.209	5.77E-16	Endothelial
SERF21	5.73E-20	0.323650774	0.914	0.755	1.22E-15	Endothelial
RGCC1	1.18E-19	0.476076212	0.555	0.356	2.51E-15	Endothelial
CHD4	3.16E-19	0.26240707	0.315	0.166	6.75E-15	Endothelial
UQCR111	4.57E-19	0.27927712	0.68	0.45	9.75E-15	Endothelial
ELOB2	1.42E-18	0.292993601	0.698	0.459	3.03E-14	Endothelial
PPIA1	2.60E-18	0.346895467	0.809	0.606	5.56E-14	Endothelial
PSMA72	7.09E-18	0.259296511	0.708	0.47	1.51E-13	Endothelial
H3F3A1	7.43E-18	0.303162519	0.871	0.704	1.59E-13	Endothelial
SDF41	3.62E-17	0.256823091	0.262	0.137	7.72E-13	Endothelial
MT-ND51	1.10E-16	0.352607559	0.773	0.618	2.35E-12	Endothelial
YBX11	7.35E-16	0.256502432	0.789	0.592	1.57E-11	Endothelial
IFI62	1.02E-14	0.315923328	0.326	0.193	2.17E-10	Endothelial
BTF3	1.42E-13	0.264884043	0.786	0.608	3.03E-09	Endothelial
UBC1	3.46E-10	0.263657718	0.867	0.691	7.38E-06	Endothelial
GNLY	0	4.054964927	0.98	0.089	0	Nkcell
FGFBP2	0	3.625024394	0.817	0.011	0	Nkcell
NKG71	0	3.569495039	0.997	0.162	0	Nkcell
PRF1	0	3.116372453	0.772	0.047	0	Nkcell
GZMB	0	3.066669299	0.967	0.134	0	Nkcell
KLRD1	0	2.966536236	0.828	0.071	0	Nkcell
KLRF1	0	2.709492825	0.597	0.008	0	Nkcell
CST71	0	2.674653586	0.873	0.145	0	Nkcell
FCGR3A1	0	2.635038334	0.628	0.039	0	Nkcell
GZMH	0	2.571845804	0.711	0.069	0	Nkcell
CTSW	0	2.436360637	0.595	0.037	0	Nkcell
CCL42	0	2.434206434	0.825	0.187	0	Nkcell
CD247	0	2.16477684	0.648	0.095	0	Nkcell
KLRB11	7.74E-275	1.784754011	0.717	0.151	1.65E-270	Nkcell
TYROBP1	6.61E-261	1.453655174	0.719	0.152	1.41E-256	Nkcell
SPON21	5.48E-246	1.968647142	0.569	0.112	1.17E-241	Nkcell
CCL51	1.30E-241	1.446404883	0.896	0.254	2.78E-237	Nkcell
GZMA	3.80E-240	1.557008788	0.64	0.129	8.11E-236	Nkcell
CHST12	1.08E-233	1.815237201	0.498	0.089	2.32E-229	Nkcell
PLAC81	6.53E-227	1.691291342	0.446	0.072	1.39E-222	Nkcell
HLA-B1	2.79E-222	1.396383012	0.995	0.854	5.96E-218	Nkcell
CCL31	7.69E-222	1.814053745	0.476	0.083	1.64E-217	Nkcell
CLIC3	2.05E-215	1.585207877	0.312	0.035	4.37E-211	Nkcell
SYTL31	3.75E-200	1.670575351	0.755	0.237	7.99E-196	Nkcell

CCL4L21	2.87E-199	2.323962616	0.484	0.094	6.14E-195	Nkcell
TRDC	5.64E-190	1.311623361	0.264	0.027	1.20E-185	Nkcell
PLEKHA2	1.96E-186	1.739276452	0.605	0.167	4.17E-182	Nkcell
AKNA1	4.75E-180	1.508273913	0.623	0.168	1.01E-175	Nkcell
CREM1	1.38E-178	1.754503644	0.879	0.453	2.95E-174	Nkcell
ARL4C1	1.44E-176	1.650147805	0.716	0.245	3.08E-172	Nkcell
GK5	2.63E-175	1.407918734	0.263	0.031	5.61E-171	Nkcell
HOPX	3.17E-172	1.462950428	0.4	0.072	6.78E-168	Nkcell
C1orf211	4.86E-167	1.625866446	0.412	0.085	1.04E-162	Nkcell
ADGRG11	2.99E-166	1.345910054	0.263	0.033	6.37E-162	Nkcell
TLE4	5.35E-163	1.745675679	0.618	0.2	1.14E-158	Nkcell
RUNX31	1.03E-159	1.443209671	0.674	0.214	2.21E-155	Nkcell
CMC1	1.15E-156	1.785742678	0.441	0.1	2.46E-152	Nkcell
SRGN2	9.18E-154	1.26340053	0.967	0.603	1.96E-149	Nkcell
CYBA1	4.47E-149	1.418167791	0.874	0.503	9.55E-145	Nkcell
HLA-E2	1.58E-144	1.202505077	0.954	0.652	3.38E-140	Nkcell
ZEB21	4.04E-144	1.536439375	0.62	0.211	8.62E-140	Nkcell
B2M1	4.63E-141	0.731243183	1	0.983	9.88E-137	Nkcell
DDIT4	2.52E-136	1.514329007	0.752	0.337	5.37E-132	Nkcell
HCST1	1.12E-127	1.445277685	0.64	0.234	2.39E-123	Nkcell
AUTS2	3.57E-127	1.310221526	0.34	0.069	7.62E-123	Nkcell
RNF125	8.96E-126	1.378768417	0.491	0.139	1.91E-121	Nkcell
GZMM	8.16E-124	1.224058338	0.327	0.065	1.74E-119	Nkcell
ITGB21	2.49E-123	1.399857623	0.412	0.106	5.32E-119	Nkcell
PPP2R5C1	2.54E-123	1.416382828	0.681	0.282	5.43E-119	Nkcell
TNFRSF1B2	3.22E-123	1.359788234	0.557	0.187	6.87E-119	Nkcell
LINC01871	7.15E-123	1.284117704	0.342	0.07	1.53E-118	Nkcell
PABPC11	1.55E-119	1.033862823	0.957	0.734	3.31E-115	Nkcell
GPR65	2.40E-115	1.281765185	0.39	0.096	5.13E-111	Nkcell
GNG2	1.94E-113	1.274470693	0.473	0.139	4.14E-109	Nkcell
TRBC11	2.36E-113	0.965419785	0.575	0.178	5.05E-109	Nkcell
HLA-A1	5.70E-104	0.928663124	0.96	0.789	1.22E-99	Nkcell
SLA1	1.11E-98	1.121994346	0.607	0.23	2.36E-94	Nkcell
MALAT11	1.31E-98	0.504275509	1	0.993	2.80E-94	Nkcell
METRNL1	2.32E-98	1.288465226	0.4	0.119	4.96E-94	Nkcell
FMNL1	1.00E-97	1.364681205	0.484	0.17	2.14E-93	Nkcell
HAVCR2	5.83E-95	1.078128908	0.25	0.049	1.24E-90	Nkcell
MYO1F	8.40E-95	1.129721963	0.271	0.058	1.79E-90	Nkcell
AKAP131	7.30E-94	1.113453944	0.774	0.435	1.56E-89	Nkcell
CD71	4.36E-93	1.083687364	0.597	0.231	9.31E-89	Nkcell
TUBA4A	7.88E-93	1.247790402	0.461	0.154	1.68E-88	Nkcell
LRMP	5.82E-92	1.048222396	0.264	0.056	1.24E-87	Nkcell
IQGAP2	1.00E-90	1.3021116	0.45	0.156	2.14E-86	Nkcell

PTPRC1	2.15E-88	1.016937079	0.79	0.409	4.58E-84	Nkcell
ABHD17A	4.78E-88	1.219176211	0.339	0.096	1.02E-83	Nkcell
SPN	3.57E-87	1.07061728	0.26	0.057	7.62E-83	Nkcell
FOSL21	6.65E-87	1.282159396	0.537	0.229	1.42E-82	Nkcell
TGFB1	8.20E-87	1.213911188	0.46	0.167	1.75E-82	Nkcell
CD531	3.76E-85	1.180922148	0.516	0.202	8.02E-81	Nkcell
CALM11	8.29E-82	0.997399448	0.86	0.608	1.77E-77	Nkcell
PTPN7	1.96E-81	1.163644812	0.393	0.126	4.18E-77	Nkcell
RNF166	4.51E-81	1.067824138	0.269	0.065	9.63E-77	Nkcell
CHD11	1.79E-80	1.284084694	0.539	0.24	3.83E-76	Nkcell
FAM177A1	8.00E-80	1.157064481	0.664	0.352	1.71E-75	Nkcell
LITAF1	2.03E-78	1.140139013	0.6	0.289	4.34E-74	Nkcell
SYNE1	5.11E-78	1.147566618	0.298	0.081	1.09E-73	Nkcell
ADGRE5	3.37E-75	1.088507623	0.489	0.198	7.19E-71	Nkcell
RAB5IF	3.96E-75	1.341047559	0.484	0.214	8.44E-71	Nkcell
CHMP1B1	1.66E-73	1.295833505	0.484	0.21	3.53E-69	Nkcell
PTPRE2	1.46E-72	1.198036695	0.349	0.116	3.12E-68	Nkcell
CNOT2	1.13E-70	1.256960588	0.38	0.139	2.42E-66	Nkcell
BTG11	1.41E-70	0.65012976	0.983	0.791	3.01E-66	Nkcell
DUSP21	5.81E-70	0.923310371	0.564	0.244	1.24E-65	Nkcell
ATP1B32	7.51E-70	1.142002241	0.621	0.33	1.60E-65	Nkcell
RALGAPA1	1.67E-69	1.049380027	0.43	0.161	3.57E-65	Nkcell
ISG202	7.78E-69	0.954560029	0.64	0.327	1.66E-64	Nkcell
SYAP11	2.01E-68	1.133080577	0.62	0.34	4.29E-64	Nkcell
ARPC22	9.77E-68	0.928146325	0.802	0.557	2.09E-63	Nkcell
PYHIN1	5.62E-67	0.955411699	0.263	0.069	1.20E-62	Nkcell
CNOT6L1	5.65E-67	0.83590977	0.621	0.29	1.21E-62	Nkcell
POLR2A	1.98E-66	1.121501587	0.474	0.21	4.22E-62	Nkcell
SRSF21	4.65E-66	1.076350724	0.674	0.416	9.92E-62	Nkcell
DDX51	1.42E-65	0.688893263	0.937	0.741	3.04E-61	Nkcell
GNPTAB	5.33E-65	1.205999431	0.34	0.119	1.14E-60	Nkcell
ARF61	2.71E-64	1.017981633	0.605	0.329	5.79E-60	Nkcell
RBM391	3.01E-64	0.892913213	0.795	0.548	6.43E-60	Nkcell
RAB29	6.65E-64	1.097731641	0.255	0.072	1.42E-59	Nkcell
NR4A21	7.59E-64	1.041938879	0.522	0.243	1.62E-59	Nkcell
VPS37B	2.91E-63	1.007663887	0.41	0.159	6.22E-59	Nkcell
DSTN3	7.10E-63	0.82887376	0.681	0.377	1.51E-58	Nkcell
C1orf56	5.86E-62	0.973669811	0.35	0.123	1.25E-57	Nkcell
LDHA2	9.23E-62	1.02155883	0.689	0.439	1.97E-57	Nkcell
SRSF51	2.07E-60	0.930724642	0.688	0.428	4.41E-56	Nkcell
PDE4D	1.47E-59	0.94222836	0.415	0.165	3.13E-55	Nkcell
CXCR41	2.10E-59	0.806433523	0.798	0.48	4.47E-55	Nkcell
RASSF5	2.25E-59	0.954871094	0.253	0.073	4.81E-55	Nkcell

S100A42	2.44E-59	0.791344687	0.793	0.49	5.21E-55	Nkcell
RPS261	4.03E-59	0.632030953	0.936	0.741	8.60E-55	Nkcell
FYN1	4.79E-59	0.856949715	0.574	0.275	1.02E-54	Nkcell
CCND2	9.63E-59	1.074562346	0.387	0.156	2.06E-54	Nkcell
ID2	1.12E-57	0.982473189	0.603	0.321	2.39E-53	Nkcell
XIST2	4.64E-57	0.994838344	0.555	0.288	9.90E-53	Nkcell
LRRFIP11	4.71E-57	0.972158641	0.698	0.446	1.01E-52	Nkcell
SUN2	1.07E-55	0.941872526	0.256	0.079	2.27E-51	Nkcell
PDE7A	2.13E-55	0.987392649	0.312	0.107	4.54E-51	Nkcell
MBP	2.27E-55	1.081275939	0.379	0.155	4.84E-51	Nkcell
MYL12A1	1.06E-54	0.971698652	0.683	0.455	2.27E-50	Nkcell
CEMIP21	1.29E-54	0.983189963	0.438	0.192	2.76E-50	Nkcell
DNAJB6	1.35E-54	0.883633206	0.617	0.365	2.88E-50	Nkcell
RPS10	7.66E-54	0.596233736	0.919	0.739	1.63E-49	Nkcell
RAP1B	5.98E-53	0.975953853	0.522	0.286	1.28E-48	Nkcell
FLNA1	1.96E-52	1.037440234	0.403	0.182	4.19E-48	Nkcell
CDC42SE1	2.41E-52	0.967752016	0.398	0.171	5.15E-48	Nkcell
PIP4K2A	1.18E-51	0.815597533	0.311	0.11	2.53E-47	Nkcell
PTMA1	1.19E-50	0.442835862	0.995	0.965	2.54E-46	Nkcell
ARHGAP9	5.91E-50	0.805689037	0.256	0.081	1.26E-45	Nkcell
SELENOK	7.83E-50	0.906593288	0.691	0.476	1.67E-45	Nkcell
PRKX	1.82E-49	0.865225004	0.291	0.102	3.88E-45	Nkcell
PRKCH	6.30E-49	0.895023673	0.314	0.118	1.34E-44	Nkcell
ARHGDIB1	9.87E-49	0.824053406	0.6	0.334	2.11E-44	Nkcell
DHRS7	1.27E-48	1.102606601	0.332	0.14	2.72E-44	Nkcell
STK41	6.01E-48	0.70792055	0.623	0.338	1.28E-43	Nkcell
IRF11	5.43E-47	0.842547579	0.572	0.327	1.16E-42	Nkcell
RBM38	1.95E-46	0.829902988	0.298	0.109	4.17E-42	Nkcell
AAK11	2.46E-46	0.803484577	0.555	0.307	5.24E-42	Nkcell
TERF1	3.26E-46	0.997904585	0.263	0.093	6.97E-42	Nkcell
RBMX1	1.22E-45	0.93054705	0.617	0.396	2.60E-41	Nkcell
TNFAIP32	2.56E-45	0.72557578	0.704	0.431	5.46E-41	Nkcell
SIRT2	2.38E-44	0.906828847	0.258	0.092	5.09E-40	Nkcell
RNF168	2.26E-43	0.964750466	0.268	0.1	4.82E-39	Nkcell
LCP11	2.04E-42	0.823628116	0.385	0.175	4.36E-38	Nkcell
STAT4	4.62E-42	0.792577703	0.294	0.112	9.86E-38	Nkcell
KMT2E1	1.58E-41	0.792792135	0.605	0.381	3.38E-37	Nkcell
TCF25	1.79E-41	0.781456923	0.608	0.407	3.82E-37	Nkcell
SYNE21	2.61E-41	0.856450095	0.46	0.233	5.56E-37	Nkcell
NR4A31	2.77E-41	0.919112496	0.279	0.108	5.91E-37	Nkcell
REL2	9.89E-41	0.823262862	0.668	0.422	2.11E-36	Nkcell
PRPF38B1	1.11E-40	0.819657582	0.574	0.354	2.38E-36	Nkcell
UBB1	1.33E-39	0.432275846	0.883	0.677	2.84E-35	Nkcell

SLC7A5	3.28E-39	0.838677643	0.283	0.112	7.00E-35	Nkcell
IL2RG	3.51E-39	0.824965029	0.342	0.152	7.48E-35	Nkcell
JMJD6	1.52E-38	0.81016924	0.298	0.123	3.25E-34	Nkcell
CCND3	1.74E-38	0.857142047	0.261	0.102	3.72E-34	Nkcell
LSP11	1.86E-38	0.760176125	0.426	0.217	3.97E-34	Nkcell
EFHD22	6.55E-38	0.983320563	0.355	0.175	1.40E-33	Nkcell
SLC38A1	1.83E-37	0.825745357	0.355	0.165	3.90E-33	Nkcell
IDI1	4.78E-37	0.925581714	0.35	0.166	1.02E-32	Nkcell
GABARAPL	7.92E-37	0.810012778	0.36	0.172	1.69E-32	Nkcell
C9orf78	2.25E-36	0.856564533	0.374	0.187	4.80E-32	Nkcell
RPL171	9.95E-36	0.572447774	0.74	0.517	2.12E-31	Nkcell
CCNH	3.32E-35	0.851410801	0.299	0.131	7.09E-31	Nkcell
RNF2131	4.56E-35	0.731357185	0.565	0.363	9.72E-31	Nkcell
CORO1A1	4.82E-35	0.678703283	0.426	0.215	1.03E-30	Nkcell
PTGER4	1.35E-34	0.741282286	0.26	0.103	2.88E-30	Nkcell
HLA-C1	3.98E-34	0.435820483	0.884	0.735	8.50E-30	Nkcell
IKZF11	4.76E-34	0.62784242	0.375	0.177	1.02E-29	Nkcell
CD992	6.97E-34	0.73349483	0.519	0.319	1.49E-29	Nkcell
OSTF1	1.04E-33	0.855851722	0.281	0.125	2.22E-29	Nkcell
PABPC41	1.19E-33	0.852827276	0.498	0.31	2.55E-29	Nkcell
RAC2	5.40E-32	0.700888785	0.357	0.175	1.15E-27	Nkcell
CFL12	1.26E-31	0.534210798	0.805	0.629	2.69E-27	Nkcell
MAGOH	1.38E-31	0.797338841	0.279	0.128	2.95E-27	Nkcell
FAM133B	2.47E-31	0.694366396	0.559	0.372	5.28E-27	Nkcell
DNTTIP2	4.00E-31	0.768828844	0.293	0.136	8.53E-27	Nkcell
SRSF71	4.02E-31	0.476639584	0.641	0.421	8.59E-27	Nkcell
DHX361	3.09E-30	0.703682135	0.471	0.285	6.59E-26	Nkcell
SON1	1.11E-29	0.592306356	0.658	0.489	2.36E-25	Nkcell
BRD7	1.26E-29	0.871803793	0.289	0.14	2.69E-25	Nkcell
RPL36AL1	3.32E-29	0.542519757	0.792	0.648	7.08E-25	Nkcell
RBM8A	5.94E-29	0.74412444	0.491	0.322	1.27E-24	Nkcell
RSRP11	9.29E-29	0.743552711	0.456	0.283	1.98E-24	Nkcell
DDX241	1.55E-28	0.522373016	0.661	0.477	3.31E-24	Nkcell
EIF11	1.61E-28	0.353426214	0.977	0.882	3.43E-24	Nkcell
LIMD2	2.19E-28	0.739877135	0.269	0.121	4.67E-24	Nkcell
CDK13	4.68E-28	0.77070534	0.276	0.13	9.99E-24	Nkcell
HNRNPA1	1.55E-27	0.504893366	0.793	0.619	3.30E-23	Nkcell
AREG1	1.96E-27	0.781549447	0.374	0.204	4.19E-23	Nkcell
SUB11	7.91E-27	0.630367421	0.625	0.47	1.69E-22	Nkcell
ZNF3311	1.23E-26	0.521880199	0.431	0.239	2.63E-22	Nkcell
NFE2L21	2.65E-26	0.774468366	0.445	0.285	5.66E-22	Nkcell
IFITM22	9.21E-26	0.539218917	0.577	0.388	1.97E-21	Nkcell
OTULIN	3.39E-25	0.708841416	0.261	0.123	7.24E-21	Nkcell

BPTF	4.08E-25	0.757046464	0.357	0.206	8.71E-21	Nkcell
HNRNPA2	1.07E-24	0.578467679	0.588	0.432	2.28E-20	Nkcell
PIK3R11	1.62E-24	0.670478595	0.375	0.212	3.45E-20	Nkcell
JAK11	2.60E-24	0.670617737	0.483	0.323	5.55E-20	Nkcell
ETS11	2.80E-24	0.499916053	0.476	0.284	5.97E-20	Nkcell
PNRC11	3.01E-24	0.5316448	0.676	0.508	6.43E-20	Nkcell
H3F3B1	4.29E-24	0.325953743	0.947	0.833	9.16E-20	Nkcell
DDX3X	6.31E-24	0.667453675	0.507	0.352	1.35E-19	Nkcell
UTRN1	6.45E-24	0.686113257	0.266	0.132	1.38E-19	Nkcell
AC245297.	7.02E-24	0.725576667	0.317	0.168	1.50E-19	Nkcell
PHF20	1.54E-23	0.773656691	0.316	0.174	3.28E-19	Nkcell
TSC22D31	1.55E-23	0.469788565	0.782	0.599	3.30E-19	Nkcell
CD69	1.68E-23	0.4835276	0.296	0.143	3.59E-19	Nkcell
BZW11	6.77E-23	0.696036817	0.418	0.272	1.44E-18	Nkcell
ARID4B1	7.70E-23	0.572889596	0.502	0.335	1.64E-18	Nkcell
SRRM11	1.65E-22	0.587019575	0.544	0.396	3.51E-18	Nkcell
YAF2	1.98E-22	0.786369459	0.271	0.143	4.22E-18	Nkcell
GLIPR1	1.99E-22	0.690642744	0.296	0.157	4.24E-18	Nkcell
RORA1	2.36E-22	0.548992718	0.38	0.219	5.04E-18	Nkcell
CEBPB2	3.22E-22	0.583855666	0.486	0.322	6.88E-18	Nkcell
MCL11	3.50E-22	0.643691748	0.607	0.472	7.46E-18	Nkcell
HNRNPA3	5.63E-22	0.692991043	0.43	0.29	1.20E-17	Nkcell
MT-ND3	1.12E-21	0.339350804	0.911	0.774	2.40E-17	Nkcell
MSN1	2.23E-21	0.648200042	0.369	0.226	4.77E-17	Nkcell
SCAF111	3.64E-21	0.657032386	0.479	0.339	7.78E-17	Nkcell
C12orf751	6.14E-21	0.587174883	0.258	0.13	1.31E-16	Nkcell
AC026979.	7.92E-21	0.613331231	0.266	0.134	1.69E-16	Nkcell
TES1	1.36E-20	0.770702845	0.327	0.196	2.90E-16	Nkcell
YPEL51	3.16E-20	0.486740863	0.451	0.293	6.75E-16	Nkcell
EVL1	5.33E-20	0.570230858	0.336	0.19	1.14E-15	Nkcell
HNRNPL	7.48E-20	0.764467865	0.269	0.149	1.60E-15	Nkcell
NCL	1.26E-19	0.464560058	0.669	0.535	2.70E-15	Nkcell
MECP2	1.37E-19	0.592787286	0.326	0.19	2.93E-15	Nkcell
ZFP362	1.60E-19	0.40750637	0.631	0.456	3.41E-15	Nkcell
FNBP11	2.36E-19	0.525777699	0.438	0.283	5.04E-15	Nkcell
MAFF2	4.55E-19	0.7360963	0.281	0.16	9.72E-15	Nkcell
CD47	6.13E-19	0.628635012	0.269	0.15	1.31E-14	Nkcell
EIF1AX	6.63E-19	0.672103641	0.44	0.314	1.42E-14	Nkcell
CELF21	7.62E-19	0.618456076	0.359	0.218	1.63E-14	Nkcell
PHF20L1	9.32E-19	0.718710369	0.256	0.139	1.99E-14	Nkcell
EMP32	1.08E-18	0.5909542	0.398	0.262	2.31E-14	Nkcell
FUS1	1.99E-18	0.478328606	0.615	0.477	4.24E-14	Nkcell
WIPF1	3.28E-18	0.5314262	0.319	0.187	7.00E-14	Nkcell

H2AFZ1	4.99E-18	0.554113921	0.545	0.413	1.07E-13	Nkcell
SFPQ1	5.11E-18	0.468984251	0.54	0.402	1.09E-13	Nkcell
HNRNPDL	5.25E-18	0.552051808	0.526	0.404	1.12E-13	Nkcell
SARAF1	1.55E-17	0.327068363	0.636	0.476	3.32E-13	Nkcell
CCDC107	1.74E-17	0.650078562	0.286	0.169	3.71E-13	Nkcell
ELF11	1.96E-17	0.521905517	0.484	0.344	4.17E-13	Nkcell
KANSL1	2.13E-17	0.633030399	0.286	0.165	4.54E-13	Nkcell
KLF31	2.22E-17	0.600625927	0.288	0.169	4.74E-13	Nkcell
VAMP2	3.72E-17	0.581990098	0.364	0.238	7.94E-13	Nkcell
DYNC1H11	4.78E-17	0.620312804	0.369	0.244	1.02E-12	Nkcell
PRMT21	6.99E-17	0.624509029	0.369	0.244	1.49E-12	Nkcell
SLTM1	1.43E-16	0.647692199	0.375	0.254	3.05E-12	Nkcell
IER22	4.76E-16	0.407086883	0.469	0.331	1.02E-11	Nkcell
SP100	7.82E-16	0.582187974	0.345	0.226	1.67E-11	Nkcell
CD372	8.28E-16	0.338691464	0.392	0.246	1.77E-11	Nkcell
BRD2	9.25E-16	0.5274843	0.441	0.324	1.97E-11	Nkcell
MIDN1	1.12E-15	0.577121198	0.367	0.248	2.39E-11	Nkcell
HLA-F	1.76E-15	0.538806927	0.274	0.163	3.75E-11	Nkcell
CRYBG11	2.32E-15	0.499362167	0.274	0.155	4.95E-11	Nkcell
PGK11	7.68E-15	0.631632725	0.379	0.27	1.64E-10	Nkcell
HMGB1	8.97E-15	0.298311531	0.744	0.617	1.92E-10	Nkcell
DDX27	1.25E-14	0.557636404	0.304	0.19	2.66E-10	Nkcell
ARID5A	1.51E-14	0.526016475	0.253	0.146	3.21E-10	Nkcell
SDCBP3	1.57E-14	0.608485634	0.425	0.318	3.35E-10	Nkcell
NEAT13	2.12E-14	0.255994144	0.952	0.842	4.52E-10	Nkcell
HSPA52	2.60E-14	0.616654435	0.331	0.224	5.55E-10	Nkcell
ARL6IP1	4.50E-14	0.516393551	0.387	0.276	9.60E-10	Nkcell
HNRNPM1	5.02E-14	0.522214268	0.393	0.286	1.07E-09	Nkcell
SRSF3	2.28E-13	0.496554397	0.473	0.371	4.86E-09	Nkcell
ANXA12	2.42E-13	0.314915921	0.468	0.331	5.16E-09	Nkcell
PCSK7	2.56E-13	0.505751959	0.294	0.186	5.46E-09	Nkcell
TMA7	2.73E-13	0.308842645	0.734	0.633	5.83E-09	Nkcell
PDIA32	4.85E-13	0.686145882	0.307	0.215	1.04E-08	Nkcell
FAM32A	6.23E-13	0.634622618	0.25	0.157	1.33E-08	Nkcell
SRRM2	1.44E-12	0.47967039	0.479	0.388	3.08E-08	Nkcell
SAFB2	1.54E-12	0.55176918	0.264	0.168	3.28E-08	Nkcell
SKIL	1.57E-12	0.532696076	0.289	0.189	3.36E-08	Nkcell
RRBP13	1.61E-12	0.653882525	0.39	0.293	3.44E-08	Nkcell
HNRNPA0	1.71E-12	0.602208739	0.354	0.258	3.65E-08	Nkcell
CD481	3.96E-12	0.312860125	0.301	0.186	8.45E-08	Nkcell
GMFG1	4.22E-12	0.389098555	0.357	0.241	9.00E-08	Nkcell
Sep-71	4.39E-12	0.406357943	0.425	0.315	9.36E-08	Nkcell
TLN11	4.64E-12	0.508244006	0.327	0.229	9.90E-08	Nkcell

HNRNPUL	5.40E-12	0.532480944	0.311	0.213	1.15E-07	Nkcell
DDX6	6.64E-12	0.494728145	0.279	0.181	1.42E-07	Nkcell
IDS1	7.18E-12	0.366439619	0.4	0.282	1.53E-07	Nkcell
POLR2L3	8.91E-12	0.387505356	0.517	0.419	1.90E-07	Nkcell
TPM32	1.13E-11	0.387596108	0.521	0.416	2.42E-07	Nkcell
PSME1	1.19E-11	0.386071676	0.539	0.445	2.54E-07	Nkcell
PRDM1	1.28E-11	0.459609195	0.306	0.2	2.72E-07	Nkcell
AC058791.	1.38E-11	0.314435486	0.392	0.269	2.95E-07	Nkcell
SAP18	1.88E-11	0.430337414	0.461	0.371	4.01E-07	Nkcell
FAM49B1	2.30E-11	0.508294944	0.286	0.19	4.92E-07	Nkcell
ANKRD111	2.57E-11	0.462622002	0.418	0.322	5.49E-07	Nkcell
CYCS1	2.79E-11	0.365850926	0.436	0.336	5.95E-07	Nkcell
SKP11	3.20E-11	0.350276622	0.539	0.438	6.84E-07	Nkcell
TRA2A	4.90E-11	0.424350523	0.385	0.286	1.05E-06	Nkcell
WAC	5.27E-11	0.49990065	0.327	0.234	1.13E-06	Nkcell
ACTR21	5.34E-11	0.560775629	0.324	0.238	1.14E-06	Nkcell
ENSA	7.36E-11	0.59607978	0.299	0.214	1.57E-06	Nkcell
PFN12	1.00E-10	0.298870921	0.711	0.598	2.14E-06	Nkcell
DBI2	1.08E-10	0.421726346	0.441	0.351	2.31E-06	Nkcell
RGS21	1.30E-10	0.269570738	0.36	0.249	2.77E-06	Nkcell
B4GALT11	2.19E-10	0.482543234	0.25	0.163	4.67E-06	Nkcell
PNN	2.21E-10	0.527124428	0.306	0.216	4.72E-06	Nkcell
MYL12B2	2.68E-10	0.33419665	0.55	0.453	5.72E-06	Nkcell
CEBPZ	2.81E-10	0.421035379	0.273	0.183	6.01E-06	Nkcell
AES	3.57E-10	0.579782502	0.273	0.191	7.62E-06	Nkcell
ABCF1	3.60E-10	0.482898307	0.321	0.234	7.68E-06	Nkcell
CALR3	7.91E-10	0.422300344	0.448	0.364	1.69E-05	Nkcell
PRPF4B	8.41E-10	0.450275202	0.342	0.251	1.80E-05	Nkcell
TMF1	1.02E-09	0.54046321	0.268	0.184	2.17E-05	Nkcell
EIF3G	1.03E-09	0.496886675	0.352	0.274	2.21E-05	Nkcell
PRRC2C1	1.04E-09	0.342998743	0.545	0.459	2.22E-05	Nkcell
SELENOT	1.60E-09	0.534968468	0.261	0.183	3.42E-05	Nkcell
DEK	2.81E-09	0.392556636	0.425	0.341	5.99E-05	Nkcell
TMEM50A	4.34E-09	0.479166876	0.326	0.25	9.25E-05	Nkcell
CALM22	4.80E-09	0.295099012	0.595	0.504	0.000102	Nkcell
SH3KBP1	5.17E-09	0.47311602	0.256	0.177	0.00011	Nkcell
CLEC2B1	7.14E-09	0.344000188	0.264	0.178	0.000152	Nkcell
CD1642	8.57E-09	0.524283712	0.293	0.22	0.000183	Nkcell
NOP56	1.05E-08	0.470796913	0.289	0.211	0.000225	Nkcell
TSPYL21	1.12E-08	0.27117811	0.342	0.245	0.000239	Nkcell
CDC422	1.23E-08	0.320586441	0.486	0.408	0.000262	Nkcell
USP15	2.36E-08	0.401285371	0.269	0.188	0.000503	Nkcell
CYTIP1	5.43E-08	0.269892063	0.316	0.221	0.00116	Nkcell

SRSF111	5.66E-08	0.400497722	0.44	0.372	0.001208	Nkcell
EIF5	6.81E-08	0.39222156	0.426	0.359	0.001454	Nkcell
FNBP4	7.66E-08	0.447910395	0.25	0.177	0.001634	Nkcell
PAIP2	9.98E-08	0.383389427	0.312	0.238	0.002129	Nkcell
TERF2IP	1.02E-07	0.412207429	0.294	0.221	0.002179	Nkcell
RBM251	1.16E-07	0.360098423	0.45	0.383	0.002482	Nkcell
YY1	1.23E-07	0.447369957	0.281	0.214	0.002635	Nkcell
CDC42SE2	2.67E-07	0.263398603	0.312	0.228	0.005703	Nkcell
CD3G1	2.81E-07	0.406822905	0.261	0.182	0.005995	Nkcell
YTHDC1	3.12E-07	0.350540282	0.317	0.246	0.006664	Nkcell
AMD11	3.20E-07	0.341260466	0.352	0.277	0.006824	Nkcell
TRIR	3.60E-07	0.335959067	0.428	0.365	0.007686	Nkcell
BAZ1B	3.72E-07	0.429182581	0.26	0.192	0.007942	Nkcell
NDUFB22	4.69E-07	0.372970461	0.392	0.333	0.010017	Nkcell
ITGB12	6.30E-07	0.326237574	0.438	0.361	0.013439	Nkcell
HNRNPF	7.96E-07	0.462049415	0.253	0.191	0.016983	Nkcell
RARRES31	9.12E-07	0.257704524	0.312	0.235	0.019456	Nkcell
DDX18	9.34E-07	0.424696838	0.314	0.25	0.019945	Nkcell
TAF7	2.37E-06	0.332218961	0.256	0.19	0.050682	Nkcell
STAT31	2.66E-06	0.264584033	0.393	0.321	0.056753	Nkcell
NDUFS52	2.89E-06	0.305709874	0.511	0.459	0.06169	Nkcell
UBE2D3	3.11E-06	0.316209728	0.468	0.416	0.0664	Nkcell
PNISR1	3.72E-06	0.287096247	0.4	0.334	0.079347	Nkcell
ROCK1	6.43E-06	0.424200159	0.288	0.231	0.137208	Nkcell
DDX461	1.22E-05	0.29127377	0.326	0.266	0.259648	Nkcell
LUC7L3	1.44E-05	0.364378153	0.293	0.237	0.30661	Nkcell
IK	1.71E-05	0.373010898	0.25	0.193	0.365677	Nkcell
SNRPB1	1.75E-05	0.325723312	0.304	0.248	0.372779	Nkcell
PDCD41	2.62E-05	0.256534113	0.281	0.215	0.559696	Nkcell
GNAI22	2.90E-05	0.334224357	0.301	0.246	0.61995	Nkcell
ZC3H15	2.92E-05	0.342859913	0.302	0.253	0.623295	Nkcell
MPHOSPH	4.17E-05	0.359105521	0.302	0.25	0.88999	Nkcell
CAP11	4.24E-05	0.322827805	0.294	0.244	0.905908	Nkcell
CIB12	4.36E-05	0.271925871	0.345	0.29	0.930327	Nkcell
NASP	4.71E-05	0.38946467	0.274	0.222	1	Nkcell
EPC11	5.19E-05	0.301477498	0.311	0.255	1	Nkcell
AP2B11	6.85E-05	0.38346813	0.271	0.224	1	Nkcell
TNRC6B1	7.21E-05	0.404376329	0.299	0.251	1	Nkcell
EIF3A	9.07E-05	0.337273607	0.35	0.309	1	Nkcell
SYF2	9.67E-05	0.256876117	0.369	0.323	1	Nkcell
TAX1BP12	0.000116	0.376115076	0.291	0.247	1	Nkcell
IFI161	0.000132	0.313279534	0.336	0.284	1	Nkcell
HNRNPK1	0.000151	0.317742508	0.383	0.351	1	Nkcell

ARL6IP5	0.000186	0.306524018	0.301	0.254	1	Nkcell
ATRX	0.000196	0.30017971	0.298	0.25	1	Nkcell
MBNL11	0.000255	0.268447383	0.35	0.307	1	Nkcell
SH3GLB11	0.000293	0.313159313	0.271	0.229	1	Nkcell
AKAP9	0.000535	0.263292921	0.367	0.33	1	Nkcell
ZC3HAV11	0.000686	0.267545738	0.279	0.231	1	Nkcell
ATP2B11	0.000697	0.277993023	0.261	0.218	1	Nkcell
SLC38A22	0.000733	0.278979767	0.331	0.291	1	Nkcell
SF3B2	0.000736	0.336619793	0.269	0.232	1	Nkcell
TACC12	0.001652	0.293560121	0.256	0.218	1	Nkcell
SSR2	0.002268	0.269405753	0.369	0.35	1	Nkcell
RAB2A2	0.003244	0.345086049	0.269	0.241	1	Nkcell
TPR	0.003971	0.254255955	0.263	0.229	1	Nkcell
SPCS21	0.004364	0.344017117	0.284	0.26	1	Nkcell
CAPZA1	0.00528	0.328436938	0.266	0.239	1	Nkcell
TTC31	0.006082	0.351180025	0.251	0.226	1	Nkcell
HNRNPAB	0.007553	0.274345054	0.26	0.234	1	Nkcell
NCOR11	0.00792	0.255659075	0.271	0.243	1	Nkcell
GOLGA4	0.00863	0.299421524	0.25	0.223	1	Nkcell
TPSB2	0	7.287850104	0.989	0.012	0	Granulocyte
TPSAB1	0	5.823660877	0.848	0.009	0	Granulocyte
CPA3	0	5.141868295	0.91	0.002	0	Granulocyte
LTC4S	0	3.378218577	0.719	0.024	0	Granulocyte
MS4A2	0	3.252173482	0.646	0.001	0	Granulocyte
GATA2	0	3.00077691	0.596	0.01	0	Granulocyte
HPGDS	0	2.945055389	0.59	0.001	0	Granulocyte
KIT	0	2.943076588	0.652	0.003	0	Granulocyte
IL1RL1	0	2.785932991	0.635	0.012	0	Granulocyte
SLC18A2	0	2.44576128	0.539	0.001	0	Granulocyte
RGS13	0	2.206168985	0.433	0.008	0	Granulocyte
HDC	0	2.114423547	0.41	0.001	0	Granulocyte
RHEX	0	1.697217361	0.315	0.004	0	Granulocyte
AL157895.	0	1.695791982	0.32	0.001	0	Granulocyte
MAOB	0	1.405744228	0.298	0.006	0	Granulocyte
TMEM233	5.70E-277	1.670046716	0.27	0.006	1.22E-272	Granulocyte
VWA5A	2.60E-252	1.673185385	0.354	0.013	5.55E-248	Granulocyte
CLU2	1.09E-224	2.801977664	0.809	0.096	2.33E-220	Granulocyte
CPM	3.80E-193	2.419835512	0.539	0.047	8.10E-189	Granulocyte
BTK	5.34E-167	1.269901522	0.275	0.013	1.14E-162	Granulocyte
SLC26A2	3.08E-149	1.913211234	0.36	0.026	6.57E-145	Granulocyte
AREG2	5.68E-118	3.092263504	0.831	0.204	1.21E-113	Granulocyte
ALOX5	6.06E-115	1.782162192	0.365	0.035	1.29E-110	Granulocyte
FCER1G1	2.69E-106	1.659154945	0.652	0.111	5.75E-102	Granulocyte

SMYD3	5.11E-92	1.361028771	0.309	0.031	1.09E-87	Granulocyte
ALOX5AP1	4.12E-81	2.018828008	0.685	0.168	8.79E-77	Granulocyte
HPGD	2.11E-80	2.019201597	0.483	0.084	4.50E-76	Granulocyte
ACSL4	2.96E-69	1.603074432	0.388	0.064	6.32E-65	Granulocyte
LEO1	8.93E-69	1.329996048	0.292	0.036	1.91E-64	Granulocyte
LMNA2	4.85E-66	2.0403387	0.854	0.398	1.04E-61	Granulocyte
CD691	9.91E-66	2.109008401	0.584	0.144	2.12E-61	Granulocyte
BMP2K	2.50E-65	1.109234475	0.253	0.029	5.34E-61	Granulocyte
DUSP61	2.64E-65	1.883892151	0.433	0.083	5.64E-61	Granulocyte
CD95	3.47E-62	1.990100052	0.635	0.197	7.41E-58	Granulocyte
CPEB42	1.56E-61	2.086639392	0.528	0.132	3.33E-57	Granulocyte
ARHGEF6	6.95E-59	0.927833393	0.253	0.031	1.48E-54	Granulocyte
FOSB2	2.67E-56	1.753126247	0.882	0.45	5.69E-52	Granulocyte
SRGN3	4.59E-56	1.61983915	0.961	0.616	9.80E-52	Granulocyte
NSMCE1	2.38E-55	1.717361338	0.404	0.084	5.09E-51	Granulocyte
RGS22	4.42E-55	1.934355347	0.697	0.248	9.43E-51	Granulocyte
GALC	7.88E-54	1.251802554	0.27	0.039	1.68E-49	Granulocyte
B4GALT5	4.05E-53	1.509357657	0.36	0.069	8.64E-49	Granulocyte
FTH11	1.14E-52	1.053258333	0.983	0.914	2.43E-48	Granulocyte
TYROBP2	9.71E-52	0.903456241	0.64	0.172	2.07E-47	Granulocyte
LAPTM4A2	1.55E-49	1.90965758	0.669	0.257	3.31E-45	Granulocyte
CTNNBL1	6.91E-49	1.351499804	0.376	0.079	1.47E-44	Granulocyte
GLUL2	2.76E-43	1.605643796	0.601	0.212	5.90E-39	Granulocyte
GPR651	2.95E-41	1.439755096	0.421	0.106	6.31E-37	Granulocyte
PBX11	3.25E-41	1.165709505	0.264	0.047	6.94E-37	Granulocyte
C1orf1621	6.81E-41	0.854166918	0.264	0.046	1.45E-36	Granulocyte
CAPG1	2.32E-40	1.551803653	0.455	0.133	4.96E-36	Granulocyte
VIM3	4.38E-40	1.380882642	0.888	0.598	9.34E-36	Granulocyte
CSF1	2.34E-39	1.395675167	0.253	0.045	4.99E-35	Granulocyte
KDM6B1	4.43E-37	1.508517809	0.584	0.226	9.45E-33	Granulocyte
PLIN21	5.06E-36	1.484885497	0.494	0.163	1.08E-31	Granulocyte
CD634	1.16E-35	1.499176254	0.753	0.402	2.47E-31	Granulocyte
RPL23	1.24E-35	0.856013641	0.972	0.858	2.64E-31	Granulocyte
VEGFA1	1.96E-35	1.394919513	0.371	0.099	4.17E-31	Granulocyte
ARHGAP18	2.18E-35	1.390862028	0.365	0.096	4.66E-31	Granulocyte
ID21	1.61E-34	1.606487436	0.697	0.33	3.44E-30	Granulocyte
LAT	1.21E-33	1.114739851	0.337	0.082	2.58E-29	Granulocyte
PAK1	2.51E-33	1.069016527	0.287	0.064	5.35E-29	Granulocyte
CD442	4.02E-32	1.151549647	0.775	0.414	8.58E-28	Granulocyte
PTGS21	4.83E-32	1.114995938	0.253	0.052	1.03E-27	Granulocyte
BACE21	6.46E-32	0.99678567	0.287	0.068	1.38E-27	Granulocyte
SELENOK1	1.44E-31	1.64690002	0.781	0.483	3.07E-27	Granulocyte
FDX1	2.61E-31	1.517886102	0.404	0.13	5.58E-27	Granulocyte

NFKBIA3	4.79E-31	1.470244738	0.781	0.49	1.02E-26	Granulocyte
TWISTNB	2.41E-30	1.438410739	0.36	0.105	5.14E-26	Granulocyte
RPL341	7.75E-30	0.675444472	0.989	0.962	1.65E-25	Granulocyte
H3F3B2	9.14E-30	0.902787661	0.955	0.837	1.95E-25	Granulocyte
SAMSN12	1.60E-29	1.151496914	0.596	0.245	3.41E-25	Granulocyte
MAPK6	2.07E-29	1.411699926	0.365	0.112	4.42E-25	Granulocyte
ELL21	2.12E-29	1.239206475	0.461	0.163	4.53E-25	Granulocyte
DUSP10	8.69E-28	1.05233847	0.281	0.07	1.85E-23	Granulocyte
PTMA2	5.85E-27	0.578803404	1	0.966	1.25E-22	Granulocyte
RGS11	1.90E-25	1.229804398	0.758	0.432	4.06E-21	Granulocyte
SYAP12	3.27E-25	1.338536905	0.657	0.35	6.98E-21	Granulocyte
BCL2A11	4.66E-24	0.997028649	0.258	0.066	9.96E-20	Granulocyte
CD831	6.70E-24	1.029156255	0.365	0.118	1.43E-19	Granulocyte
SDCBP4	9.87E-24	1.234582237	0.607	0.319	2.11E-19	Granulocyte
SKIL1	2.82E-23	1.248880849	0.461	0.19	6.01E-19	Granulocyte
RASGEF1B	8.35E-23	1.141620263	0.253	0.067	1.78E-18	Granulocyte
PAG1	1.76E-22	0.945426793	0.326	0.103	3.76E-18	Granulocyte
FOXP11	2.00E-22	1.090906839	0.624	0.317	4.26E-18	Granulocyte
RPL37A2	2.00E-22	0.630954203	0.978	0.941	4.27E-18	Granulocyte
ANXA13	2.80E-22	1.484345834	0.624	0.334	5.98E-18	Granulocyte
CD82	3.34E-22	0.87462373	0.298	0.09	7.13E-18	Granulocyte
ASAH11	1.10E-21	1.001548924	0.371	0.136	2.36E-17	Granulocyte
RAC21	1.18E-21	0.939379622	0.449	0.18	2.51E-17	Granulocyte
SWAP701	3.69E-21	0.866708355	0.264	0.077	7.89E-17	Granulocyte
GNPTAB1	6.43E-21	0.884575944	0.36	0.127	1.37E-16	Granulocyte
MAPK1	6.64E-21	0.93983413	0.298	0.094	1.42E-16	Granulocyte
AHR1	2.09E-19	0.851667606	0.275	0.087	4.47E-15	Granulocyte
CKLF1	2.14E-19	1.131083617	0.427	0.182	4.58E-15	Granulocyte
REL3	2.34E-19	1.09085228	0.68	0.431	5.00E-15	Granulocyte
PPP1R15A	6.79E-19	1.255961794	0.657	0.399	1.45E-14	Granulocyte
UBB2	7.75E-19	1.006257018	0.893	0.684	1.65E-14	Granulocyte
NFKBIZ2	8.90E-18	1.22669231	0.438	0.209	1.90E-13	Granulocyte
PHF201	9.85E-18	0.918501636	0.416	0.177	2.10E-13	Granulocyte
TSC22D12	1.08E-17	1.236387092	0.388	0.162	2.29E-13	Granulocyte
LMO41	1.81E-17	0.957830492	0.449	0.207	3.87E-13	Granulocyte
SLC44A1	1.07E-16	0.88807792	0.258	0.088	2.28E-12	Granulocyte
CDC42EP3	1.79E-16	0.942629627	0.281	0.098	3.82E-12	Granulocyte
PLAUR2	4.02E-16	0.63754137	0.371	0.151	8.58E-12	Granulocyte
CD373	1.12E-15	0.585779927	0.522	0.249	2.39E-11	Granulocyte
GNAQ2	2.30E-15	0.768918996	0.275	0.102	4.92E-11	Granulocyte
FAU	2.77E-15	0.436808374	0.949	0.913	5.92E-11	Granulocyte
S100A43	3.12E-15	0.792280392	0.758	0.501	6.65E-11	Granulocyte
AHNAK1	5.54E-15	0.784385917	0.528	0.29	1.18E-10	Granulocyte

AKAP132	1.99E-14	0.749165341	0.685	0.448	4.24E-10	Granulocyte
CTSD1	2.85E-14	0.593156868	0.478	0.242	6.09E-10	Granulocyte
KCNQ1OT	4.24E-14	1.383685288	0.36	0.16	9.06E-10	Granulocyte
RPL71	4.44E-14	0.521907738	0.904	0.86	9.48E-10	Granulocyte
NFE2L22	7.24E-14	0.913071352	0.517	0.289	1.55E-09	Granulocyte
FXYD51	1.45E-13	0.808484972	0.551	0.314	3.09E-09	Granulocyte
SGK12	3.13E-13	0.949718994	0.287	0.116	6.69E-09	Granulocyte
AP1S22	3.45E-13	0.864480486	0.292	0.12	7.37E-09	Granulocyte
TUBA1A1	4.84E-13	1.088105903	0.404	0.2	1.03E-08	Granulocyte
BIRC31	6.95E-13	1.059507864	0.489	0.278	1.48E-08	Granulocyte
LAPTM51	8.05E-13	0.589590005	0.579	0.313	1.72E-08	Granulocyte
PRKX1	8.09E-13	0.630218507	0.281	0.109	1.73E-08	Granulocyte
SOCS1	1.27E-12	0.747627525	0.326	0.141	2.72E-08	Granulocyte
NFKB11	1.31E-12	0.899851116	0.371	0.179	2.80E-08	Granulocyte
HES13	1.51E-12	1.033607641	0.382	0.183	3.22E-08	Granulocyte
RPL36AL2	1.64E-12	0.638122746	0.803	0.653	3.51E-08	Granulocyte
MYADM2	1.65E-12	0.926895027	0.461	0.259	3.53E-08	Granulocyte
RPL111	1.94E-12	0.344722406	0.961	0.954	4.15E-08	Granulocyte
SAT11	1.97E-12	0.337300633	0.893	0.66	4.21E-08	Granulocyte
VAMP82	2.28E-12	0.836089442	0.427	0.219	4.86E-08	Granulocyte
NR4A32	2.96E-12	0.825042117	0.281	0.114	6.32E-08	Granulocyte
RPS4X1	3.49E-12	0.410828991	0.938	0.921	7.45E-08	Granulocyte
DRAP1	5.86E-12	0.843148334	0.438	0.239	1.25E-07	Granulocyte
MAPRE1	1.12E-11	0.755107946	0.281	0.121	2.39E-07	Granulocyte
NDUFA42	1.91E-11	0.775583739	0.657	0.447	4.08E-07	Granulocyte
OSBPL8	3.18E-11	0.721955149	0.404	0.217	6.79E-07	Granulocyte
EMP33	3.42E-11	0.739249171	0.466	0.265	7.30E-07	Granulocyte
RPL261	5.48E-11	0.395457763	0.972	0.933	1.17E-06	Granulocyte
ITM2B3	6.67E-11	0.71587742	0.685	0.489	1.42E-06	Granulocyte
RPL24	6.90E-11	0.464045717	0.927	0.862	1.47E-06	Granulocyte
EIF3E1	8.43E-11	0.669265233	0.618	0.424	1.80E-06	Granulocyte
ZEB22	9.54E-11	0.666637988	0.433	0.228	2.04E-06	Granulocyte
CALM23	1.71E-10	0.687828141	0.691	0.506	3.65E-06	Granulocyte
ARHGDIB2	1.96E-10	0.578885943	0.579	0.344	4.18E-06	Granulocyte
BST21	2.12E-10	0.694083415	0.365	0.189	4.53E-06	Granulocyte
PABPC42	2.21E-10	0.978597458	0.489	0.316	4.71E-06	Granulocyte
RHBDD2	2.26E-10	0.609884759	0.264	0.114	4.83E-06	Granulocyte
NR4A12	2.45E-10	0.565480099	0.461	0.255	5.22E-06	Granulocyte
BHLHE401	2.47E-10	0.9064377	0.32	0.16	5.27E-06	Granulocyte
RPL142	2.65E-10	0.386751731	0.921	0.882	5.66E-06	Granulocyte
RPS241	2.75E-10	0.345876791	0.944	0.922	5.87E-06	Granulocyte
GPX43	6.57E-10	0.697445665	0.528	0.348	1.40E-05	Granulocyte
NR4A22	7.51E-10	0.741885409	0.444	0.254	1.60E-05	Granulocyte

EEF1A11	9.56E-10	0.452828317	0.949	0.952	2.04E-05	Granulocyte
SEC11A2	1.01E-09	0.738952853	0.444	0.273	2.16E-05	Granulocyte
RPL5	1.07E-09	0.336655087	0.933	0.861	2.28E-05	Granulocyte
RPS112	1.15E-09	0.316541969	0.972	0.899	2.45E-05	Granulocyte
BZW12	1.36E-09	0.789053819	0.449	0.277	2.91E-05	Granulocyte
CLIC12	3.10E-09	0.771832484	0.573	0.413	6.62E-05	Granulocyte
RPS201	4.25E-09	0.321816102	0.927	0.906	9.08E-05	Granulocyte
EGR12	5.63E-09	0.873777169	0.421	0.236	0.00012	Granulocyte
TUBA1B3	6.67E-09	0.625539684	0.517	0.335	0.000142	Granulocyte
TSC22D21	8.66E-09	0.714943389	0.264	0.126	0.000185	Granulocyte
RNF1451	1.58E-08	0.665132804	0.421	0.256	0.000337	Granulocyte
LRRFIP12	1.73E-08	0.546290375	0.64	0.455	0.000369	Granulocyte
PRDX13	1.80E-08	0.697061203	0.494	0.326	0.000383	Granulocyte
MT-ND21	2.08E-08	0.32755766	0.933	0.903	0.000444	Granulocyte
CREM2	3.23E-08	0.349825756	0.702	0.47	0.00069	Granulocyte
NEAT14	3.70E-08	0.44883448	0.916	0.846	0.000791	Granulocyte
IDS2	4.30E-08	0.612365779	0.455	0.286	0.000917	Granulocyte
RGS101	4.85E-08	0.660841614	0.376	0.221	0.001035	Granulocyte
BATF1	5.00E-08	0.28833162	0.331	0.165	0.001067	Granulocyte
SYTL32	1.01E-07	0.321093197	0.461	0.259	0.002151	Granulocyte
LDHB1	1.14E-07	0.728483545	0.455	0.295	0.002425	Granulocyte
RPL101	1.28E-07	0.263433344	0.994	0.987	0.002736	Granulocyte
SNHG81	1.39E-07	0.719055597	0.455	0.294	0.002956	Granulocyte
PRDX61	1.71E-07	0.856209583	0.365	0.229	0.003653	Granulocyte
DNAJA12	1.89E-07	0.638526726	0.584	0.415	0.004037	Granulocyte
RPL35A	2.16E-07	0.273116475	0.972	0.922	0.00462	Granulocyte
SERTAD12	2.22E-07	0.617259004	0.309	0.169	0.004731	Granulocyte
22-Sep	3.99E-07	0.477714397	0.331	0.188	0.008526	Granulocyte
HSP90B13	4.26E-07	0.608983028	0.551	0.387	0.009084	Granulocyte
ZBTB202	4.73E-07	0.555813086	0.348	0.201	0.010086	Granulocyte
PLK3	6.39E-07	0.559998896	0.32	0.183	0.013638	Granulocyte
VAPA2	6.89E-07	0.649588017	0.416	0.276	0.014698	Granulocyte
CDKN1A3	8.70E-07	0.588700571	0.393	0.249	0.01857	Granulocyte
ATF42	9.65E-07	0.462341429	0.562	0.4	0.020605	Granulocyte
TALDO12	1.07E-06	0.595627689	0.275	0.153	0.022943	Granulocyte
ELF12	1.30E-06	0.498354556	0.511	0.348	0.027669	Granulocyte
SH3BGRL2	2.00E-06	0.543369752	0.348	0.213	0.04268	Granulocyte
RPL42	2.11E-06	0.321816559	0.826	0.694	0.045123	Granulocyte
COX7C1	2.16E-06	0.358642809	0.809	0.675	0.046048	Granulocyte
MT-ND31	2.46E-06	0.406939119	0.865	0.78	0.052583	Granulocyte
MT-ND41	3.02E-06	0.314365075	0.961	0.926	0.064364	Granulocyte
RPL91	3.12E-06	0.28115724	0.921	0.883	0.066672	Granulocyte
NPC23	3.13E-06	0.365291075	0.427	0.273	0.066847	Granulocyte

YBX12	3.23E-06	0.461472725	0.713	0.601	0.069023	Granulocyte
CAVIN12	3.39E-06	0.291108046	0.253	0.129	0.07235	Granulocyte
PFDN51	4.30E-06	0.356771874	0.798	0.69	0.091785	Granulocyte
CHMP33	6.23E-06	0.532683884	0.281	0.163	0.132905	Granulocyte
AP2M12	6.86E-06	0.52368976	0.315	0.196	0.146427	Granulocyte
TSTD11	7.77E-06	0.397264844	0.275	0.155	0.165944	Granulocyte
PTPN71	8.31E-06	0.455137731	0.258	0.137	0.177318	Granulocyte
TNFAIP33	1.27E-05	0.537942751	0.59	0.443	0.271445	Granulocyte
PRKAR1A1	1.38E-05	0.620832554	0.275	0.163	0.295177	Granulocyte
IRS21	1.76E-05	0.375789988	0.264	0.15	0.374774	Granulocyte
ANKRD282	1.76E-05	0.540894593	0.354	0.225	0.376167	Granulocyte
SQSTM13	1.81E-05	0.570554611	0.483	0.363	0.385962	Granulocyte
NACA1	1.96E-05	0.34394507	0.837	0.74	0.419083	Granulocyte
S100A113	2.35E-05	0.395267807	0.657	0.512	0.502663	Granulocyte
RYBP	3.16E-05	0.458545893	0.27	0.158	0.674781	Granulocyte
TSC22D32	3.42E-05	0.444901524	0.725	0.606	0.729946	Granulocyte
JUNB2	3.89E-05	0.281629029	0.708	0.57	0.830039	Granulocyte
PDIA33	4.06E-05	0.47636952	0.331	0.218	0.866339	Granulocyte
TACC13	4.56E-05	0.364525337	0.343	0.219	0.972598	Granulocyte
ATP6V1F1	4.93E-05	0.621130211	0.348	0.236	1	Granulocyte
GADD45B3	5.35E-05	0.481853812	0.388	0.262	1	Granulocyte
ARL6IP51	7.77E-05	0.39170964	0.376	0.254	1	Granulocyte
H3F3A2	8.12E-05	0.271103007	0.803	0.711	1	Granulocyte
ATP6V0B1	9.25E-05	0.422452791	0.32	0.21	1	Granulocyte
CCNI1	0.000113	0.443765338	0.59	0.492	1	Granulocyte
DYNLL11	0.000121	0.515486048	0.601	0.48	1	Granulocyte
RBMX2	0.00014	0.477256328	0.522	0.405	1	Granulocyte
CBX31	0.000144	0.577829077	0.444	0.341	1	Granulocyte
HMGN1	0.000156	0.382793587	0.539	0.424	1	Granulocyte
RBM8A1	0.000175	0.407325956	0.444	0.328	1	Granulocyte
DBI3	0.0002	0.547665106	0.449	0.354	1	Granulocyte
MCL12	0.00022	0.28029047	0.601	0.476	1	Granulocyte
HSP90AB1	0.000227	0.343436603	0.764	0.641	1	Granulocyte
DDX52	0.00023	0.311847912	0.82	0.75	1	Granulocyte
ZNF3312	0.000244	0.576309108	0.365	0.246	1	Granulocyte
SH3BGRL3	0.000244	0.31371494	0.73	0.617	1	Granulocyte
DDIT41	0.000253	0.354802608	0.483	0.356	1	Granulocyte
ABRACL	0.000315	0.507669079	0.27	0.176	1	Granulocyte
ZFP363	0.00034	0.265770292	0.607	0.463	1	Granulocyte
ZFAND52	0.000382	0.367630593	0.416	0.298	1	Granulocyte
ARPC31	0.000417	0.30818421	0.539	0.424	1	Granulocyte
PABPC12	0.000418	0.315349942	0.82	0.744	1	Granulocyte
BRD21	0.000512	0.289588707	0.444	0.328	1	Granulocyte

RHOH1	0.000616	0.266005878	0.275	0.172	1	Granulocyte
ATP5MC21	0.000697	0.271050209	0.669	0.546	1	Granulocyte
SKP12	0.000804	0.277072218	0.556	0.442	1	Granulocyte
CIRBP	0.000828	0.293740746	0.584	0.462	1	Granulocyte
STK17B1	0.00084	0.306957844	0.371	0.258	1	Granulocyte
STMP11	0.000851	0.413139398	0.253	0.168	1	Granulocyte
MTPN2	0.000854	0.391179185	0.32	0.225	1	Granulocyte
TAGLN22	0.000962	0.28823569	0.466	0.355	1	Granulocyte
C4orf483	0.00102	0.459005458	0.287	0.198	1	Granulocyte
SERPINB12	0.001025	0.478910122	0.27	0.183	1	Granulocyte
CAMLG	0.00113	0.378047218	0.281	0.192	1	Granulocyte
HSPB14	0.001181	0.785683979	0.331	0.446	1	Granulocyte
ARID1B	0.001418	0.49907533	0.292	0.205	1	Granulocyte
UQCRH1	0.001479	0.483184164	0.494	0.432	1	Granulocyte
TAOK3	0.001502	0.440940598	0.264	0.181	1	Granulocyte
POMP3	0.001532	0.308346005	0.483	0.379	1	Granulocyte
AP2B12	0.001554	0.369486776	0.32	0.225	1	Granulocyte
PEBP12	0.001647	0.516009793	0.376	0.289	1	Granulocyte
EIF3D	0.00193	0.341594165	0.292	0.208	1	Granulocyte
ARL6IP11	0.002047	0.39792762	0.376	0.28	1	Granulocyte
SRRM12	0.002233	0.307780822	0.506	0.402	1	Granulocyte
ATP1B33	0.002474	0.262649018	0.449	0.343	1	Granulocyte
IER23	0.002534	0.34840687	0.433	0.337	1	Granulocyte
RPL36A1	0.002597	0.353712398	0.674	0.606	1	Granulocyte
SSBP11	0.002618	0.255399112	0.287	0.201	1	Granulocyte
TMED22	0.002897	0.337194862	0.343	0.255	1	Granulocyte
EIF1B1	0.003118	0.374791398	0.326	0.24	1	Granulocyte
DDX3X1	0.003553	0.324755991	0.461	0.358	1	Granulocyte
HNRNPA2	0.003919	0.2678503	0.534	0.438	1	Granulocyte
EIF3M	0.004093	0.306141554	0.253	0.174	1	Granulocyte
KHDRBS1	0.004453	0.266991876	0.303	0.221	1	Granulocyte
BTG22	0.004816	0.391212792	0.348	0.266	1	Granulocyte
MYL12A2	0.004917	0.277743032	0.551	0.465	1	Granulocyte
FOS2	0.005674	0.439057068	0.59	0.502	1	Granulocyte
ANXA112	0.005938	0.304070618	0.27	0.196	1	Granulocyte
RPS27L2	0.006196	0.306526297	0.478	0.381	1	Granulocyte
ARL6IP4	0.006336	0.302289765	0.449	0.368	1	Granulocyte
CSNK1A11	0.00759	0.349766376	0.354	0.279	1	Granulocyte
ATP6V0E12	0.008197	0.300805361	0.427	0.346	1	Granulocyte
REX1BD	0.008672	0.258631037	0.27	0.197	1	Granulocyte
